# Synthesis and Toxicity Evaluation of Some *N*^4^-Aryl Substituted 5-Trifluoromethoxyisatin-3-thiosemicarbazones

**DOI:** 10.3390/molecules16086408

**Published:** 2011-07-29

**Authors:** Humayun Pervez, Naveeda Saira, Mohammad Saeed Iqbal, Muhammad Yaqub, Khalid Mohammed Khan

**Affiliations:** 1 Department of Chemistry, Bahauddin Zakariya University, Multan-60800, Pakistan; 2 Department of Chemistry, FC College, Lahore-54600, Pakistan; 3 H.E.J. Research Institute of Chemistry, International Center for Chemical and Biological Sciences, University of Karachi, Karachi-75270, Pakistan

**Keywords:** isatin, 5-trifluoromethoxyisatin, thiosemicarbazones, 5-trifluoromethoxyisatin-3-thiosemicarbazones, toxicity

## Abstract

A series of twenty one *N*^4^-aryl substituted 5-trifluoromethoxyisatin-3-thiosemicarbazones **3a-3u** was synthesized by the reaction of trifluoromethoxyisatin **1** with different arylthiosemicarbazides **2** in aqueous ethanol (50%), containing a few drops of acetic acid. Their structures were established on the basis of analytical (CHN) and spectral (IR, ^1^H-NMR, EIMS) data. All the synthesized compounds were evaluated for their toxicity potential by a brine shrimp lethality bioassay. Ten compounds *i.e*., **3a**, **3e**, **3i**-**3l** and **3n**-**3q** proved to be active in this assay, displaying promising toxicity (LD_50_ = 1.11 × 10^−5^ M − 1.80 × 10^−4^ M). Amongst these, **3k**, **3n** and **3o** were found to be the most active ones (LD_50_ = 1.11 × 10^−5^ M − 1.43 × 10^−5^ M). Compound **3k** showed the highest activity with a LD_50_ value of 1.11 × 10^−5^ M and can, therefore, be used as a lead for further studies. Structure-activity relationship (SAR) studies revealed that the presence of strong inductively electron-attracting trifluoromethoxy substituent at position-5 of the isatin moiety played an important role in inducing or enhancing toxic potentiality of some of the synthesized compounds.

## 1. Introduction

Much interest has been shown in isatin and its derivatives due to their diversified pharmacological properties including antimicrobial, anticonvulsant, antineoplastic, antiviral, antihypertensive, anti-inflammatory and enzymatic inhibition activities [1,2,3,4,5,6,7,8,9,10,11,12,13,14,15,16,17,18]. Amongst isatin derivatives, isatin-thiosemicarbazones have been found to demonstrate numerous chemotherapeutic properties such as anticancer, antimicrobial, antituberculosis, antiulcer, antiviral, antiplasmodial, cytotoxic and enzymatic inhibition [1,2,3,4,5,6,8,9,12,19,20]. Prompted by this and in continuation of our work on bioactive isatin derivatives [21,22,23,24,25], it was of interest to synthesize some new isatin-3-thiosemicarbazones, which may exhibit better or different types of biological properties. It is pertinent to mention that a number of *N*^4^-aryl substituted isatin-derived thiosemicarbazones, prepared earlier in our laboratory, displayed different biological activities such as antibacterial, antifungal, cytotoxic, phytotoxic and urease inhibition [22,23,24,25]. Structure-activity relationship (SAR) studies in the synthesized *N*^4^-aryl substituted isatin-3-thiosemicarbazones revealed that in certain cases, the nature and position of different substituents about the phenyl ring attached to N^4^ of the thiosemicarbazone moiety [22,23,25] and/or the presence of an inductively electron-withdrawing nitro group at position-5 of the isatin scaffold [24] played an important role in the inducement or enhancement of different activities. Furthermore, it has been reported by some other workers that certain *N*^4^-aryl substituted 5-nitroisatin-3-thiosemicabazones exhibit more cytotoxic activity than their alkyl and alkenyl counterparts [26]. Also, *N*^4^-aryl substituted 5-bromoisatin-3-thiosemicabazones have been found to show favourable cytotoxicity [27]. In view of this, it was envisaged that the combined effect of substitution of different inductively electron-attracting groups at position-5 of the isatin part and the attachment of a variety of aryl substituents to N^4^ of the thiosemicarbazone moiety would result in increased toxic activity. Thus, the present work to synthesize some title thiosemicarbazones and screen them for their toxicity potential by a brine shrimp (*Artemia salina*) lethality bioassay was accomplished. This work describes the effects of the nature of aryl groups (modified by placement of one, two, or three substituents about the phenyl ring) attached to N^4^ of the thiosemicarbazone moiety as well as the presence of trifluoromethoxy substituent at position-5 of the isatin scaffold on the toxicity potential of these compounds.

## 2. Results and Discussion

This study illustrates the synthesis and *in vitro* determination of the toxic effects of nineteen new and two previously reported [15] *N*^4^-aryl substituted 5-trifluoromethoxyisatin-3-thiosemicarbazones **3b**-**3d**, **3f**-**3u** and **3a**, **3e**, respectively.

### 2.1. Chemistry

**Scheme 1:**
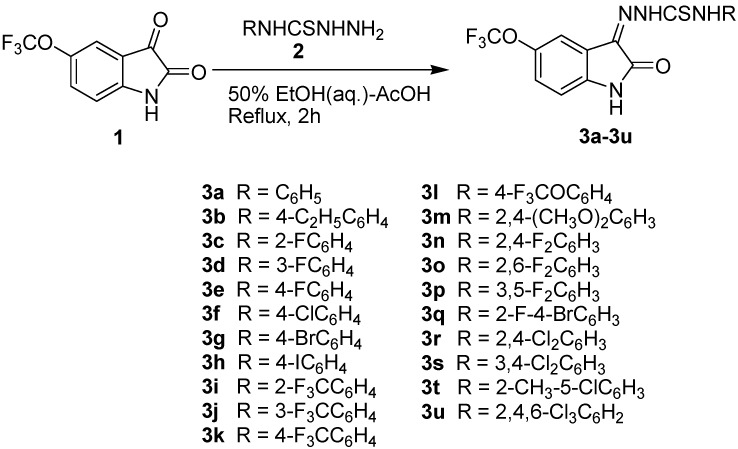
The synthetic route to the title compounds **3a-3u**.

5-Trifluoromethoxyisatin **1** was reacted with appropriate N-substituted thiosemicarbazides **2** in aqueous ethanol (50%) containing a catalytic amount of glacial acetic acid to give the corresponding 5-trifluoromethoxyisatin-3-thiosemicarbazones **3a-3u** (Scheme 1) in moderate to excellent yields (50–93%). The structures of all the synthesized compounds were confirmed by means of their analytical (CHN) and spectral (IR, ^1^H-NMR, EIMS) data. Satisfactory elemental analysis (±0.4% of theoretical values) was obtained for all compounds, except where noted otherwise. The IR spectra of **3a**-**3u** showed the absorption bands of NH stretching in the 3354–3200 and 3196–3055 cm^−1^ regions. The absorption bands of lactam C=O, azomethine C=N and thioamide C=S stretchings appeared in the 1705–1692, 1629–1564 and 1182–1143 cm^−1^ regions, respectively [26,28,29,30]. The ^1^H-NMR spectra of **3a**-**3u** displayed three separate singlets at *δ* 10.45–11.07, *δ* 11.39–11.48 and *δ* 12.58–12.81 attributed to thiosemicarbazone N^4^-H, indole NH and thiosemicarbazone N^2^-H, respectively [26,28,31,32]. The electron impact mass spectra (EIMS) of the synthesized thiosemicarbazones demonstrated molecular ions of different intensities, confirming their molecular weights. Compounds **3r** and **3u** did not display molecular ion peaks in their mass spectra; however, the fragments corresponding to thiosemicarbazone part of the molecules, formed by N-N and NH-CS bond rupture, confirmed their structures. X-ray structures of two representative examples **3k** and **3t** were determined in order to confirm the assigned structures and establish conformations of the synthesized thiosemicarbazones **3a**-**3u**. Relevant crystal data and details of structural elucidation have been reported elsewhere [33,34].

### 2.2. Toxicity

All the synthesized thiosemicarbazones **3a-3u** were screened for toxic activity by a brine shrimp (*Artemia salina*) lethality bioassay. The compound 2-(2-oxo-1,2-dihydro-3*H*-indol-3-ylidene)-*N*-phenyl-1-hydrazinecarbothioamide, whose synthesis has been reported elsewhere [21], served as reference compound (without a substituent in the isatin part as well as on the phenyl ring) to evaluate the effects of substituents about the isatin scaffold as well as the phenyl ring attached to N^4^ of the thiosemicarbazone moiety of the test compounds on their toxicity potential. The results given in the Table 1 revealed that substitution of trifluoromethoxy group alone in the isatin scaffold or in combination with some other functions on the phenyl ring resulted into either induction or enhancement of toxic activity in certain cases, compared with the reference compound. To the contrary, in some other cases, such a combination caused either elimination or reduction in the activity. This inference receives support from the results obtained by us in the earlier studies [22,23,25]. For example, compound **3a** possessing inductively electron-attracting trifluoromethoxy substituent at position-5 of the isatin scaffold exhibited promising toxic activity (LD_50_ = 4.49 × 10^−5^ M), whereas the corresponding compound *i.e.*, the reference point having no trifluoromethoxy group in the isatin moiety, was found to be inactive (LD_50_ = >3.38 × 10^−4^ M) [22]. Similarly, compound **3k** having trifluoromethyl substituent at position-4 of the phenyl ring was found to display enhanced toxicity (LD_50_ = 1.11 × 10^−5^ M) when compared with the corresponding compound with no trifluoromethoxy function at position-5 of the isatin scaffold, giving a LD_50_ value of 1.17 × 10^−4^ M [23]. Also, compounds **3n** and **3o** possessing fluoro groups at positions-2,4 and -2,6 of the phenyl ring exhibited increased toxic activity (LD_50_ = 1.43 × 10^−5^ M and 1.34 × 10^−5^ M, respectively) in comparison to the corresponding compounds having no trifluoromethoxy substituent at position-5 of the isatin part, giving the same LD_50_ values of 2.00 × 10^−5^ M. Much more pronounced enhancement was observed in the case of **3k** (LD_50_ = 1.17 × 10^−4^ M →1.11 × 10^−5^ M). However, compound **3l** with trifluoromethoxy group at position-4 of the phenyl ring attached to N^4^ of the thiosemicarbazone moiety and the corresponding compound possessing no such substituent at position-5 of the isatin scaffold displayed almost the same activity (LD_50_ = 1.81 × 10^−5^ M and 1.80 × 10^−^5 M, respectively) [23] indicating that the introduction of trifluoromethoxy group at position-5 of the isatin scaffold did not significantly affect toxicity potential of the compound having trifluoromethyl substituent at position-4 of the phenyl ring. On the contrary, compounds **3b**-**3d**, **3h**, **3r** and **3u** with 4-ethyl, 2-fluoro, 3-fluoro, 4-iodo, 2,4-dichloro and 2,4,6-trichloro substituents about the phenyl ring showed almost no toxic effects (LD_50_ = >2.45 × 10^−4^ M, >2.51 × 10^−4^ M, >2.51 × 10^−4^ M, >1.98 × 10^−4^ M, >2.23 × 10^−4^ M and >2.07 × 10^−4^ M, respectively), whereas the corresponding compounds without trifluoromethoxy group at position-5 of the isatin scaffold gave LD_50_ values of 5.10 × 10^−5^ M, 3.10 × 10^−5^ M, 4.60 × 10^−5^ M, 1.26 × 10^−4^ M, 1.20 × 10^−4^ M and 1.10 × 10^−5^ M, respectively [23,25]. Similarly, compounds **3i**, **3j** and **3p** with 2-trifluoromethyl, 3-trifluoromethyl and 3,5-difluoro substituents about the phenyl ring showed reduced toxic activity (LD_50_ = 1.80 × 10^−4^ M, 1.70 × 10^−4^ M and 4.32 × 10^−5^ M, respectively) in comparison to the corresponding compounds (without trifluoromethoxy group at position-5 of the isatin moiety), which gave LD_50_ values of 1.36 × 10^−4^ M, 2.20 × 10^−5^ M and 2.10 × 10^−5^ M [23]. Relatively, much pronounced reduction in the toxic activity was observed in the case of **3j** (LD_50_ = 2.20 × 10^−5^ M → 1.70 × 10^−4^ M). This showed that the simultaneous presence of inductively electron-attracting groups in the isatin scaffold as well as the phenyl ring substituted at N^4^ of the thiosemicarbazone moiety caused either elimination or reduction in the toxicity potential. However, compared with monofluoro-substituted compounds **3c** and **3d**, compound **3e** having fluoro substituent at position-4 of the phenyl ring displayed promising activity (LD_50_ = 3.72 × 10^−5^ M) in the present assay. Similarly, compared with compound **3c**, the dihalogeno-substituted compound **3q** possessing fluoro and bromo substituents at positions-2 and -4 of the phenyl ring, respectively, exhibited induced toxic activity (LD_50_ = 1.60 × 10^−4^ M) in the present assay.

On the whole, out of twenty one compounds tested for toxic activity, ten *i.e.*,**3a**, **3e**, **3i-3l** and **3n**-**3q** proved to be active, exhibiting promising toxicity (LD_50_ = 1.11 × 10^−5^ M − 1.80 × 10^−4^ M) against *Artemia salina.* The remaining compounds *i.e*., **3b-3d**, **3f-3h**, **3m** and **3r-3u** gave LD_50_ values ranging from >1.98 × 10^−4^ M to >2.51 × 10^−4^ M in the present assay and, therefore, can be considered as inactive. Compound **3k** having an inductively electron-attracting trifluoromethyl substituent at position-4 of the phenyl ring proved to be the most potent toxicant, as it showed exciting activity with a LD_50_ value of 1.11 × 10^−5^ M, which was even better than the potent anticancer drug etoposide (LD_50_ = 1.27 × 10^−5^ M) used as a reference standard in the present assay, and thus seems a promising lead compound for further studies. The next potent compounds were found to be **3n** and **3o** with fluoro groups at positions-2,4 and -2,6 of the phenyl ring, showing toxic activity with LD_50_ values of 1.43 × 10^−5^ M and 1.34 × 10^−5^ M, respectively. These preliminary results indicate that structural modifications may lead to the development of novel compounds with improved toxicity. 

**Table 1:** Brine shrimp (*Artemia salina*) bioassay of compounds **3a-3u**.

Compounds	LD_50_ (M)
**3a**	4.49 × 10^−5^
**3b**	>2.45 × 10^−4^
**3c**	>2.51 × 10^−4^
**3d**	>2.51 × 10^−4^
**3e**	3.72 × 10^−5^
**3f**	>2.42 × 10^−4^
**3g**	>2.18 × 10^−4^
**3h**	>1.98 × 10^−4^
**3i**	1.80 × 10^−4^
**3j**	1.70 × 10^−4^
**3k**	1.11 × 10^−5^
**3l**	1.81 × 10^−5^
**3m**	>2.27 × 10^−4^
**3n**	1.43 × 10^−5^
**3o**	1.34 × 10^−5^
**3p**	4.32 × 10^−5^
**3q**	1.60 × 10^−4^
**3r**	>2.23 × 10^−4^
**3s**	>2.23 × 10^−4^
**3t**	>2.34 × 10^−4^
**3u**	>2.07 × 10^−4^
2-(2-Oxo-1,2-dihydro-3*H*-indol-3-ylidene)-*N*-phenyl-1-hydrazinecarbothioamide *	>3.38 × 10^−4^

* [21,22].

## 3. Experimental

### 3.1. General

Melting points were taken on a Fisher-Johns melting point apparatus and are uncorrected. Elemental analyses were performed on a Leco CHNS-9320 (USA) elemental analyzer. Infrared spectra (KBr discs) were run on Shimadzu Prestige-21 FT-IR spectrometer. The ^1^H-NMR spectra were recorded in DMSO-*d*_6_ on Bruker (Rhenistetten-Forchheim, Germany) AM 300 spectrometer, operating at 300 MHz and using TMS as an internal standard. ^1^H chemical shifts are reported in δ/ppm and coupling constants in Hz. The electron impact mass spectra (EIMS) were determined with JEOL MSRoute mass spectrometer. The progress of the reaction and purity of the products were checked on TLC plates coated with Merck silica gel 60 GF_254_, and the spots were visualized under ultraviolet light at 254/366 nm and/or spraying with iodine vapours. *In vitro* biological evaluation of the synthesized compounds was done at Panjwani Center for Molecular Medicine and Drug Research, H.E.J. Research Institute of Chemistry, University of Karachi, Pakistan.

### 3.2. Synthesis

#### 3.2.1. General Procedure for the Preparation of 5-Ttrifluoromethoxyisatin-thiosemicarbazones **3a-3u**

To a solution of 5-trifluoromethoxyisatin (2.5 mmol) in 50% aqueous ethanol (10 mL) containing a catalytic amount of glacial acetic acid was added the appropriate thiosemicarbazide (2.5 mmol) dissolved in ethanol (10 mL) under stirring. The reaction mixture was then heated under reflux for 2 h. The crystalline or amorphous solid formed during heating was collected by suction filtration. Thorough washing with hot aqueous ethanol (50%) furnished the target compounds **3a-3u** in pure form.

*N-Phenyl-2-[2-oxo-5-(trifluoromethoxy)-1,2-dihydro-3H-indol-3-ylidene]-1-hydrazinecarbothioamide* (**3a**). Yield 93% as yellow fluffy crystals, mp: 230–232 °C (229–232 °C, lit. [15]). IR: 3319, 3178, 3119 (NH stretching), 1695 (C=O), 1629 (C=N), 1143 (C=S) cm^−1^. ^1^H-NMR (DMSO-*d*_6_): δ 7.04 (d, *J* = 8.4 Hz, 1H, indole C_7_-H), 7.30 (t, *J* = 7.5 Hz, 1H, phenyl C_4_-H), 7.38 (dd, *J* = 8.4, 1.8 Hz, 1H, indole C_6_-H), 7.45 (t, *J* = 7.5 Hz, 2H, phenyl C_3_-H, C_5_-H), 7.59 (d, *J* = 8.1 Hz, 2H, phenyl C_2_-H, C_6_-H), 7.81 (d, *J* = 1.8 Hz, 1H, indole C_4_-H), 10.90 (s, 1H, CS-NH ), 11.41 (s, 1H, indole NH), 12.66 (s, 1H, N-NH). MS, *m/z* (rel. int.): 380 (M^+^, 42), 352 (100), 287 (13), 263 (10), 245 (66), 229 (15), 217 (18), 216 (20), 202 (19), 201 (22), 188 (52), 159 (16), 150 (8), 135 (71), 118 (24), 104 (6), 93 (38). Anal calcd. for C_16_H_11_F_3_N_4_O_2_S: C, 50.53, H, 2.92, N, 14.73; found: C, 50.47, H, 2.92, N, 15.01.

*N-(4-Ethylphenyl)-2-[2-oxo-5-(trifluoromethoxy)-1,2-dihydro-3H-indol-3-ylidene]-1-hydrazinecarbothioamide* (**3b**). Yield 83% as yellow fluffy crystals, mp: 258 °C. IR: 3200, 3150, 3067 (NH stretching), 1701 (C=O), 1597 (C=N), 1150 (C=S) cm^−1^. ^1^H-NMR (DMSO-*d_6_*): δ 1.21 (t, *J* = 7.5 Hz, 3H, CH_3_), 2.63 (q, *J* = 7.5 Hz, 2H, CH_2_), 7.03 (d, *J* = 8.4 Hz, 1H, indole C_7_-H), 7.27 (d, *J* = 8.1 Hz, 2H, phenyl C_2_-H, C_6_-H), 7.37 (d, *J* = 8.4 Hz, 1H, indole C_6_-H), 7.48 (d, *J* = 8.4 Hz, 2H, phenyl C_3_-H, C_5_-H), 7.81 (s, 1H, indole C_4_-H), 10.85 (s, 1H, CS-NH), 11.42 (s, 1H, indole NH), 12.63 (s, 1H, N-NH). MS, *m/z* (rel. int.): 408 (M^+^, 34), 380 (100), 365 (5), 311 (7), 245 (11), 230 (13), 217 (7), 216 (6), 202 (10), 188 (7), 163 (11), 131 (19), 106 (24), 77 (5). Anal calcd. for C_18_H_15_F_3_N_4_O_2_S: C, 52.94, H, 3.70, N, 13.72; found: C, 52.85, H, 3.69, N, 13.69.

*N-(2-Fluorophenyl)-2-[2-oxo-5-(trifluoromethoxy)-1,2-dihydro-3H-indol-3-ylidene]-1-hydrazinecarbothioamide* (**3c**). Yield 76% as yellow powder, mp: 225 °C. IR: 3289, 3264, 3073 (NH stretching), 1703 (C=O), 1607 (C=N), 1155 (C=S) cm^−1^. ^1^H-NMR (DMSO-*d_6_*): δ 7.04 (d, *J* = 8.7 Hz, 1H, indole C_7_-H), 7.27–7.52 (m, 5H, indole C_6_-H, phenyl C_3_-H, C_4_-H, C_5_-H, C_6_-H), 7.73 (s, 1H, indole C_4_-H), 10.81 (s, 1H, CS-NH ), 11.43 (s, 1H, indole NH), 12.71 (s, 1H, N-NH). MS, *m/z* (rel. int.): 398 (M^+^, 54), 371 (28), 370 (100), 351 (2), 301 (12), 263 (8), 245 (12), 234 (11), 216 (11), 188 (13), 154 (16), 134 (15), 111 (11), 83 (7), 69 (7), 44 (3). Anal calcd. for C_16_H_10_F_4_N_4_O_2_S: C, 48.24, H, 2.53, N, 14.07; found: C, 48.05, H 2.52, N 14.04.

*N-(3-Fluorophenyl)-2-[2-oxo-5-(trifluoromethoxy)-1,2-dihydro-3H-indol-3-ylidene]-1-hydrazinecarbothioamide* (**3d**). Yield 81% as yellow powder, mp: 238 °C. IR: 3289, 3264, 3073(NH stretching), 1701 (C=O), 1597 (C=N), 1161 (C=S) cm^−1^. ^1^H-NMR (DMSO-*d_6_*): δ 7.03 (d, *J* = 8.4 Hz, 1H, indole C_7_-H), 7.11–7.18 (m, 1H, phenyl C_4_-H), 7.38 (dd, *J* = 8.4, 1.5 Hz, 1H, indole C_6_-H), 7.49 (t, *J* = 9.9 Hz, 2H, phenyl C_6_-H, C_5_-H), 7.61 (d, *J* = 10.8 Hz, 1H, phenyl C_2_-H), 7.80 (s, 1H, indole C_4_-H), 10.94 (s, 1H, CS-NH), 11.45 (s, 1H, indole NH), 12.72 (s, 1H, N-NH). MS, *m/z* (rel. int.): 398 (M^+^, 9), 370 (27), 338 (7), 312 (3), 284 (46), 269 (3), 256 (45), 241 (27), 230 (31), 213 (29), 202 (26), 185 (39), 171 (22), 157 (25), 136 (46), 129 (65), 111 (45), 85 (36), 73 (100), 69 (36), 61 (25), 60 (73), 57 (76). Anal calcd. for C_16_H_10_F_4_N_4_O_2_S: C, 48.24, H, 2.53, N, 14.07; found: C, 48.17, H, 2.53, N, 14.05.

*N-(4-Fluorophenyl)-2-[2-oxo-5-(trifluoromethoxy)-1,2-dihydro-3H-indol-3-ylidene]-1-hydrazinecarbothioamide* (**3e**). Yield 76% as yellow fluffy crystals, mp: 224–226 °C (224–227 °C, lit. [15]). IR: 3320, 3197 (NH stretching), 1692 (C=O), 1600 (C=N), 1155 (C=S) cm^−1^. ^1^H-NMR (DMSO-*d_6_*): δ 7.03 (d, *J* = 8.7 Hz, 1H, indole C_7_-H), 7.26–7.32 (m, 2H, phenyl C_2_-H, C_6_-H), 7.37 (dd, *J* = 8.7, 2.1 Hz, 1H, indole C_6_-H), 7.57–7.61 (m, 2H, phenyl C_3_-H, C_5_-H), 7.78 (s, 1H, indole C_4_-H), 10.91 (s, 1H, CS-NH), 11.43 (s, 1H, N-NH), 12.66 (s, 1H, indole NH). MS, *m/z* (rel. int.): 398 (M^+^, 24), 370 (100), 364 (28), 338 (12), 301 (9), 287 (7), 263 (8), 243 (63), 230 (45), 215 (39), 202 (31), 187 (30), 136 (81), 133 (31), 109 (27), 95 (15), 83 (12), 69 (27), 52 (5), 44 (25). Anal calcd. for C_16_H_10_F_4_N_4_O_2_S: C, 48.24, H, 2.53, N, 14.07; found: C, 48.10, H, 2.52, N, 14.12.

*N-(4-Chlorophenyl)-2-[2-oxo-5-(trifluoromethoxy)-1,2-dihydro-3H-indol-3-ylidene]-1-hydrazinecarbothioamide* (**3f**). Yield 75% as shinning yellow powder, mp: 235 °C. IR: 3258, 3107, 3080 (NH stretching), 1701 (C=O), 1589 (C=N), 1150 (C=S) cm^−1^. ^1^H-NMR (DMSO-*d_6_*): δ 7.03 (d, *J* = 8.1 Hz, 1H, indole C_7_-H), 7.38 (d, *J* = 7.5 Hz, 1H, indole C_6_-H), 7.51 (d, *J* = 7.5 Hz, 2H, phenyl C_2_-H, C_6_-H), 7.65 (d, *J* = 7.2 Hz, 2H, phenyl C_3_-H, C_5_-H), 7.78 (s, 1H, indole C_4_-H), 10.93 (s, 1H, CS-NH), 11.44 (s, 1H, indole NH), 12.70 (s, 1H, N-NH). MS, *m/z* (rel. int.): 416/414 (M^+^, 20/53), 388/386 (39/100), 287 (32), 247/245 (8/13), 230/228 (18/15), 217/215 (8/7), 198/196 (5/5), 171 (17), 152 (12), 127 (31), 92 (4). Anal calcd. for C_16_H_10_ClF_3_N_4_O_2_S: C, 46.33, H, 2.43, N, 13.51; found: C, 46.34, H, 2.43, N, 13.51.

*N-(4-Bromophenyl)-2-[2-oxo-5-(trifluoromethoxy)-1,2-dihydro-3H-indol-3-ylidene]-1-hydrazinecarbothioamide* (**3g**). Yield 70% as yellow powder, mp: 240 °C. IR: 3281, 3088 (NH stretching), 1699 (C=O), 1600 (C=N), 1148 (C=S) cm^−1^. ^1^H-NMR (DMSO-*d_6_*): δ 7.04 (d, *J* = 8.7 Hz, 1H, indole C_7_-H), 7.39 (d, *J* = 7.8 Hz, 1H, indole C_6_-H), 7.60 (d, *J* = 9.0 Hz, 2H, phenyl C_2_-H, C_6_-H), 7.64 (d, *J* = 8.7 Hz, 2H, phenyl C_3_-H, C_5_-H), 7.79 (s, 1H, indole C_4_-H), 10.92 (s, 1H, CS-NH), 11.44 (s, 1H, indole NH), 12.70 (s, 1H, N-NH). MS, *m/z* (rel. int.): 460/458 (M^+^, 5/5), 432/430 (7/5), 354 (5), 287 (45), 262 (5), 245 (36), 230/228 (5/5), 215/213 (20/20), 199/197 (39/28), 173/171 (100/96), 148 (9), 134 (4), 92 (13), 65 (19). Anal calcd. for C_16_H_10_BrF_3_N_4_O_2_S: C, 41.85, H, 2.19, N, 12.20; found: C, 42.00, H, 2.19, N, 12.19.

*N-(4-Iodophenyl)-2-[2-oxo-5-(trifluoromethoxy)-1,2-dihydro-3H-indol-3-ylidene]-1-hydrazinecarbothioamide* (**3h**). Yield 80% as yellow powder, mp: 246 °C. IR: 3291, 3196, 3080 (NH stretching), 1703 (C=O), 1591 (C=N), 1146 (C=S) cm^−1^. ^1^H-NMR (DMSO-*d_6_*): δ 7.03 (d, *J* = 8.7 Hz, 1H, indole C_7_-H), 7.38 (d, *J* = 8.1 Hz, 1H, indole C_6_-H), 7.45 (d, *J* = 8.4 Hz, 2H, phenyl C_2_-H, C_6_-H), 7.79 (d, *J* = 8.1 Hz, 3H, indole C_4_-H , phenyl C_3_-H, C_5_-H), 10.89 (s, 1H, CS-NH ), 11.44 (s, 1H, indole NH), 12.70 (s, 1H, N-NH). MS, *m/z* (rel. int.): 506 (M^+^, 32), 478 (69), 352 (5), 287 (100), 261 (95), 254 (30), 245 (23), 230 (56), 229 (49), 228 (49), 220 (35), 219 (100), 202 (39), 159 (7), 127 (26), 92 (72), 64 (24). Anal calcd. for C_16_H_10_F_3_IN_4_O_2_S: C, 37.96, H, 1.99, N, 11.07; found: C, 37.92, H, 1.99, N, 11.04.

*2-[2-Oxo-5-(trifluoromethoxy)-1,2-dihydro-3H-indol-3-ylidene]-N-[2-(trifluoromethyl)phenyl]-1-hydrazinecarbothioamide* (**3i**). Yield 87% as yellow fluffy crystals, mp: 234 °C. IR: 3346, 3188, 3171(NH stretching), 1695 (C=O), 1625 (C=N), 1182 (C=S) cm^−1^. ^1^H-NMR (DMSO-*d_6_*): δ 7.03 (d, *J* = 8.4 Hz, 1H, indole C_7_-H), 7.38 (dd, *J* = 8.4, 1.8 Hz, 1H, indole C_6_-H), 7.57 (d, *J* = 8.1 Hz, 1H, phenyl C_4_-H), 7.63 (d, *J* = 7.5 Hz, 1H, phenyl C_6_-H), 7.72 (s, 1H, indole C_4_-H), 7.77-7.86 (m, 2H, phenyl C_3_-H, C_5_-H), 10.89 (s, 1H, CS-NH), 11.43 (s, 1H, indole NH), 12.71 (s, 1H, N-NH). MS, *m/z* (rel. int.): 448 (M^+^, 3), 420 (23), 414 (38), 388 (4), 345 (9), 311 (4), 243 (63), 228 (73), 215 (58), 202 (36), 187 (76), 186 (89), 167 (33), 166 (100), 159 (38), 145 (16), 139 (13), 133 (35), 128 (21), 114 (19), 83 (46), 78 (12), 69 (36), 44 (53). Anal calcd. for C_17_H_10_F_6_N_4_O_2_S: C, 45.54, H, 2.25, N, 12.50; found: C, 45.53, H, 2.24, N, 12.50.

*2-[2-Oxo-5-(trifluoromethoxy)-1,2-dihydro-3H-indol-3-ylidene]-N-[3-(trifluoromethyl)phenyl]-1-hydrazinecarbothioamide* (**3j**). Yield 79% as yellow powder, mp: 220 °C. IR: 3310, 3196, 3063 (NH stretching), 1699 (C=O), 1600 (C=N), 1161 (C=S) cm^−1^. ^1^H-NMR (DMSO-*d_6_*): *δ* 7.04 (d, *J* = 8.4 Hz, 1H, indole C_7_-H), 7.40 (dd, *J* = 8.7, 1.8 Hz, 1H, indole C_6_-H), 7.64–7.72 (m, 2H, phenyl C_5_-H, C_6_-H), 7.78 (s, 1H, indole C_4_-H), 7.99 (d, *J* = 7.2 Hz, 1H, phenyl C_4_-H), 8.04 (s, 1H, phenyl C_2_-H), 11.06 (s, 1H, CS-NH), 11.47 (s, 1H, indole NH), 12.76 (s, 1H, N-NH). MS, *m/z* (rel. int.): 448 (M^+^, 3), 420 (12), 414 (33), 395 (8), 388 (23), 372 (4), 346 (4), 345 (4), 330 (16), 277 (3), 243 (49), 230 (40), 228 (89), 216 (13), 215 (60), 203 (41), 202 (47), 188 (33), 187 (96), 186 (100), 167 (25), 161 (23), 160 (31), 159 (98), 145 (66), 139 (20), 133 (57), 109 (14), 105 (13), 95 (14), 85 (31), 83 (46), 82 (17), 80 (18), 69 (44), 63 (16), 44 (79). Anal calcd. for C_17_H_10_F_6_N_4_O_2_S: C, 45.54, H, 2.25, N, 12.50; found: C, 45.50, H, 2.24, N, 12.48.

*2-[2-Oxo-5-(trifluoromethoxy)-1,2-dihydro-3H-indol-3-ylidene]-N-[4-(trifluoromethyl)phenyl]-1-hydrazinecarbothioamide* (**3k**). Yield 75% as yellow powder, mp: 238–240 °C. IR: 3318, 3161, 3130 (NH stretching), 1701 (C=O), 1587 (C=N), 1167 (C=S) cm^−1^. ^1^H-NMR (DMSO-*d_6_*): *δ* 7.04 (d, *J* = 8.7 Hz, 1H, indole C_7_-H), 7.39 (dd, *J* = 9.9 Hz, 1H, indole C_6_-H), 7.82 (d, *J* = 9.3 Hz, 3H, indole C_4_-H, phenyl C_2_-H, C_6_-H ), 7.93 (d, *J* = 8.4 Hz, 2H, phenyl C_3_-H, C_5_-H), 11.07 (s, 1H, CS-NH), 11.47 (s, 1H, N-NH), 12.78 (s, 1H, indole NH). MS, *m/z* (rel. int.): 448 (M^+^, 24), 421 (24), 420 (100), 414 (18), 388 (26), 372 (2), 351 (10), 331 (3), 287 (3), 264 (7), 243 (31), 230 (71), 215 (31), 202 (70), 188 (27), 187 (50), 186 (84), 167 (36), 161 (25), 160 (15), 159 (39), 145 (50), 133 (80), 120 (7), 105 (22), 95 (13), 78 (15), 69 (43), 44 (6). Anal calcd. for C_17_H_10_F_6_N_4_O_2_S: C, 45.54, H, 2.25, N, 12.50; found: C, 45.53, H, 2.23, N, 12.49.

*2-[2-Oxo-5-(trifluoromethoxy)-1,2-dihydro-3H-indol-3-ylidene]-N-[4-(trifluoromethoxy)phenyl]-1-hydrazinecarbothioamide* (**3l**). Yield 72% as orange fluffy crystals, mp: 228–230 °C. IR: 3308, 3100, 3055 (NH stretching), 1699 (C=O), 1600 (C=N), 1159 (C=S) cm^−1^. ^1^H-NMR (DMSO-*d_6_*): *δ* 7.04 (d, *J* = 8.4 Hz, 1H, indole C_7_-H), 7.38 (dd, *J* = 8.4, 1.8 Hz, 1H, indole C_6_-H), 7.45 (d, *J* = 8.4 Hz, 2H, phenyl C_2_-H, C_6_-H), 7.71–7.77 (m, 3H, phenyl C_3_-H, C_5_-H, indole C_4_-H), 7.77 (d, *J* = 6.3 Hz, 1H, indole C_4_-H), 10.97 (s, 1H, CS-NH), 11.44 (s, 1H, indole NH), 12.71 (s, 1H, N-NH). MS, *m/z* (rel. int.): 464 (M^+^, 12), 436 (88), 430 (28), 404 (21), 388 (2), 367 (10), 345 (4), 287 (5), 245 (18), 243 (55), 230 (56), 228 (60), 220 (13), 219 (23), 215 (46), 203 (29), 202 (100), 188 (27), 187 (44), 177 (18), 160 (12), 159 (24), 134 (15), 133 (100), 131 (11), 108 (13), 105 (32), 95 (10), 86 (7), 78 (9), 69 (53), 63 (10), 44 (33). Anal calcd. for C_17_H_10_F_6_N_4_O_3_S: C, 43.97, H, 2.17, N, 12.07; found: C, 44.10, H, 2.17, N, 12.09.

*N-(2,4-Dimethoxyphenyl)-2-[2-oxo-5-(trifluoromethoxy)-1,2-dihydro-3H-indol-3-ylidene]-1-hydrazinecarbothioamide* (**3m**). Yield 89% as orange fluffy crystals, mp: 248 °C. IR: 3269, 3200, 3100 (NH stretching), 1705 (C=O), 1608 (C=N), 1165 (C=S) cm^−1^. ^1^H-NMR (DMSO-*d_6_*): *δ* 3.80 (s, 6H, OCH_3_), 6.58 (dd, *J* = 8.7, 2.4 Hz, 1H, phenyl C_5_-H), 6.69 (d, *J* = 2.4 Hz, 1H, phenyl C_3_-H), 7.03 (d, *J* = 8.7 Hz, 1H, indole C_7_-H), 7.30–7.38 (m, 2H, indole C_6_-H, phenyl C_6_-H), 7.76 (s, 1H, indole C_4_-H), 10.45 (s, 1H, CS-NH), 11.39 (s, 1H, indole NH), 12.58 (s, 1H, N-NH). MS, *m/z* (rel. int.): 440 (M^+^, 20), 426 (5), 412 (44), 381 (3), 343 (4), 287 (8), 245 (76), 228 (33), 217 (11), 195 (100), 188 (40), 180 (30), 152 (41), 138 (27), 120 (11), 95 (11), 69 (27). Anal calcd. for C_18_H_15_F_3_N_4_O_4_S: C, 49.09, H, 3.43, N, 12.72; found: C, 49.13, H, 3.43, N, 12.74.

*N-(2,4-Difluorophenyl)-2-[2-oxo-5-(trifluoromethoxy)-1,2-dihydro-3H-indol-3-ylidene]-1-hydrazinecarbothioamide* (**3n**). Yield 81% as yellow fluffy crystals, mp: 228–230 °C. IR: 3316, 3206, 3084 (NH stretching), 1692 (C=O), 1611 (C=N), 1155 (C=S) cm^-1^. ^1^H-NMR (DMSO-*d_6_*): *δ* 7.04 (d, *J* = 8.7 Hz, 1H, indole C_7_-H), 7.19 (td, *J* = 8.4,1.2 Hz, 1H, phenyl C_5_-H), 7.37–7.58 (m, 3H, indole C_6_-H, phenyl C_3_-H, C_6_-H), 7.71 (s, 1H, indole C_4_-H), 10.77 (s, 1H, CS-NH), 11.44 (s, 1H, indole NH), 12.73 (s, 1H, N-NH). MS, *m/z* (rel. int.): 416 (M^+^, 18), 389 (20), 388 (100), 382 (36), 356 (12), 337 (2), 319 (13), 297 (2), 287 (6), 263 (6), 243 (45), 230 (38), 229 (13), 228 (41), 215 (33), 202 (33), 188 (24), 187 (28), 172 (11), 159 (18), 155 (12), 154 (85), 133 (32), 129 (20), 101(16), 82 (4), 69 (24), 44 (15). Anal. calcd. for C_16_H_9_F_5_N_4_O_2_S: C, 46.16, H, 2.18, N, 13.46; found: C, 46.11, H, 2.17, N, 13.43.

*N-(2,6-Difluorophenyl)-2-[2-oxo-5-(trifluoromethoxy)-1,2-dihydro-3H-indol-3-ylidene]-1-hydrazinecarbothioamide* (**3o**). Yield 70% as yellow fluffy crystals, mp: 248–250 °C. IR: 3333, 3256, 3100 (NH stretching), 1694 (C=O), 1610 (C=N), 1165 (C=S) cm^−1^. ^1^H-NMR (DMSO-*d_6_*): *δ* 7.04 (d, *J* = 8.4 Hz, 1H, indole C_7_-H), 7.28 (t, *J* = 8.1 Hz, 2H, phenyl C_3_-H, C_5_-H), 7.39 (dd, *J* = 8.4, 1.8 Hz, 1H, indole C_6_-H), 7.48–7.57 (m, 1H, phenyl C_4_-H), 7.69 (s, 1H, indole C_4_-H), 10.66 (s, 1H, CS-NH), 11.44 (s, 1H, indole NH), 12.81 (s, 1H, N-NH). MS, *m/z* (rel. int.): 416 (M^+^, 13), 389 (20), 388 (99), 382 (44), 356 (7), 337 (3), 319 (10), 287 (9), 267 (7), 243 (48), 230 (39), 229 (15), 228 (44), 216 (14), 215 (39), 202 (36), 188 (24), 187 (32), 172 (11), 159 (21), 154 (100), 134 (15), 133 (38), 127 (23), 101 (13), 85 (11), 83 (16), 69 (31), 44 (4). Anal calcd. for C_16_H_9_F_5_N_4_O_2_S: C, 46.16, H, 2.18, N, 13.46; found: C, 46.17, H, 2.19, N, 13.45.

*N-(3,5-Difluorophenyl)-2-[2-oxo-5-(trifluoromethoxy)-1,2-dihydro-3H-indol-3-ylidene]-1-hydrazinecarbothioamide* (**3p**). Yield 70% as yellow powder, mp: 236–238 °C. IR: 3308, 3262, 3102 (NH stretching), 1703 (C=O), 1600 (C=N), 1155 (C=S) cm^−1^. ^1^H-NMR (DMSO-*d_6_*): δ 7.03 (d, *J* = 8.7 Hz, 1H, indole C_7_-H), 7.19 (tt, *J* = 9.3, 2.4 Hz, 1H, phenyl C_4_-H), 7.39 (dd, *J* = 8.7, 1.8 Hz, 1H, indole C_6_-H), 7.56 (dd, *J* = 9.0, 2.1 Hz, 2H, phenyl C_2_-H, C_6_-H), 7.78 (d, *J* = 1.5 Hz, 1H, indole C_4_-H), 10.95 (s, 1H, CS-NH), 11.47 (s, 1H, indole NH), 12.79 (s, 1H, N-NH). MS, *m/z* (rel. int.): 416 (M^+^, 7), 388 (38), 382 (19), 356 (14), 319 (5), 287 (12), 287 (12), 267 (6), 245 (14), 243 (38), 230 (58), 228 (60), 215 (32), 202 (53), 188 (24), 187 (32), 171 (14), 159 (30), 155 (18), 154 (100), 133 (60), 127 (65), 113 (20), 101 (16), 83 (37), 69 (28), 44 (11). Anal calcd. for C_16_H_9_F_5_N_4_O_2_S: C, 46.16, H, 2.18, N, 13.46, found: C, 46.14, H, 2.18, N, 13.42.

*N-(4-Bromo-2-fluorophenyl)-2-[2-oxo-5-(trifluoromethoxy)-1,2-dihydro-3H-indol-3-ylidene]-1-hydrazinecarbothioamide* (**3q**). Yield 74% as yellow powder, mp: 238–240 °C. IR: 3300, 3173, 3129 (NH stretching), 1701 (C=O), 1580 (C=N), 1144 (C=S) cm^−1^. ^1^H-NMR (DMSO-*d_6_*): δ 7.04 (d, *J* = 8.4 Hz, 1H, indole C_7_-H), 7.39 (d, *J* = 8.7 Hz, 1H, indole C_6_-H), 7.45–7.54 (m, 2H, phenyl C_5_-H, C_6_-H), 7.70–7.76 (m, 2H, indole C_4_-H, phenyl C_3_-H ), 10.79 (s, 1H, CS-NH), 11.44 (s, 1H, indole NH), 12.76 (s, 1H, N-NH). MS, *m/z* (rel. int.): 478/476 (M^+^, 7/8), 450/448 (55/51), 444/442 (16/15), 418/416 (9/9), 381/379 (5/5), 363 (3), 287 (9), 245/243 (19/47), 243 (47), 233/231 (18/24), 230/228 (60/85), 217/215 (28/58), 216/214 (100/100), 203/201 (7/20), 191 (18) 189/187 (28/30), 159 (33), 135/133 (18/60), 131 (16), 115 (15), 108 (43), 83 (15), 69 (33), 44 (10). Anal calcd. for C_16_H_9_BrF_4_N_4_O_2_S: C, 40.27, H, 1.90, N, 11.74, found: C, 40.26, H, 1.90, N, 11.70.

*N-(2,4-Dichlorophenyl)-2-[2-oxo-5-(trifluoromethoxy)-1,2-dihydro-3H-indol-3-ylidene]-1-hydrazinecarbothioamide* (**3r**). Yield 50% as shinning yellow powder, mp: 253–255 °C. IR: 3258, 3184, 3080 (NH stretching), 1701 (C=O), 1580 (C=N), 1155 (C=S) cm^−1^. ^1^H-NMR (DMSO-*d_6_*): δ 7.04 (d, *J* = 8.1 Hz, 1H, indole C_7_-H), 7.39 (d, *J* = 7.5 Hz, 1H phenyl C_6_-H), 7.55 (s, 2H, phenyl C_5_-H, C_6_-H), 7.72 (s, 1H, phenyl C_3_-H), 7.82 (s, 1H, indole C_4_-H), 10.91 (s, 1H, CS-NH), 11.44 (s, 1H, indole NH), 12.73 (s, 1H, N-NH). MS, *m/z* (rel. int.): 422 (7), 420 (10), 417 (5), 416 (17), 415 (64), 414 (30), 413 (100), 287 (7), 228 (6), 186 (13), 161 (13), 78 (5). Anal calcd. for C_16_H_9_Cl_2_F_3_N_4_O_2_S: C, 42.78, H, 2.02, N, 12.47; found: C, 42.75, H, 2.01, N, 12.44.

*N-(3,4-Dichlorophenyl)-2-[2-oxo-5-(trifluoromethoxy)-1,2-dihydro-3H-indol-3-ylidene]-1-hydrazinecarbothioamide* (**3s**). Yield 82% as yellow powder, mp: 245 °C. IR: 3318, 3177, 3080 (NH stretching), 1697 (C=O), 1600 (C=N), 1153 (C=S) cm^−1^. ^1^H-NMR (DMSO-*d_6_*): δ 7.04 (d, *J* = 8.4 Hz, 1H, indole C_7_-H), 7.39 (d, *J* = 8.1 Hz, 1H, indole C_6_-H), 7.71 (s, 2H, phenyl C_5_-H, C_6_-H) 7.77 (s, 1H, phenyl C_2_-H), 8.01 (s, 1H, indole C_4_-H), 10.97 (s, 1H, CS-NH), 11.46 (s, 1H, indole NH), 12.76 (s, 1H, N-NH). MS, *m/z* (rel. int.): 450/448 (M^+^, 16/ 27), 422 (87), 420 (100), 351 (11), 287 (78), 263 (9), 245 (53), 230/228 (11/18), 229 (43), 206/204 (7/12), 201 (52), 188 (10), 161 (98), 133 (12), 120 (10), 99 (13), 77 (6). Anal calcd. for C_16_H_9_Cl_2_F_3_N_4_O_2_S: C, 42.78, H, 2.02, N, 12.47; found: C, 42.79, H, 2.02, N, 12.49.

*N-(5-Chloro-2-methylphenyl)-2-[2-oxo-5-(trifluoromethoxy)-1,2-dihydro-3H-indol-3-ylidene]-1-hydrazinecarbothioamide* (**3t**). Yield 64% as orange powder, mp: 240 °C. IR: 3302, 3233, 3082 (NH stretching), 1701 (C=O), 1584 (C=N), 1146 (C=S) cm^−1^. ^1^H-NMR (DMSO-*d_6_*): δ 2.23 (s, 3H, CH_3_), 7.03 (d, *J* = 8.7 Hz, 1H, indole C_7_-H), 7.37–7.39 (m, 4H, indole C_6_-H, phenyl C_3_-H, C_4_-H, C_6_-H), 7.73 (s, 1H, indole C_4_-H), 10.84 (s, 1H, CS-NH), 11.42 (s, 1H, indole NH), 12.66 (s, 1H, N-NH). MS, *m/z* (rel. int.): 430/428 (M^+^, 9/22), 402 (47), 400 (100), 333/331 (4/9), 287 (4), 263 (6), 246 (6), 245 (33), 234 (7), 216 (12), 188 (20), 183 (15), 166 (7), 148 (21), 120 (7), 106 (7), 89 (8), 77 (7), 69 (18). Anal calcd. for C_17_H_12_ClF_3_N_4_O_2_S: C, 47.62, H, 2.82, N, 13.07; found: C, 47.60, H, 2.83, N, 13.03.

*2-[2-Oxo-5-(trifluoromethoxy)-1,2-dihydro-3H-indol-3-ylidene]-N-(2,4,6-trichlorophenyl)-1-hydrazinecarbothioamide* (**3u**). Yield 79% as yellow powder, mp: 240 °C. IR: 3354, 3204, 3069 (NH stretching), 1701 (C=O), 1564 (C=N), 1167 (C=S) cm^−1^. ^1^H-NMR (DMSO-*d_6_*): δ 7.04 (d, *J* = 8.1 Hz, 1H, indole C_7_-H), 7.39 (d, *J* = 7.2 Hz, 1H, indole C_6_-H), 7.69 (s, 2H, phenyl C_3_-H, C_5_-H), 7.88 (s, 1H, indole C_4_-H), 10.94 (s, 1H, CS-NH), 11.44 (s, 1H, indole NH), 12.81 (s, 1H, N-NH). MS, *m/z* (rel. int.): 456/454 (6/6), 451/449 (15/70), 448/446 (33/13), 447 (100), 432/430 (9/42), 402 (7), 385/383 (7/14), 287 (38), 262 (5), 245 (19), 239/237 (11/12), 199 (26), 197/195 (76/82), 69 (6). Anal calcd. for C_16_H_8_Cl_3_F_3_N_4_O_2_S: C, 39.73, H, 1.67, N, 11.58; found: C, 39.82, H, 1.66, N, 11.56.

### 3.3. Bioassay of Toxic Activity

Brine shrimp (*Artemia salina* Leach) eggs were hatched in a shallow rectangular plastic dish (22 × 32 cm) filled with artificial sea water, which was prepared with a commercial salt mixture (Instant Ocean, Aquarium System, Inc., Mentor, OH, USA) and double-distilled water. An unequal partition was made in the plastic dish with the help of a perforated device. Approximately 50 mg of eggs were sprinkled into the large compartment, which was darkened, while the smaller compartment was opened to ordinary light. After two days, nauplii were collected by a pipette from the lighted side. A sample of each test compound was prepared by dissolving 2 mg in 2 mL of methanol. From this stock solution, 100, 10 and 1 µL were transferred to 9 vials, three for each dilution, and one vial was kept as control having 2 mL of methanol. The solvent was allowed to evaporate overnight. After two days, when shrimp larvae were ready, 1 mL of sea water and 10 shrimps were added to each vial (30 shrimps/dilution) and the volume was adjusted with sea water to 5 mL per vial. After 24 h, the number of survivors was counted [35,36]. Data were analyzed by a Finney computer programme to determine the LD_50_ values [37].

## 4. Conclusions

We have demonstrated the potential of *N*^4^-aryl substituted 5-trifluoromethoxyisatin-3-thiosemicarbazones to show toxic activity. Based on the preliminary data given in the Table 1 and in terms of further development and structure-activity relationship (SAR) studies, simultaneous substitution of different substituents at position-5 of the isatin scaffold and on the phenyl ring attached to N^4^ of the thiosemicarbazone moiety certainly warrants further investigation. Work in this regard along with extended SAR studies will be reported in the near future.

## Supplementary Materials

Supplementary File 1
