# Supplementary figures and images for: Synthesis and Toxicity Evaluation of Some N4-Aryl Substituted 5-Trifluoromethoxyisatin-3-thiosemicarbazones
Source: Molecules. 2011 Jul 29;16(8):6408–21. doi: 10.3390/molecules16086408 (PMC6264754; doi:10.3390/molecules16086408)

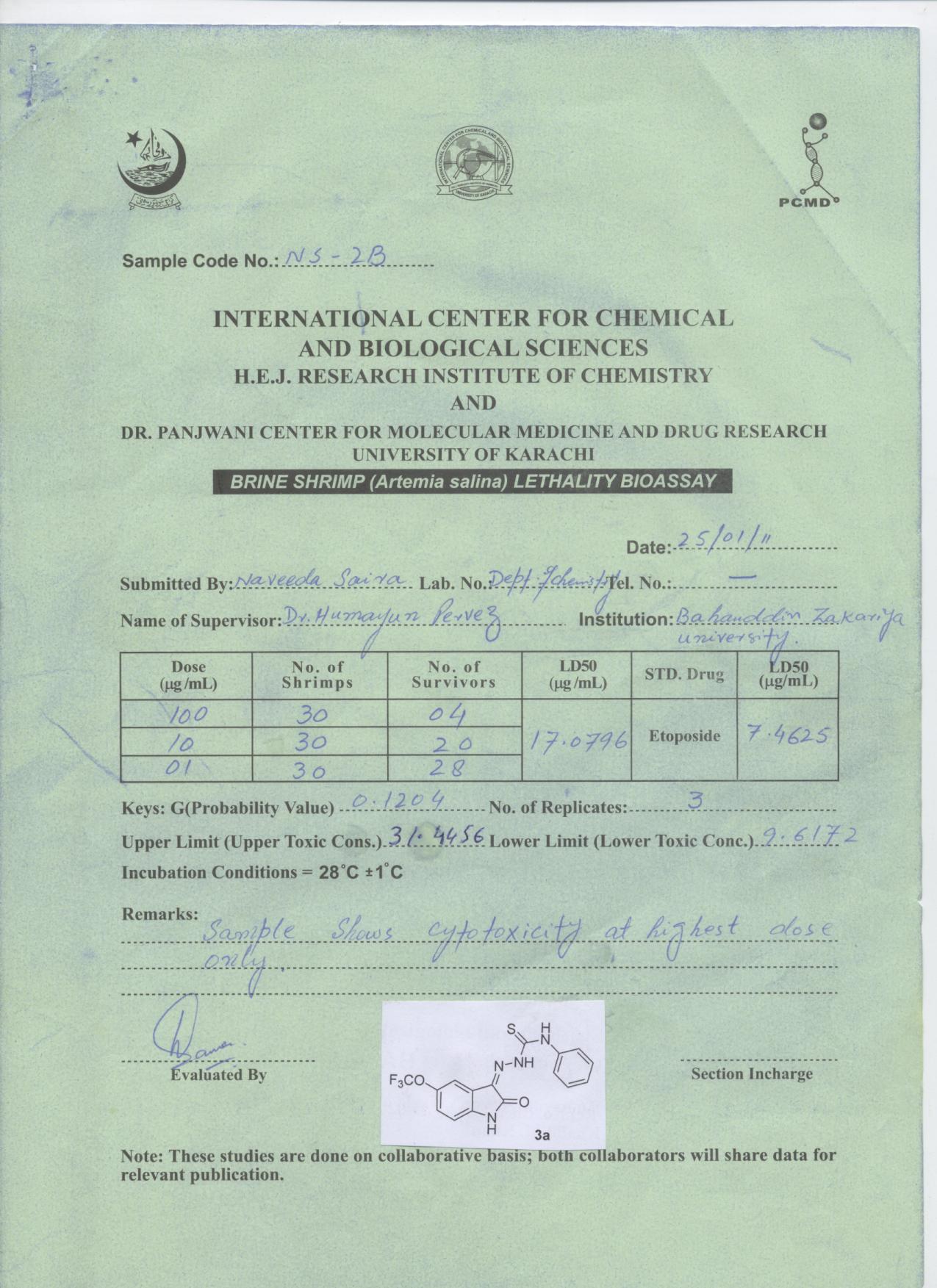

Supplement: Supplementary File 1 [file molecules-16-06408-s001.zip › Spectroscopy/Cytotoxity/3a.jpg]

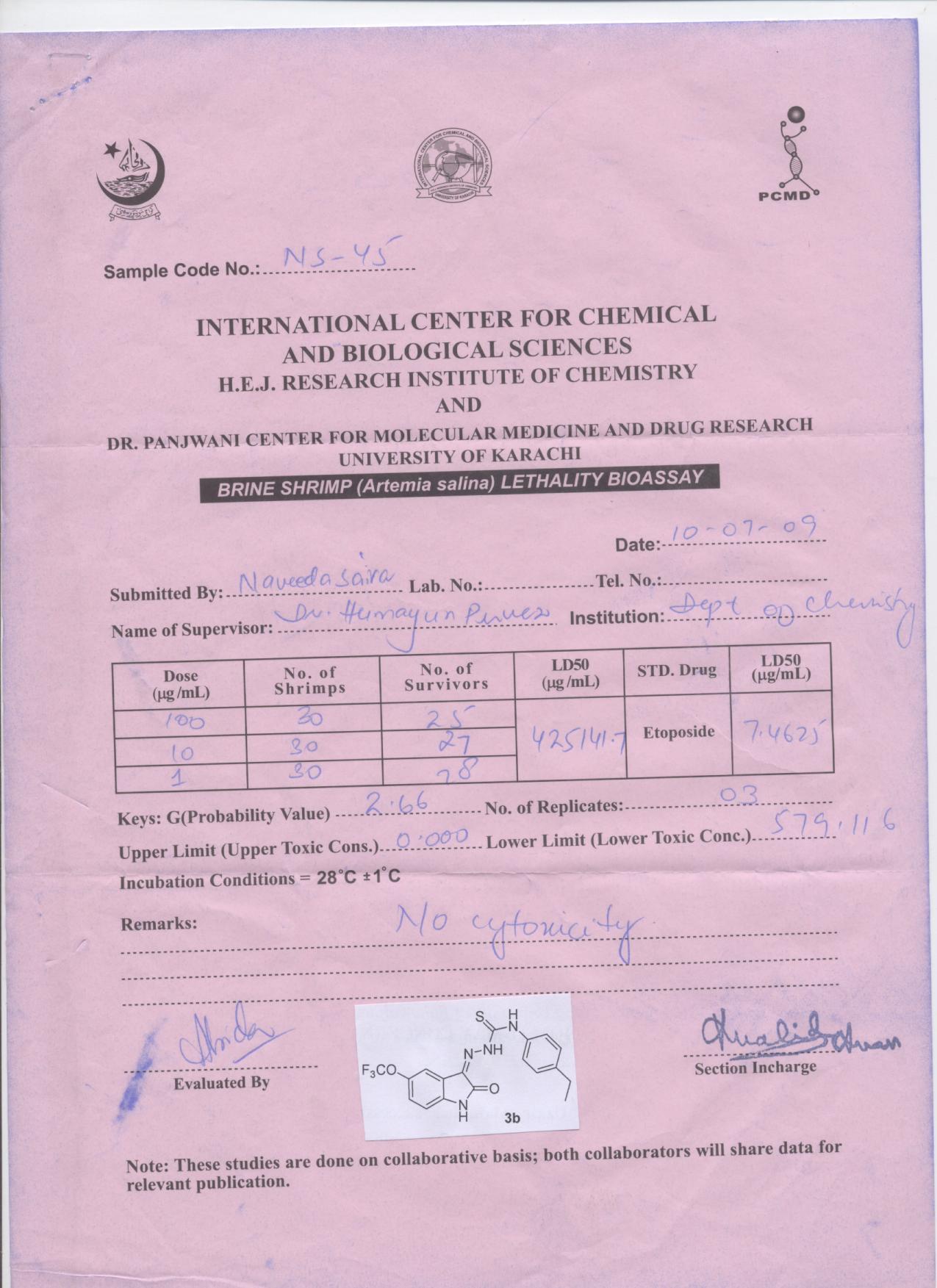

Supplement: Supplementary File 1 [file molecules-16-06408-s001.zip › Spectroscopy/Cytotoxity/3b.jpg]

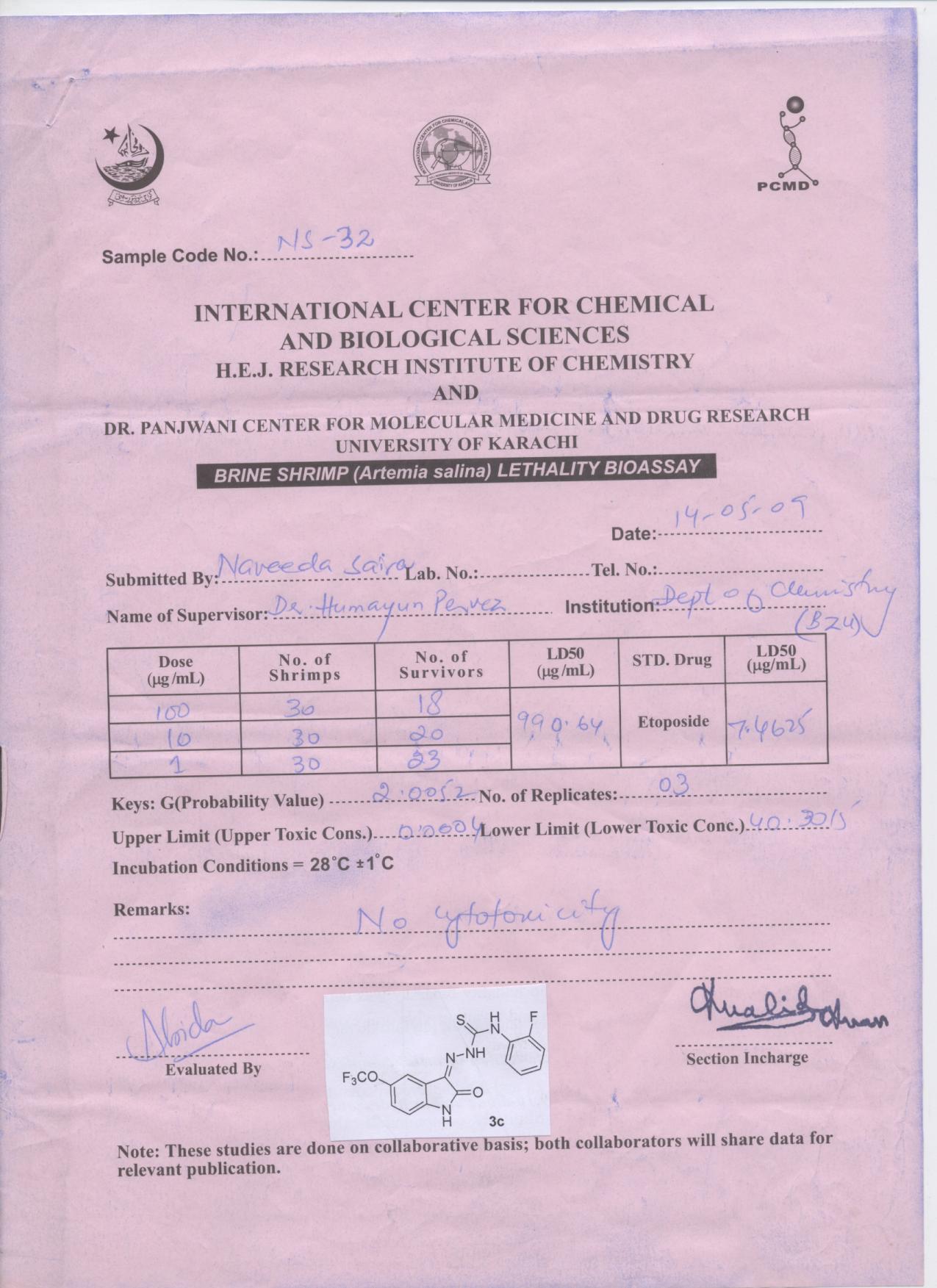

Supplement: Supplementary File 1 [file molecules-16-06408-s001.zip › Spectroscopy/Cytotoxity/3c.jpg]

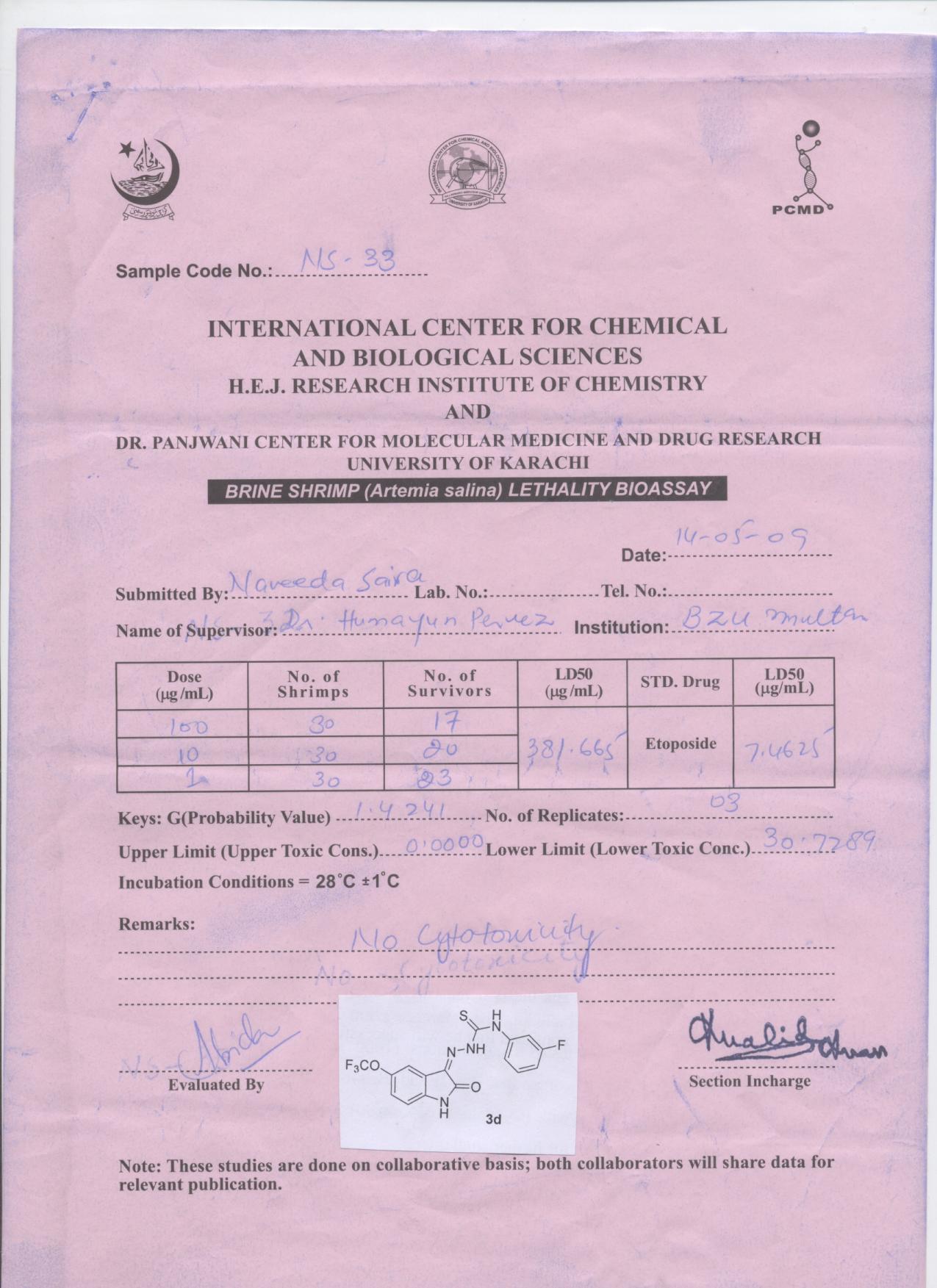

Supplement: Supplementary File 1 [file molecules-16-06408-s001.zip › Spectroscopy/Cytotoxity/3d.jpg]

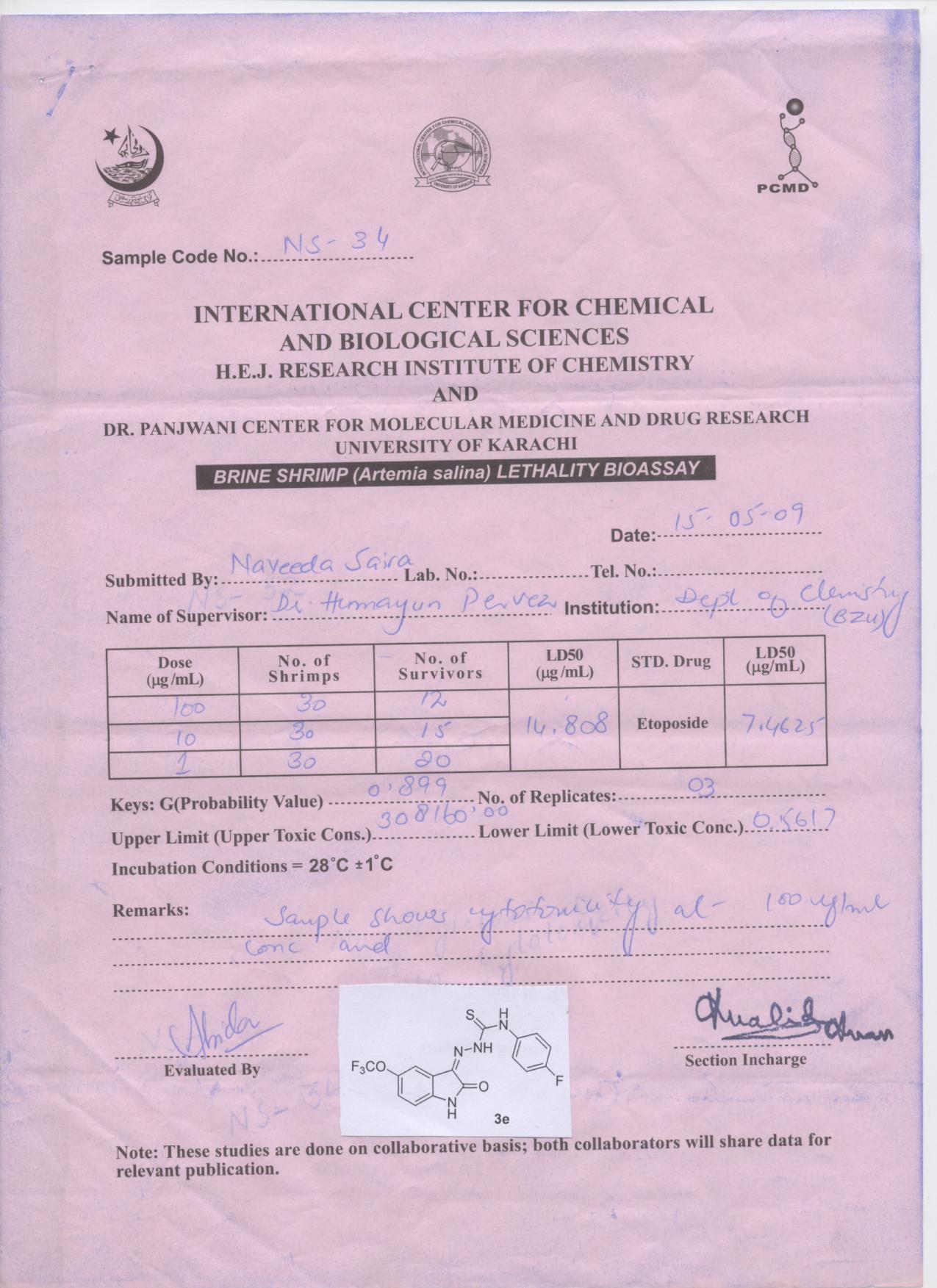

Supplement: Supplementary File 1 [file molecules-16-06408-s001.zip › Spectroscopy/Cytotoxity/3e.jpg]

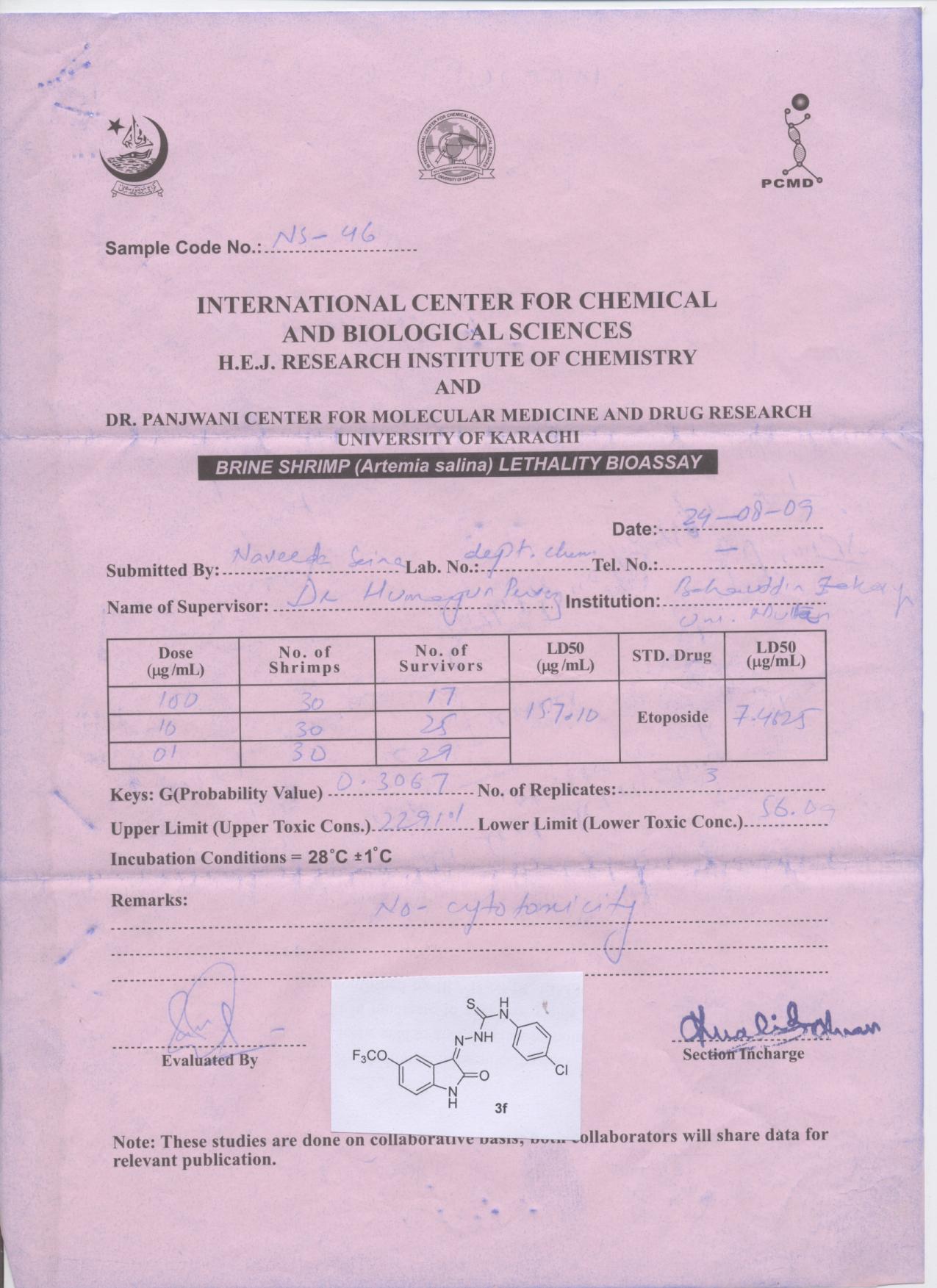

Supplement: Supplementary File 1 [file molecules-16-06408-s001.zip › Spectroscopy/Cytotoxity/3f.jpg]

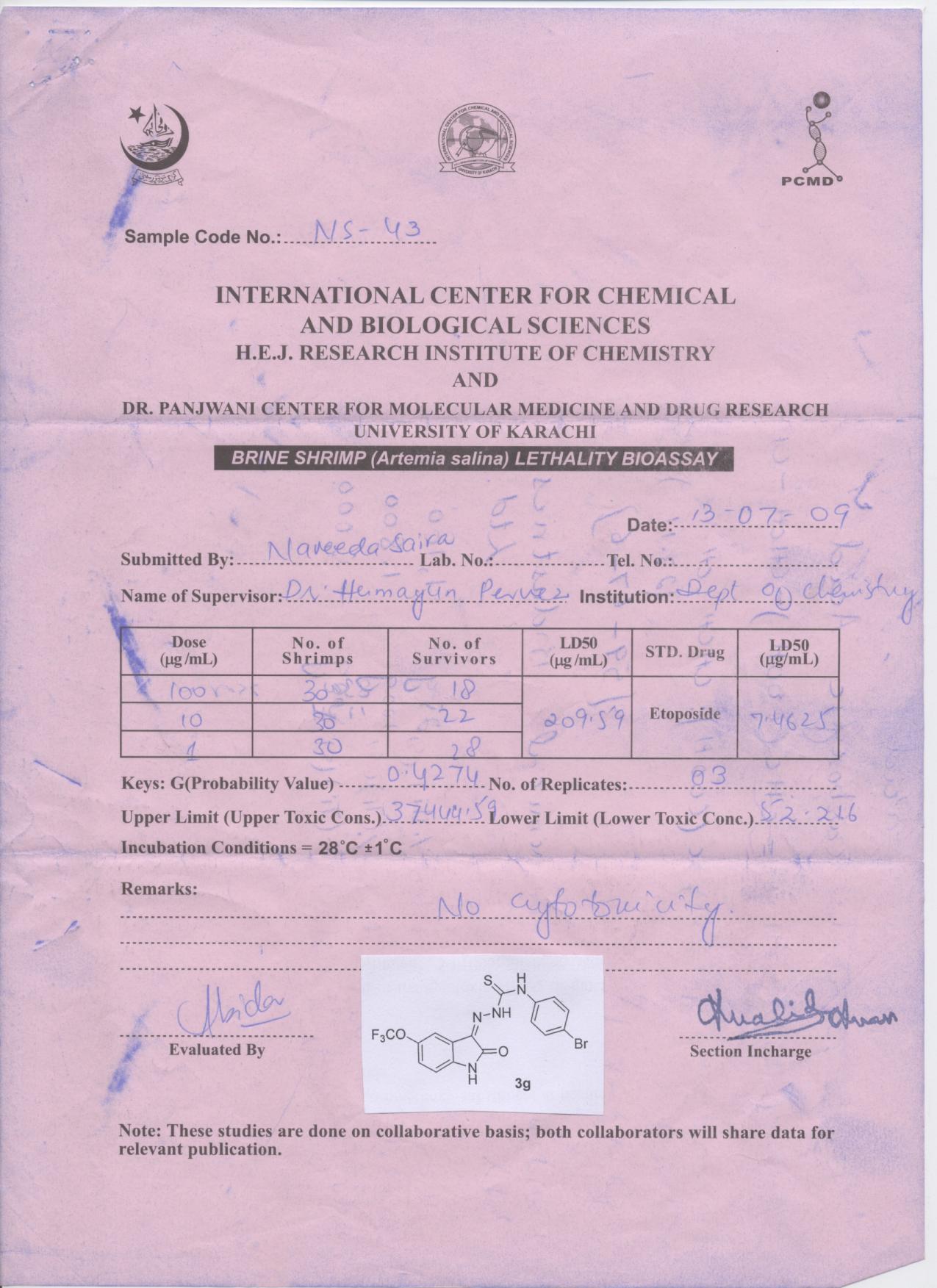

Supplement: Supplementary File 1 [file molecules-16-06408-s001.zip › Spectroscopy/Cytotoxity/3g.jpg]

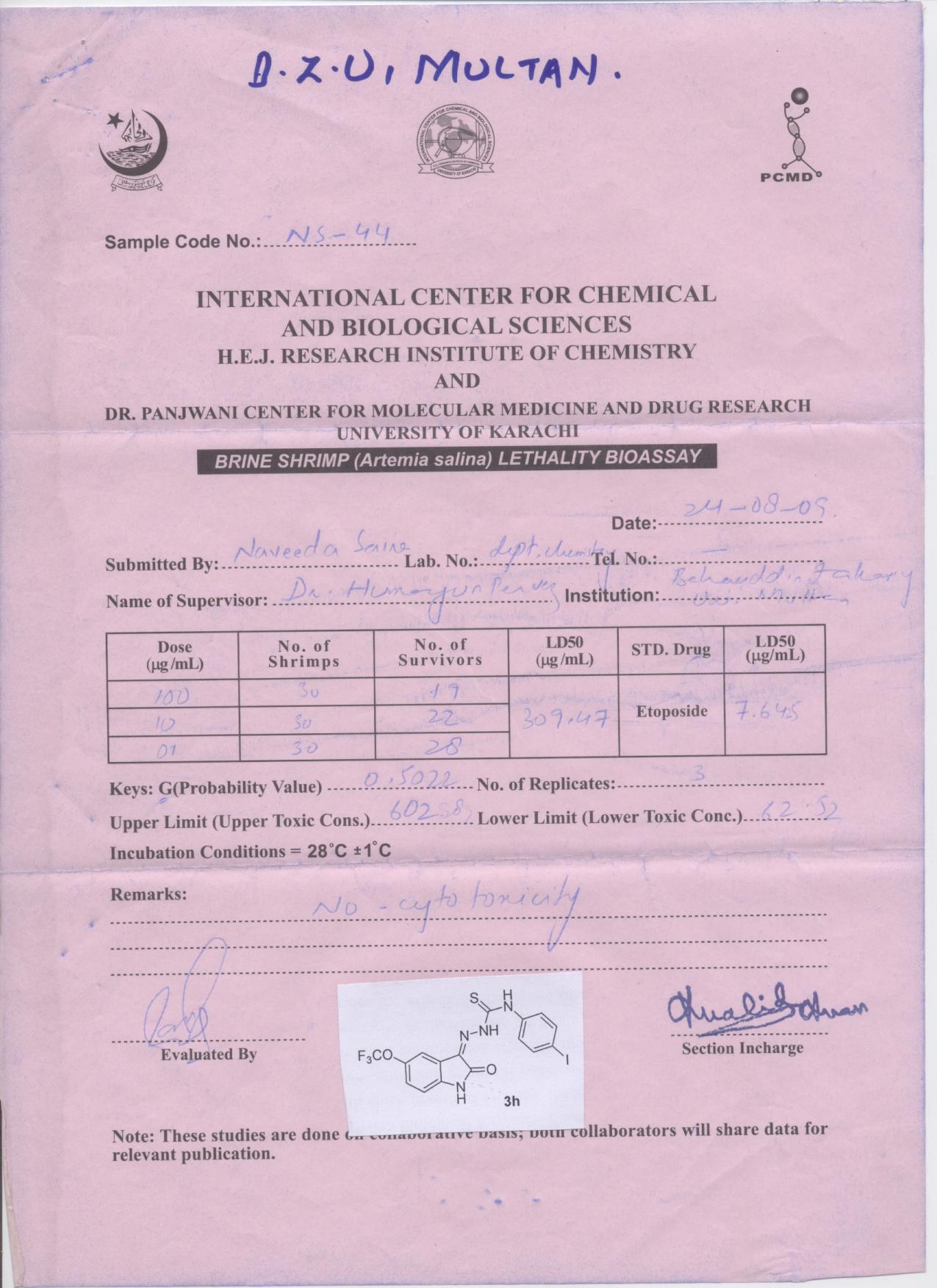

Supplement: Supplementary File 1 [file molecules-16-06408-s001.zip › Spectroscopy/Cytotoxity/3h.jpg]

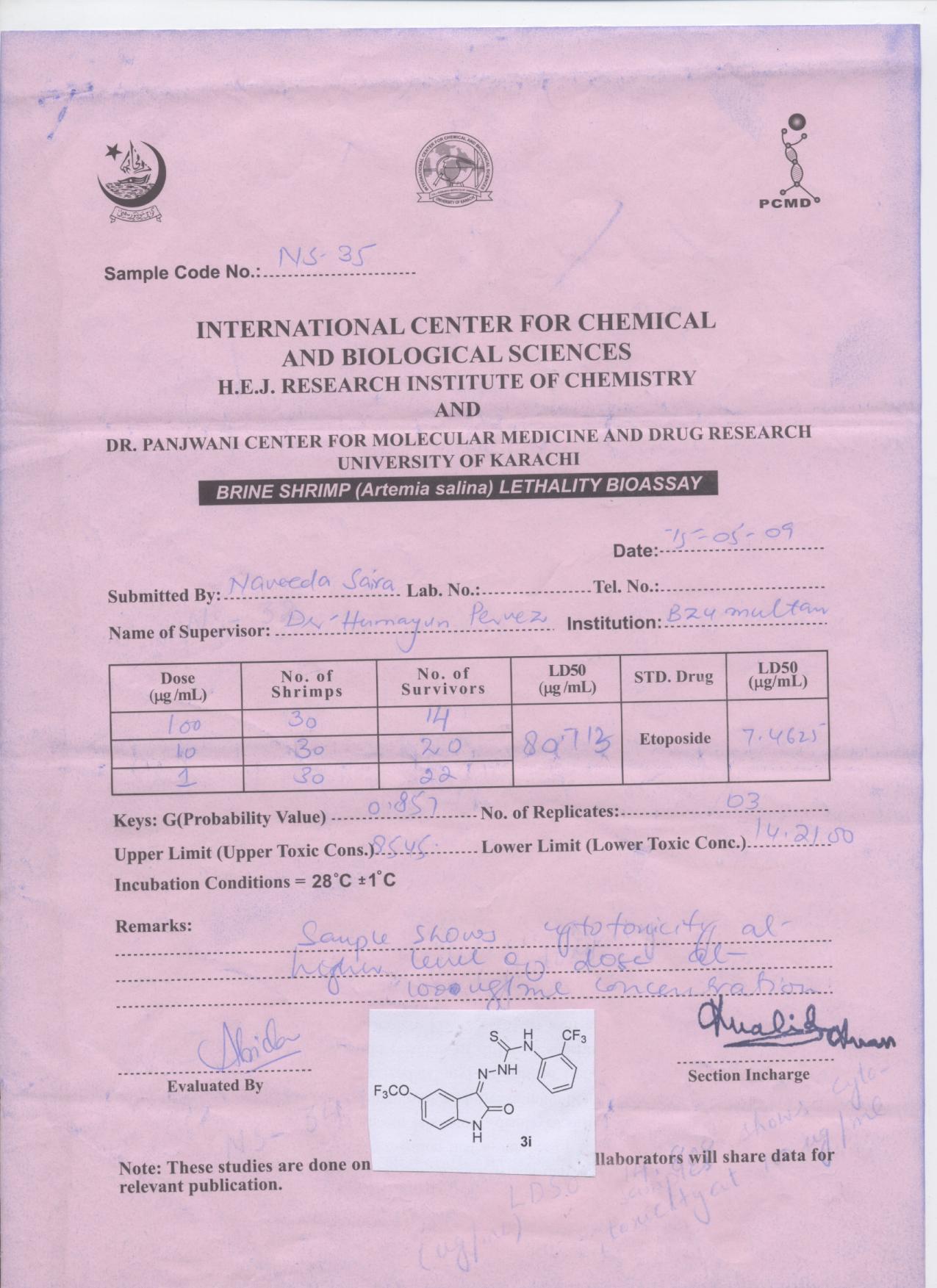

Supplement: Supplementary File 1 [file molecules-16-06408-s001.zip › Spectroscopy/Cytotoxity/3i.jpg]

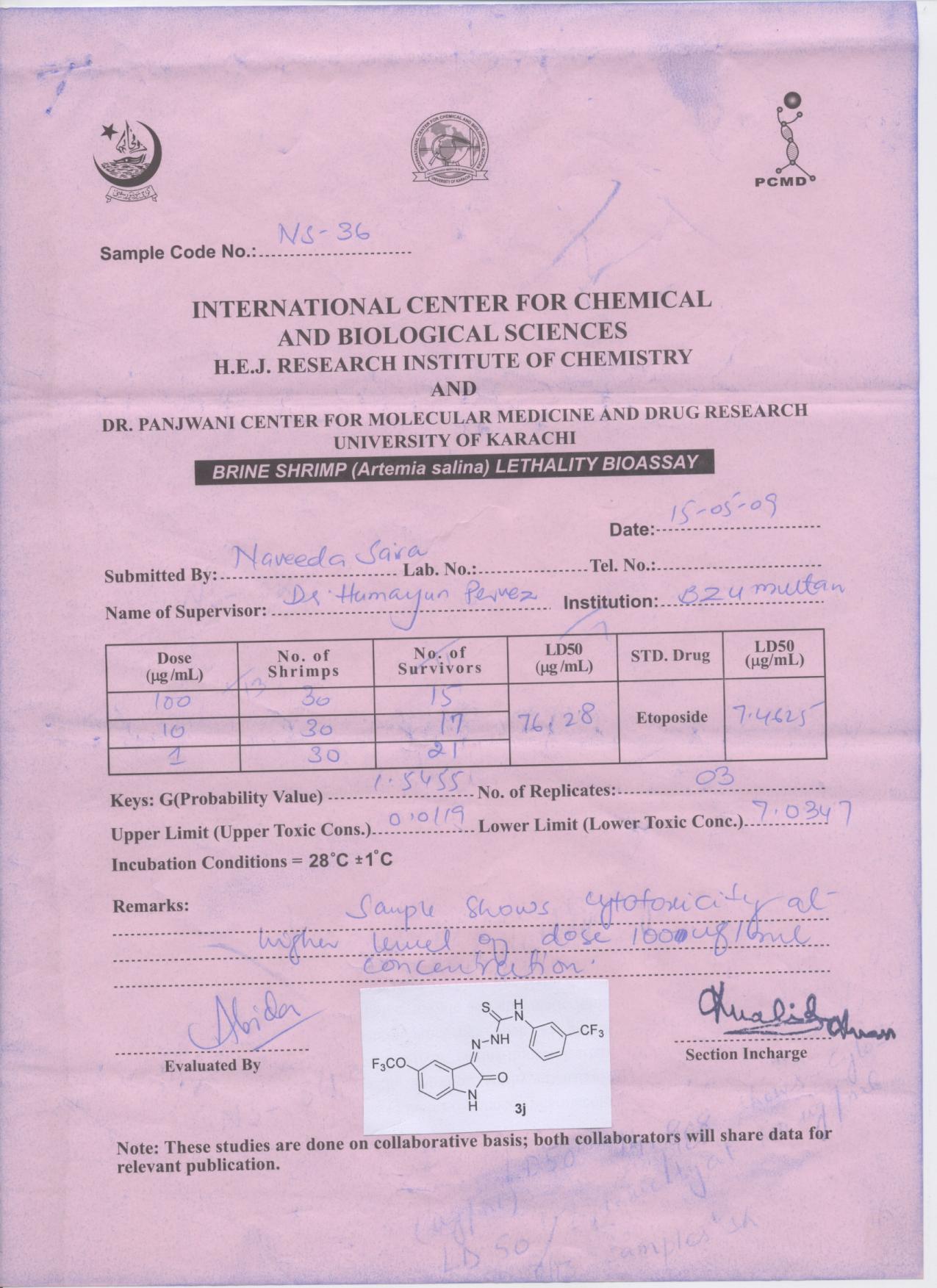

Supplement: Supplementary File 1 [file molecules-16-06408-s001.zip › Spectroscopy/Cytotoxity/3j.jpg]

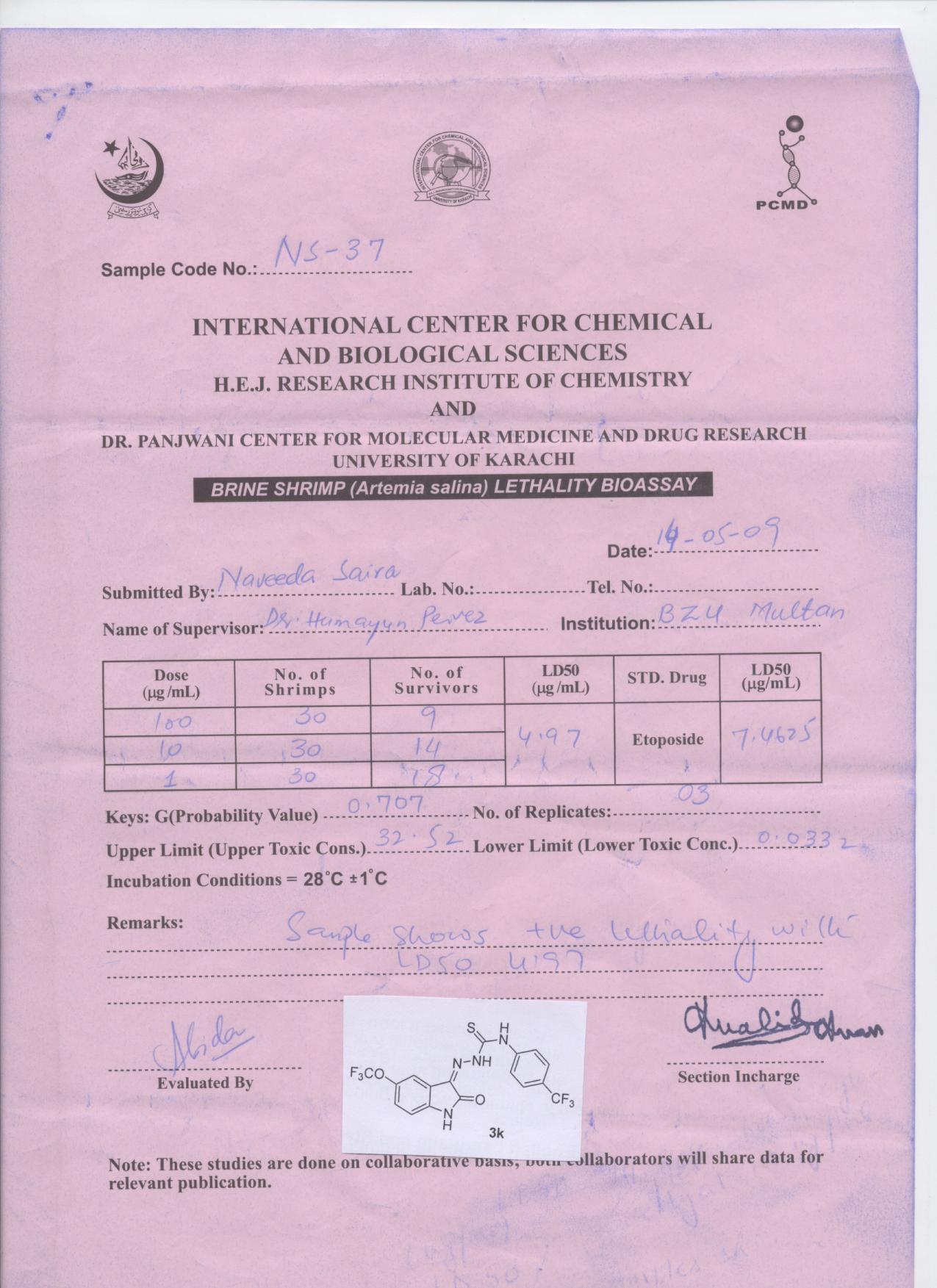

Supplement: Supplementary File 1 [file molecules-16-06408-s001.zip › Spectroscopy/Cytotoxity/3k.jpg]

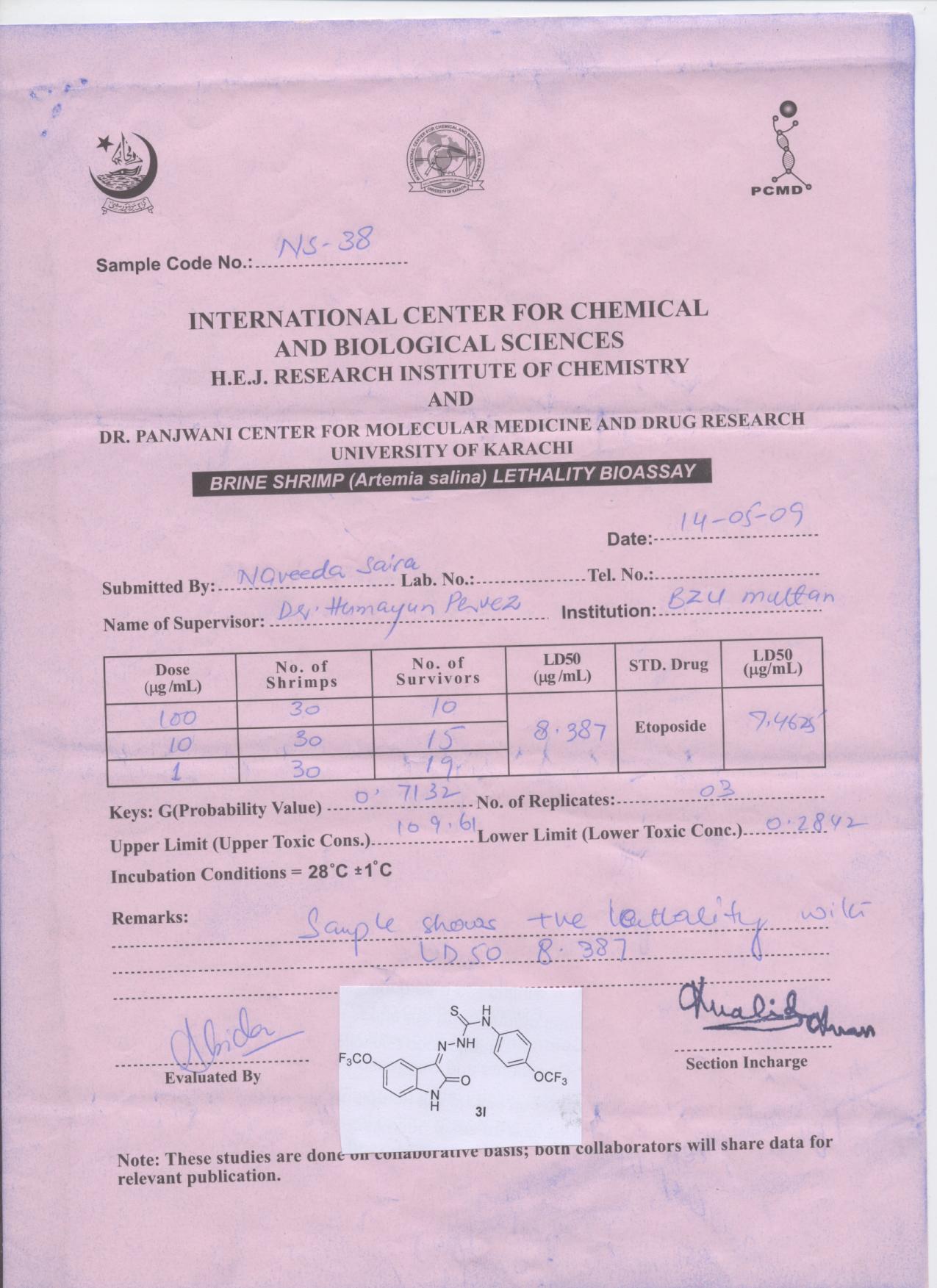

Supplement: Supplementary File 1 [file molecules-16-06408-s001.zip › Spectroscopy/Cytotoxity/3l.jpg]

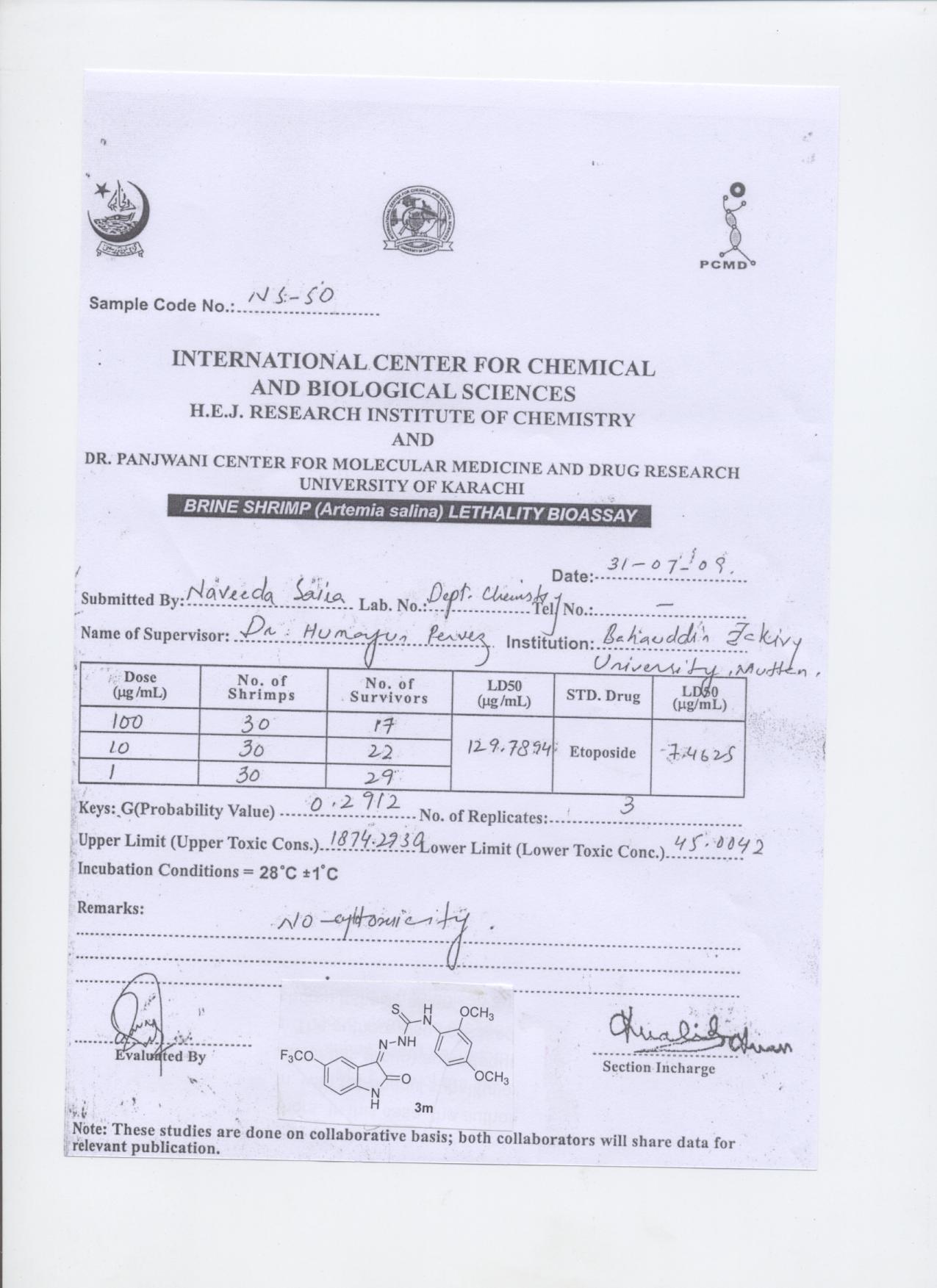

Supplement: Supplementary File 1 [file molecules-16-06408-s001.zip › Spectroscopy/Cytotoxity/3m.jpg]

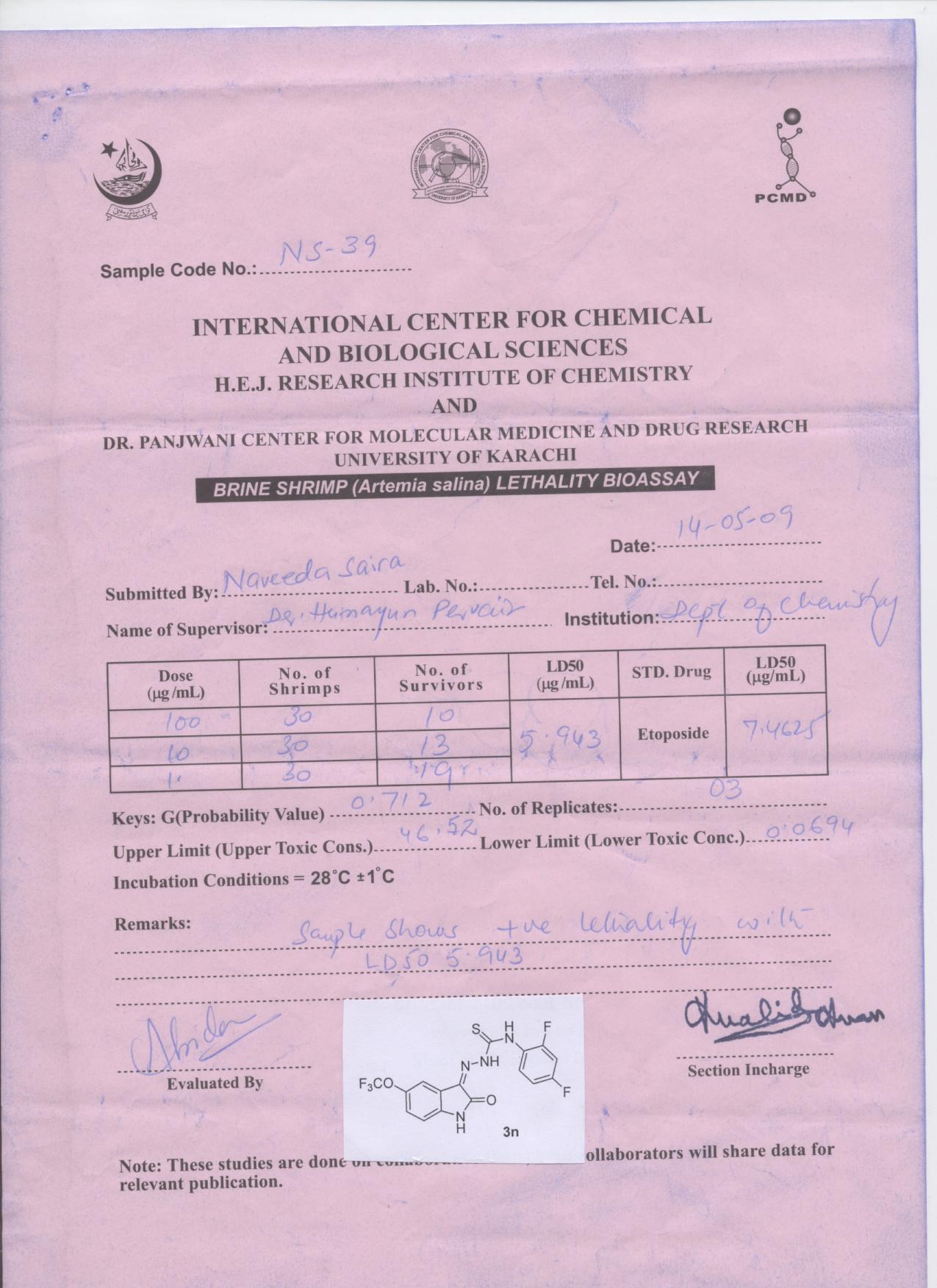

Supplement: Supplementary File 1 [file molecules-16-06408-s001.zip › Spectroscopy/Cytotoxity/3n.jpg]

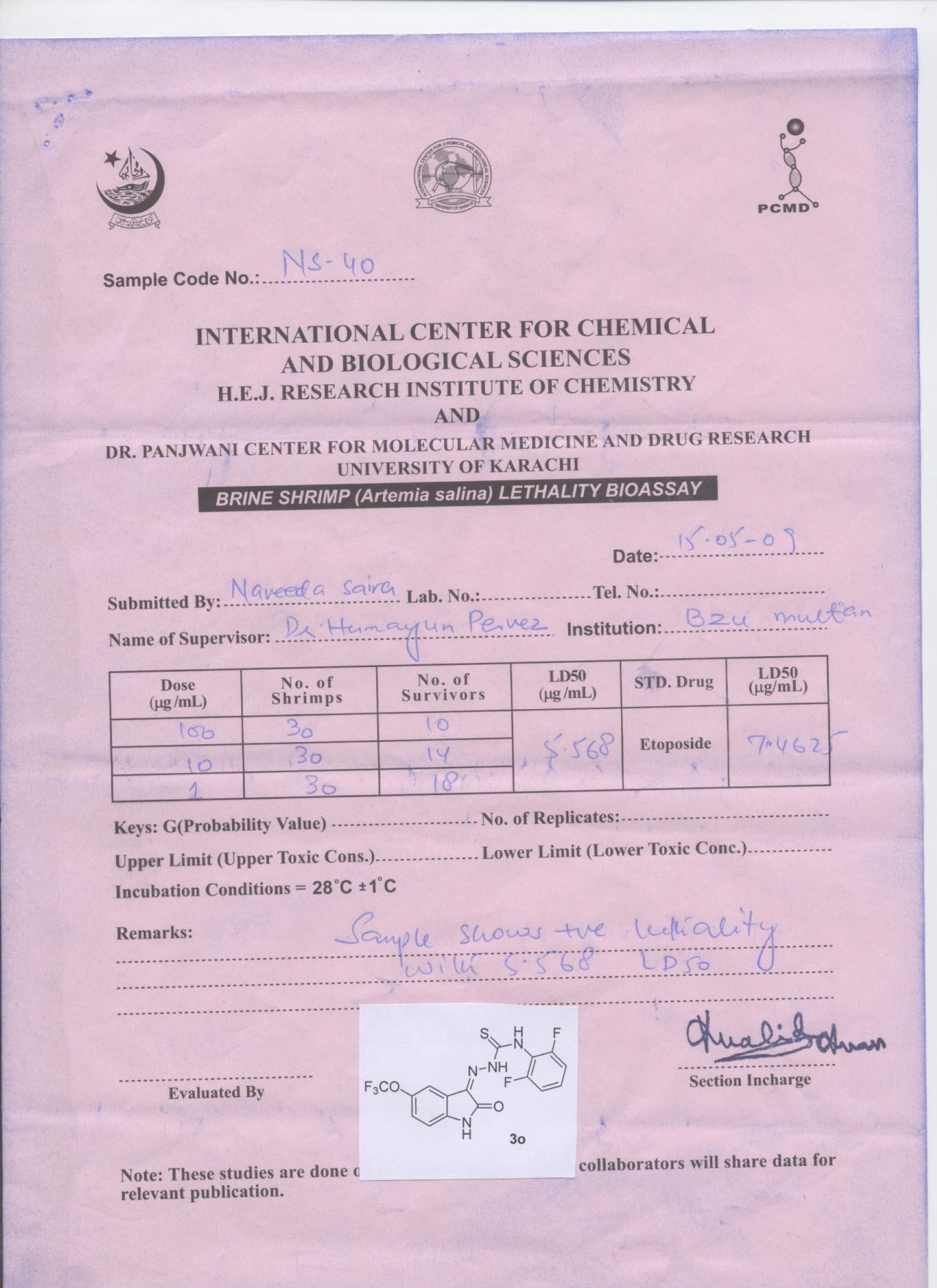

Supplement: Supplementary File 1 [file molecules-16-06408-s001.zip › Spectroscopy/Cytotoxity/3o.jpg]

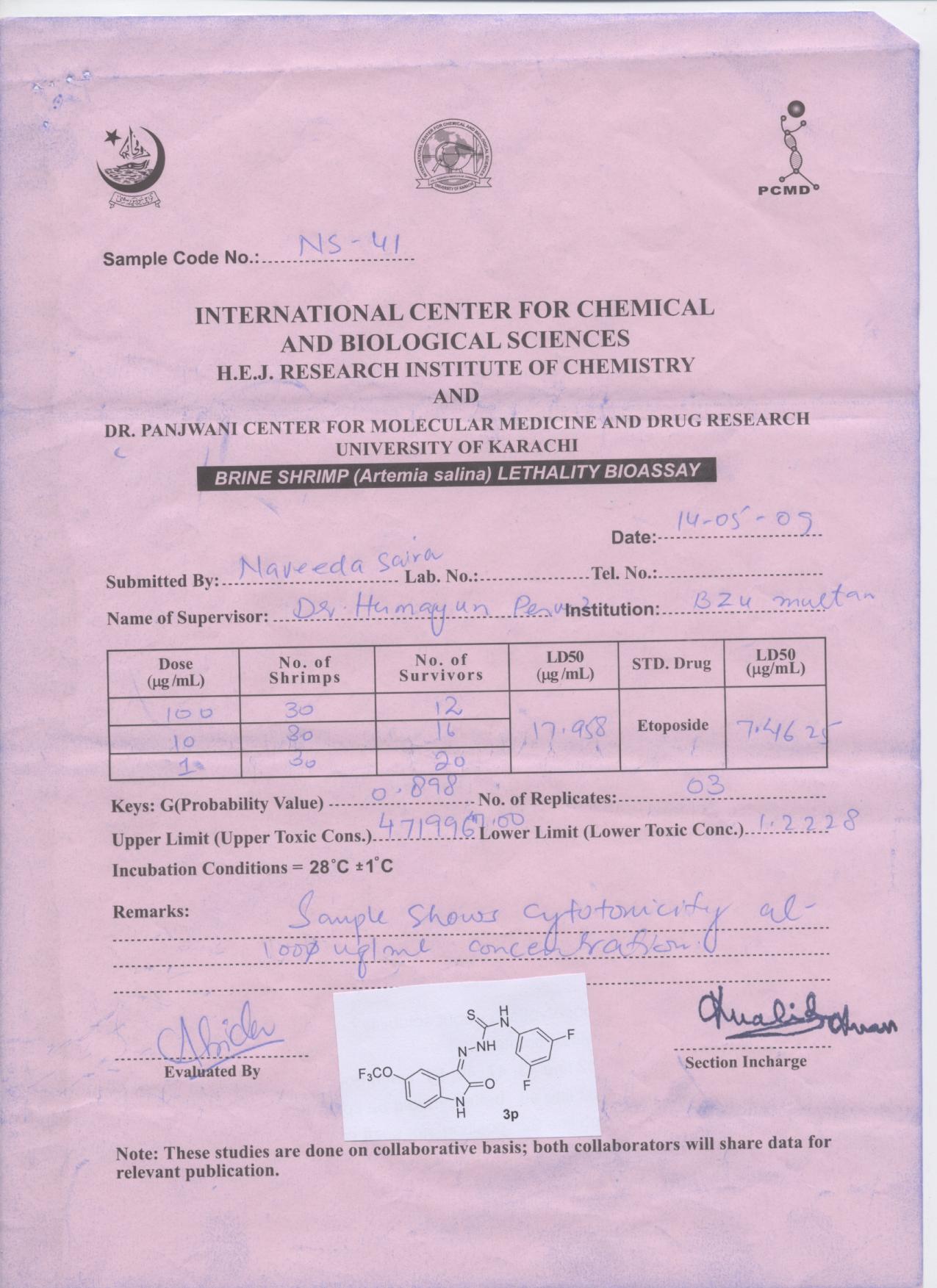

Supplement: Supplementary File 1 [file molecules-16-06408-s001.zip › Spectroscopy/Cytotoxity/3p.jpg]

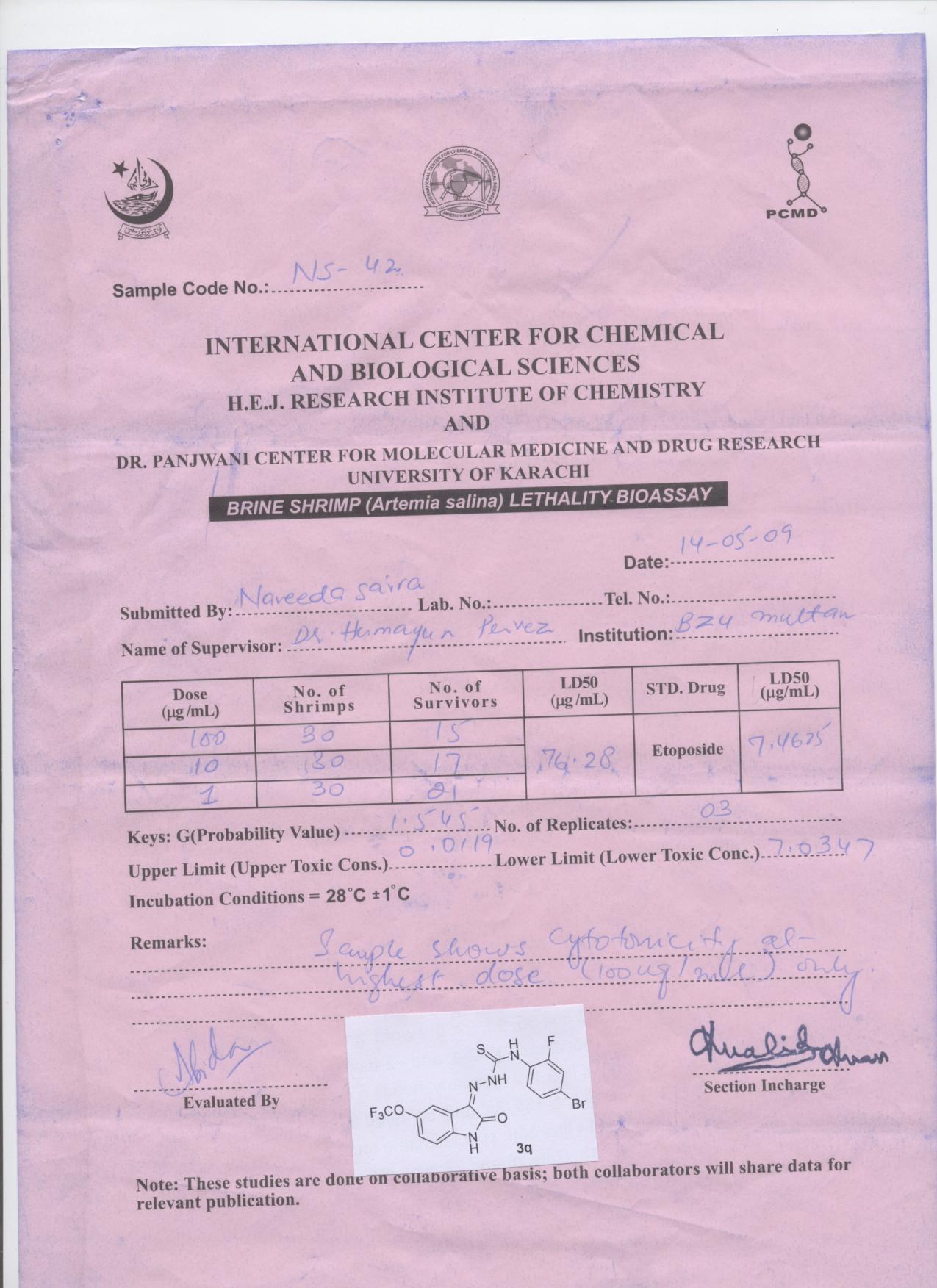

Supplement: Supplementary File 1 [file molecules-16-06408-s001.zip › Spectroscopy/Cytotoxity/3q.jpg]

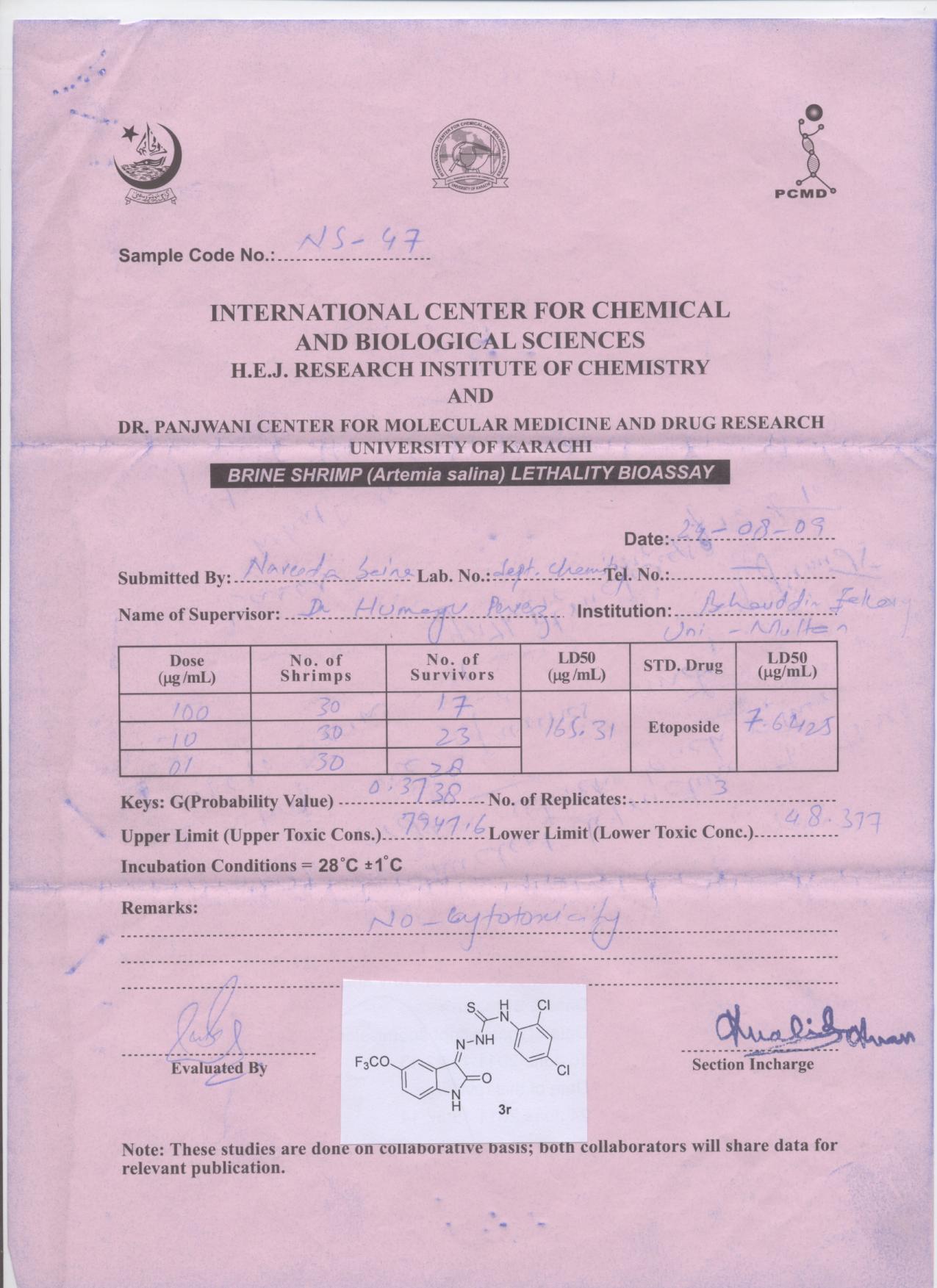

Supplement: Supplementary File 1 [file molecules-16-06408-s001.zip › Spectroscopy/Cytotoxity/3r.jpg]

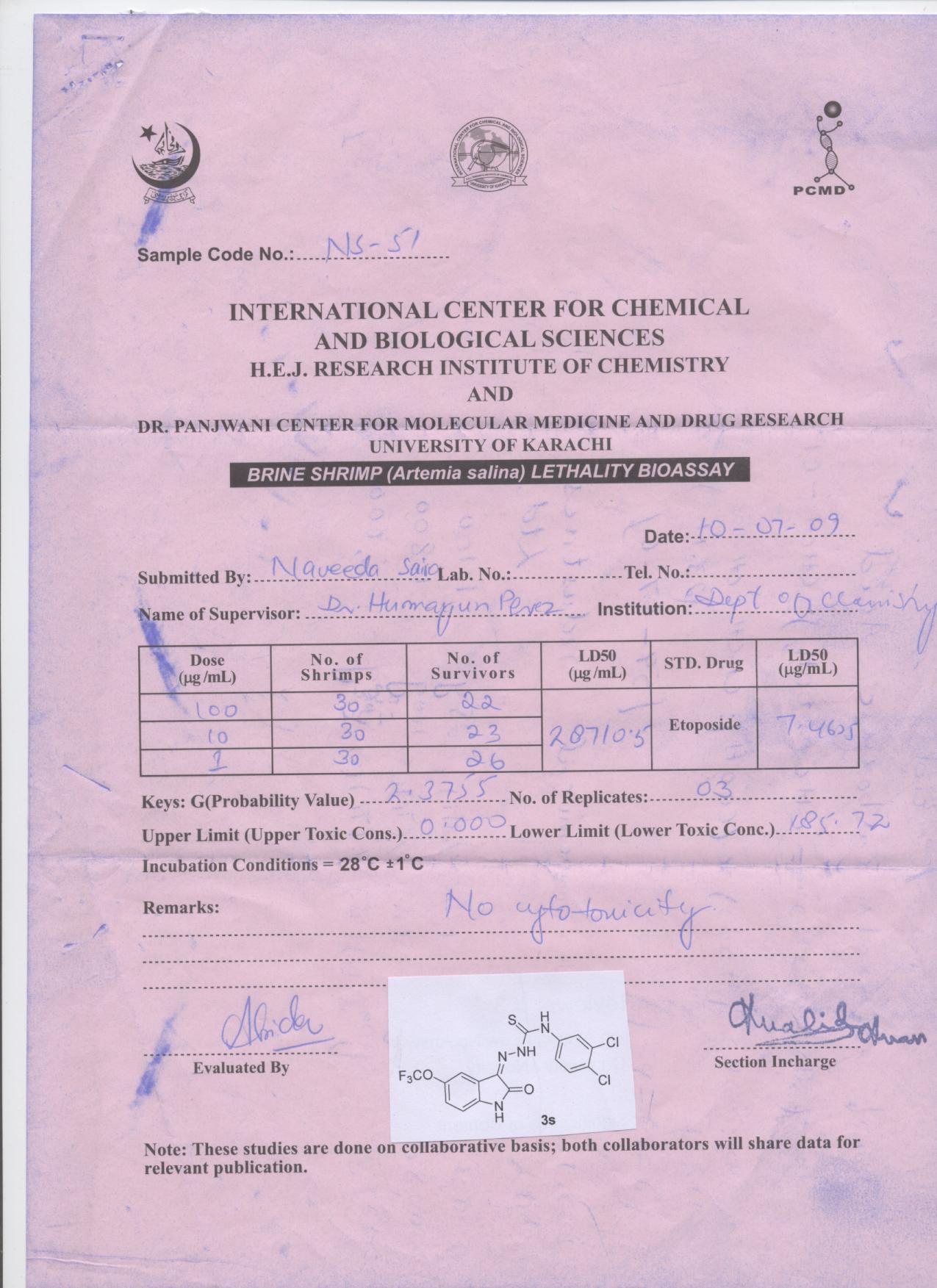

Supplement: Supplementary File 1 [file molecules-16-06408-s001.zip › Spectroscopy/Cytotoxity/3s.jpg]

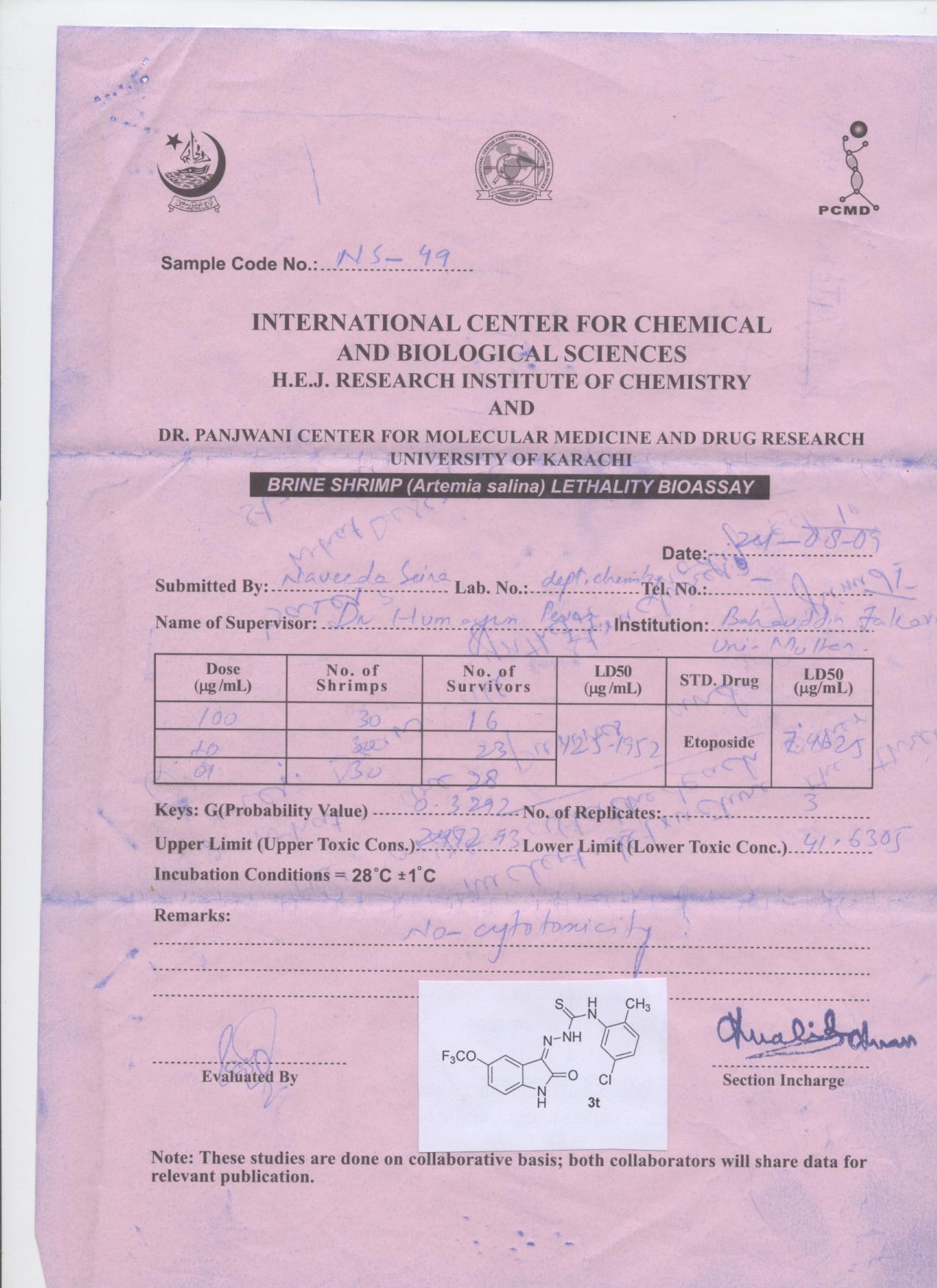

Supplement: Supplementary File 1 [file molecules-16-06408-s001.zip › Spectroscopy/Cytotoxity/3t.jpg]

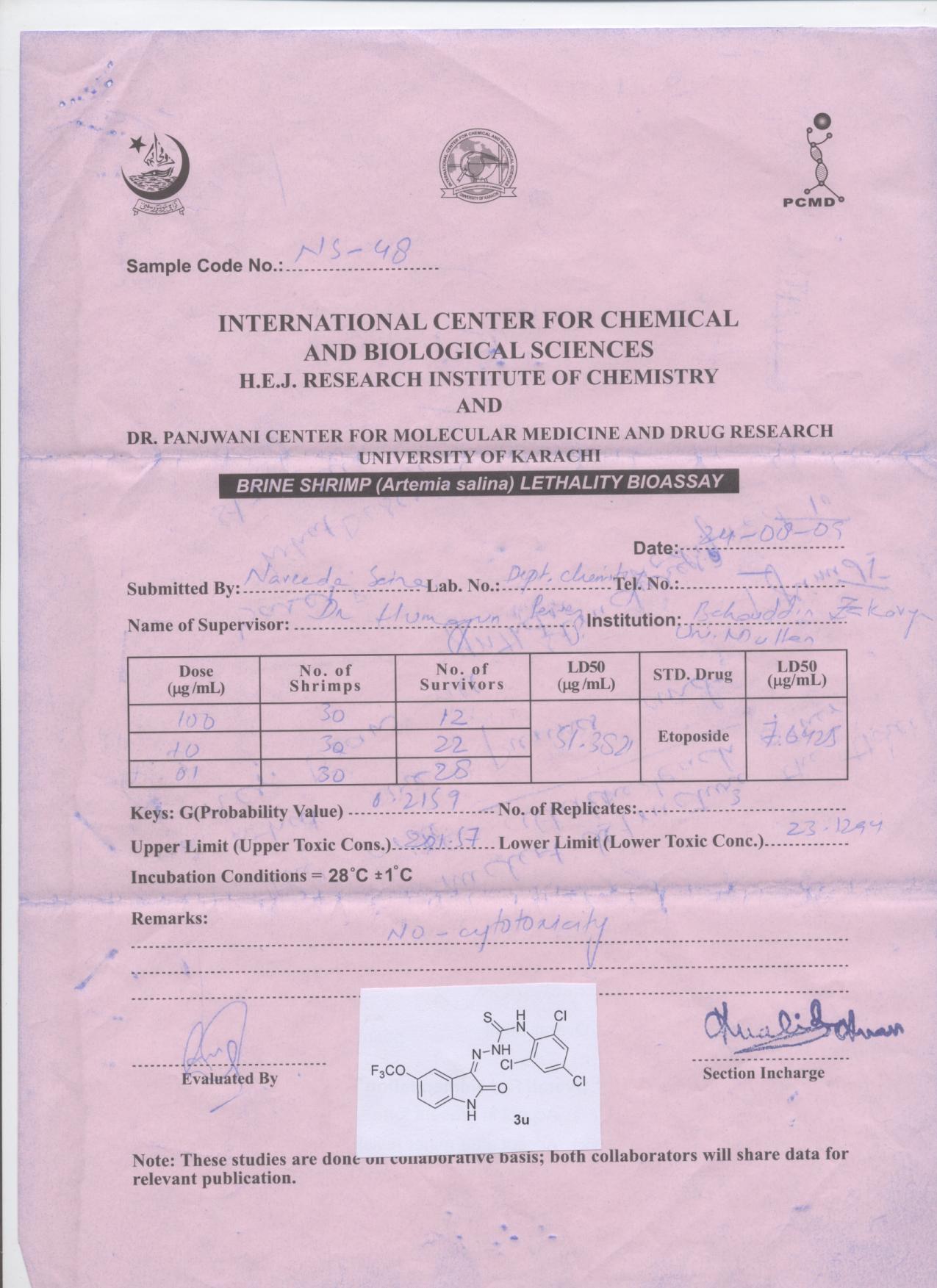

Supplement: Supplementary File 1 [file molecules-16-06408-s001.zip › Spectroscopy/Cytotoxity/3u.jpg]

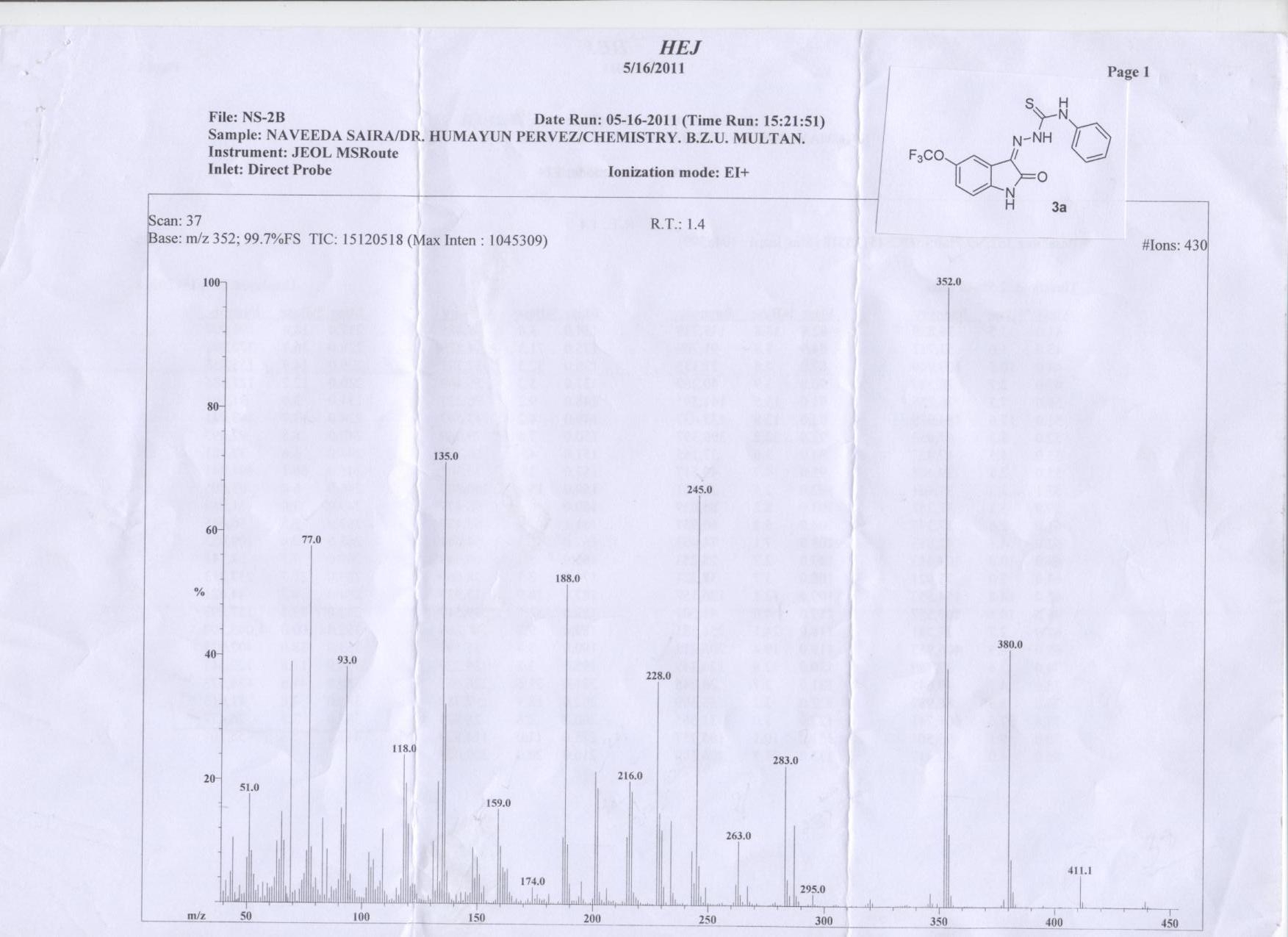

Supplement: Supplementary File 1 [file molecules-16-06408-s001.zip › Spectroscopy/MASS/3a.jpg]

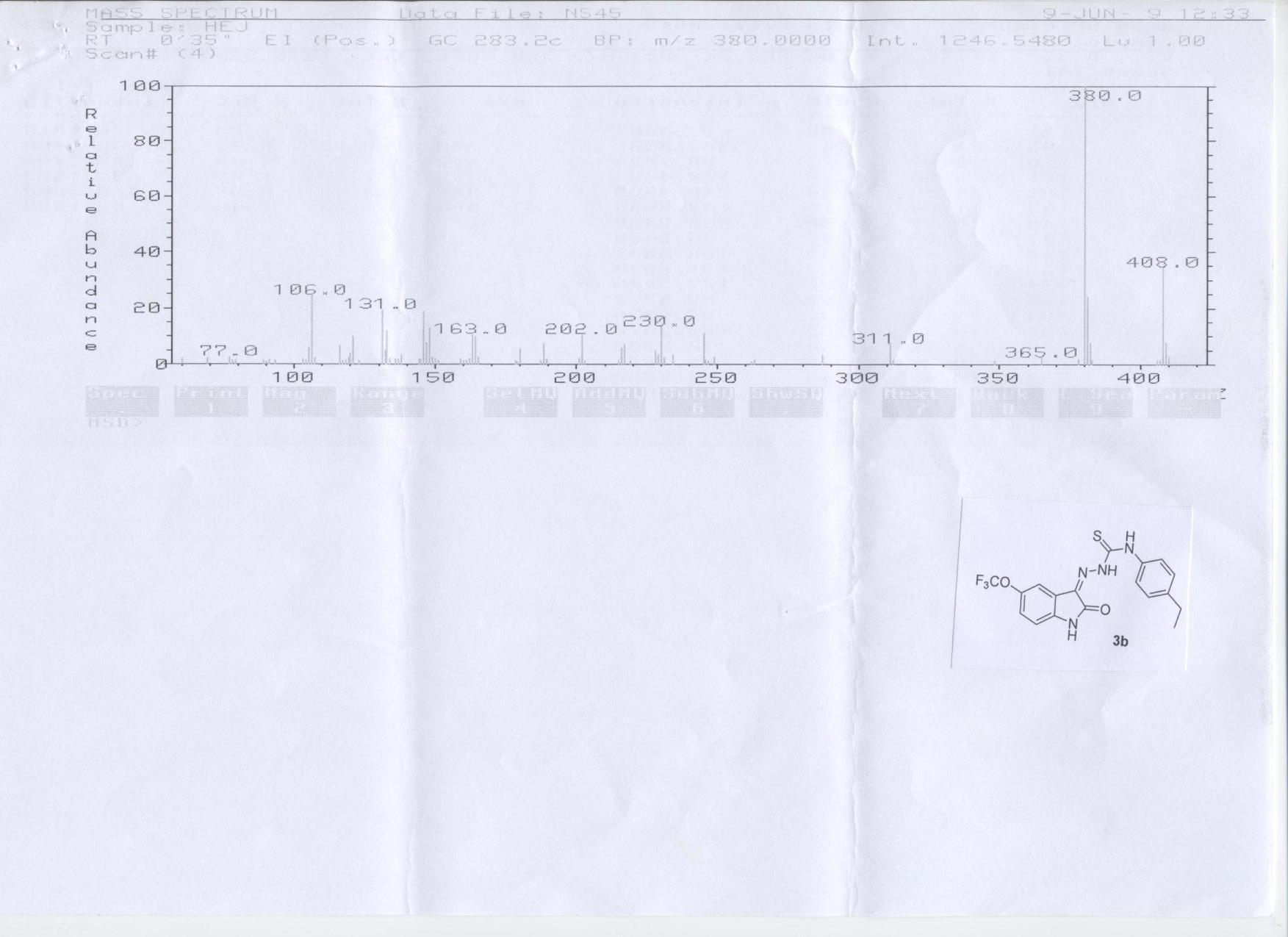

Supplement: Supplementary File 1 [file molecules-16-06408-s001.zip › Spectroscopy/MASS/3b.jpg]

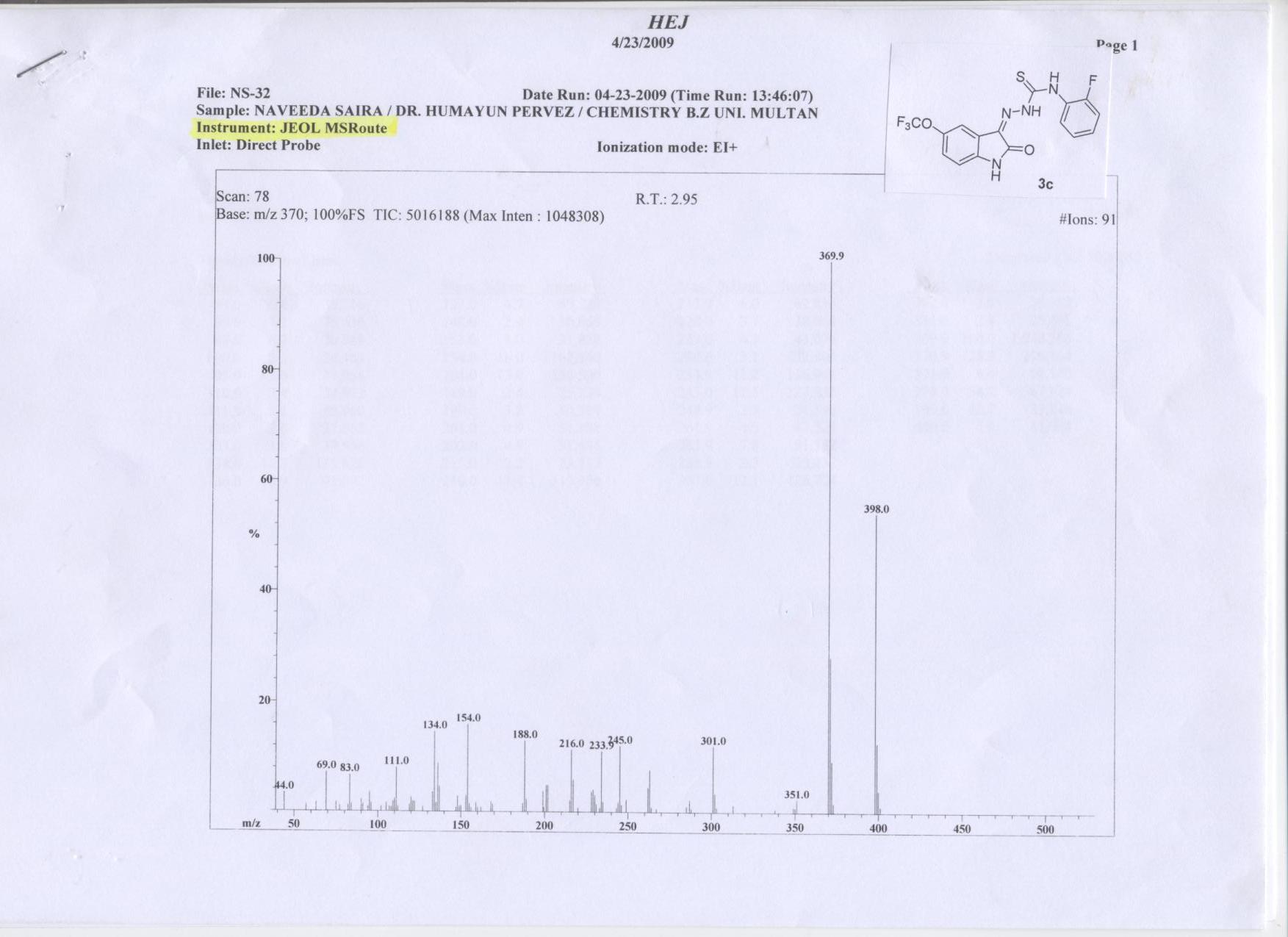

Supplement: Supplementary File 1 [file molecules-16-06408-s001.zip › Spectroscopy/MASS/3c.jpg]

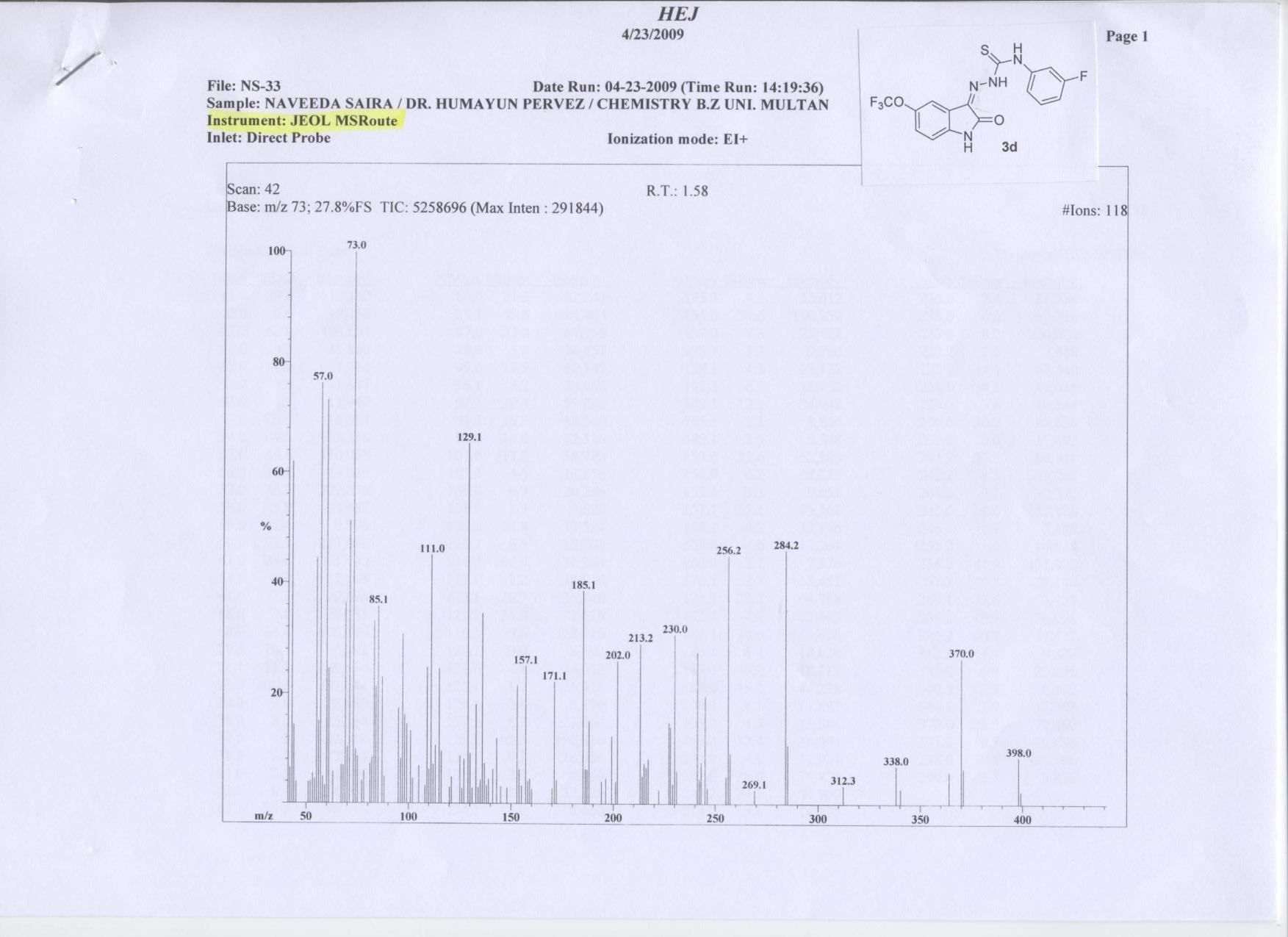

Supplement: Supplementary File 1 [file molecules-16-06408-s001.zip › Spectroscopy/MASS/3d.jpg]

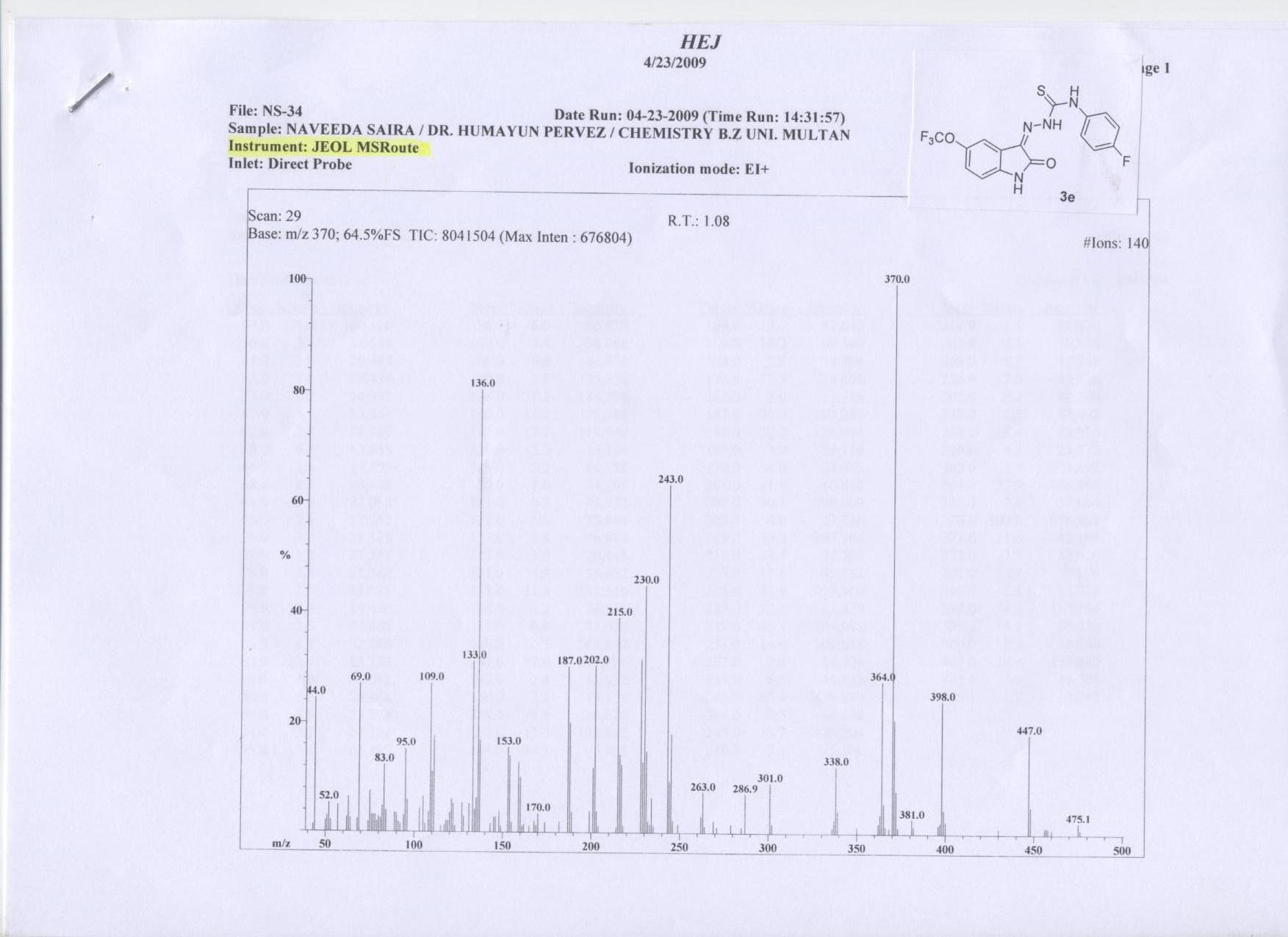

Supplement: Supplementary File 1 [file molecules-16-06408-s001.zip › Spectroscopy/MASS/3e.jpg]

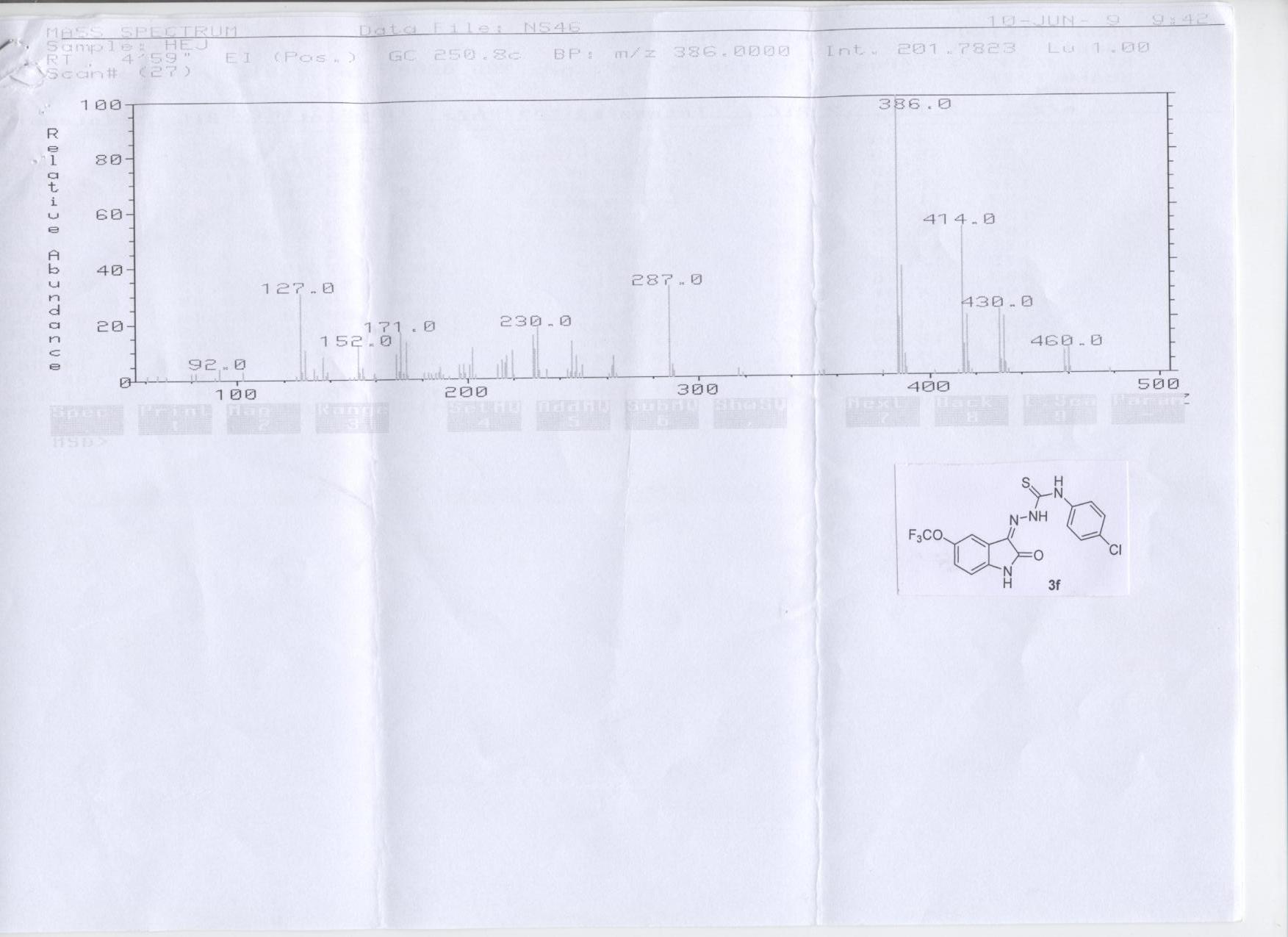

Supplement: Supplementary File 1 [file molecules-16-06408-s001.zip › Spectroscopy/MASS/3f.jpg]

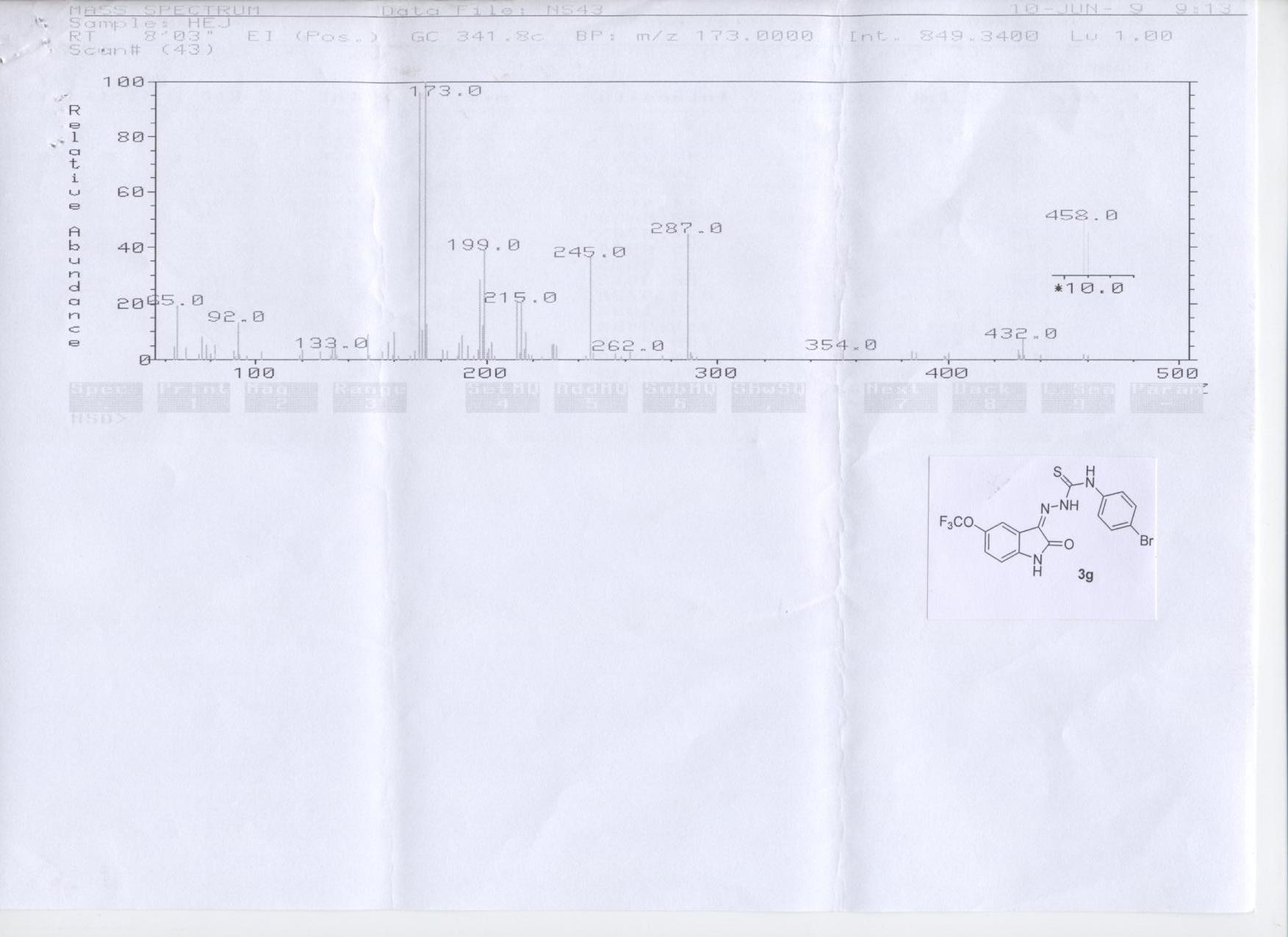

Supplement: Supplementary File 1 [file molecules-16-06408-s001.zip › Spectroscopy/MASS/3g.jpg]

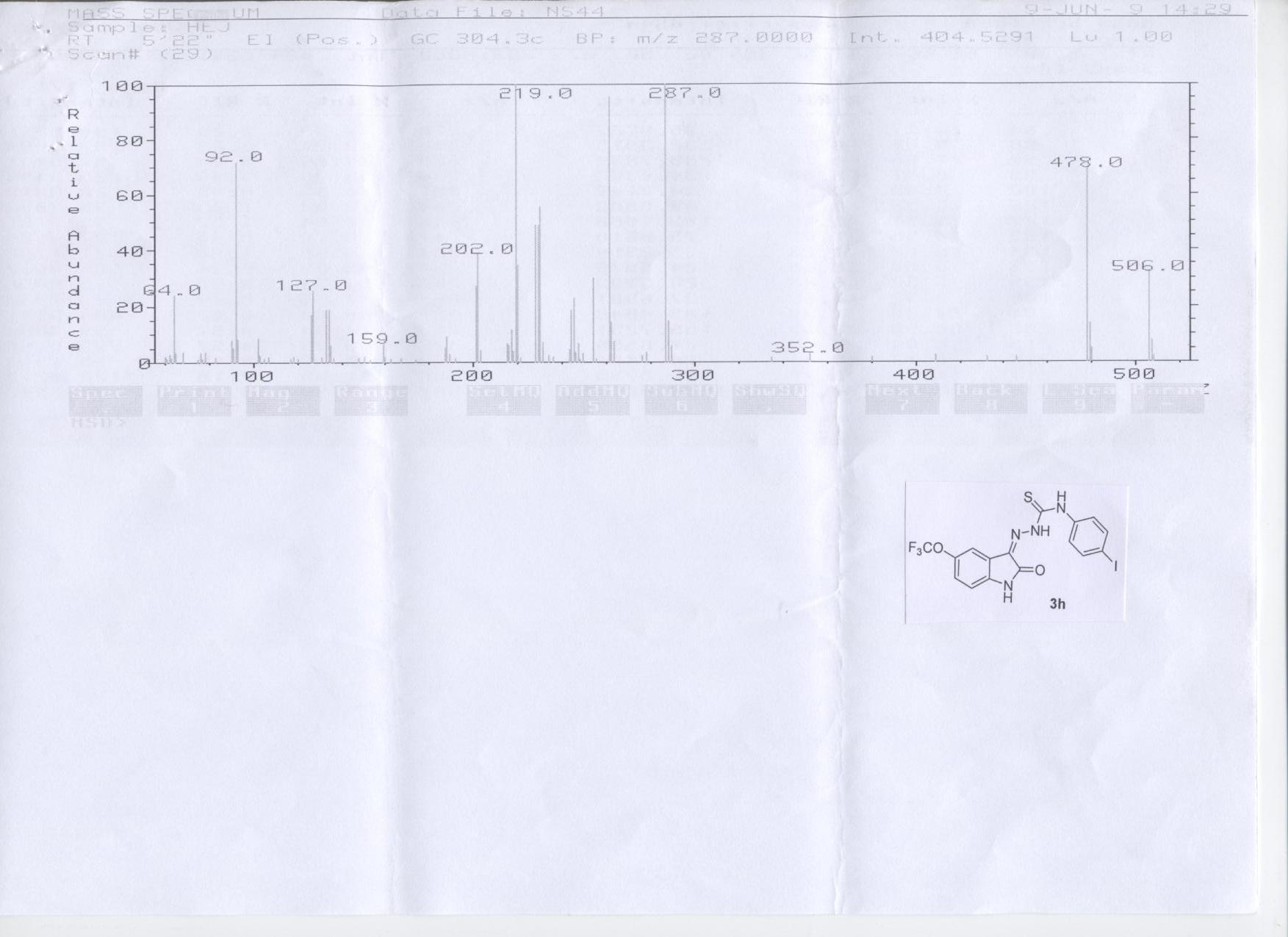

Supplement: Supplementary File 1 [file molecules-16-06408-s001.zip › Spectroscopy/MASS/3h.jpg]

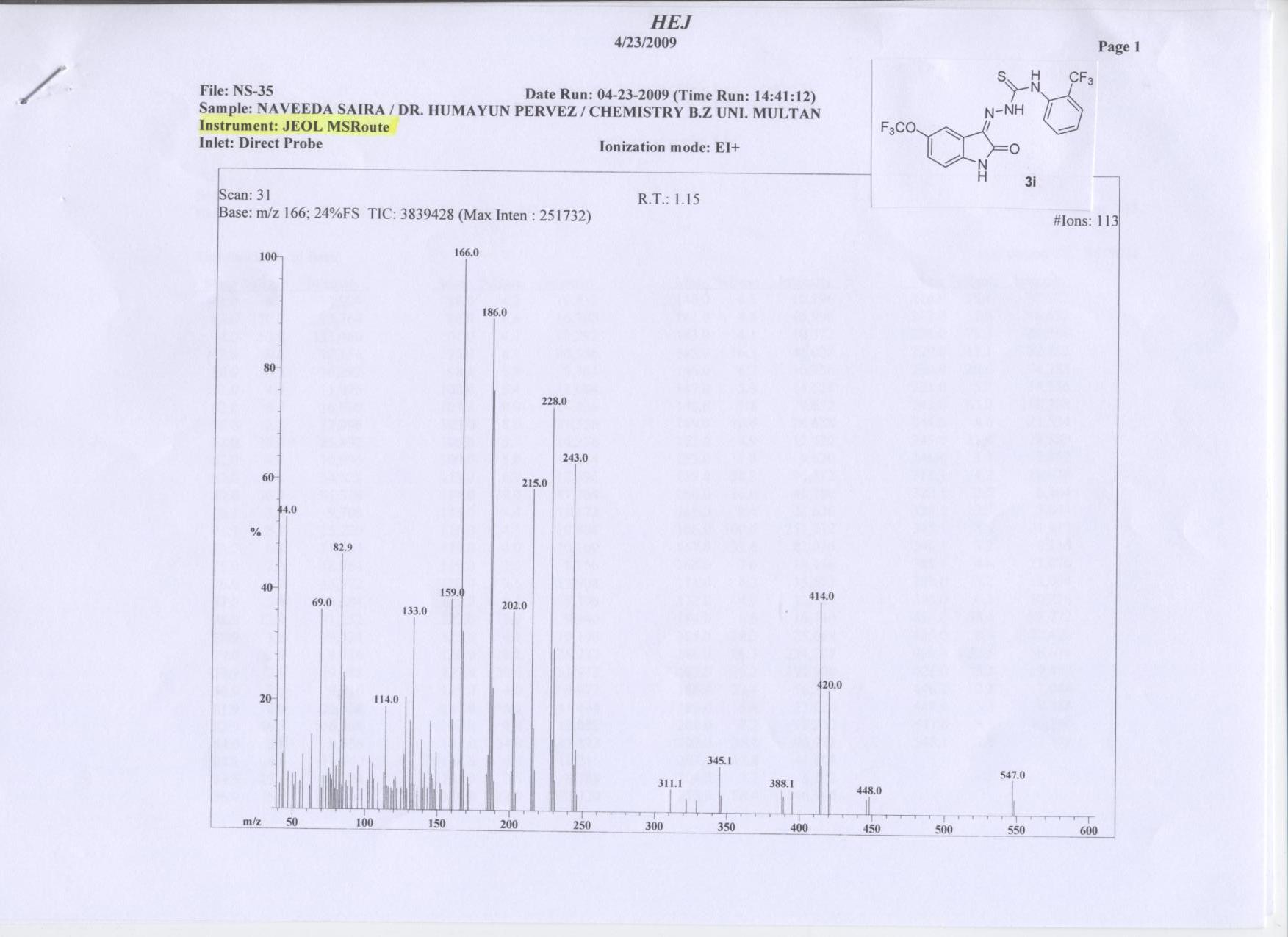

Supplement: Supplementary File 1 [file molecules-16-06408-s001.zip › Spectroscopy/MASS/3i.jpg]

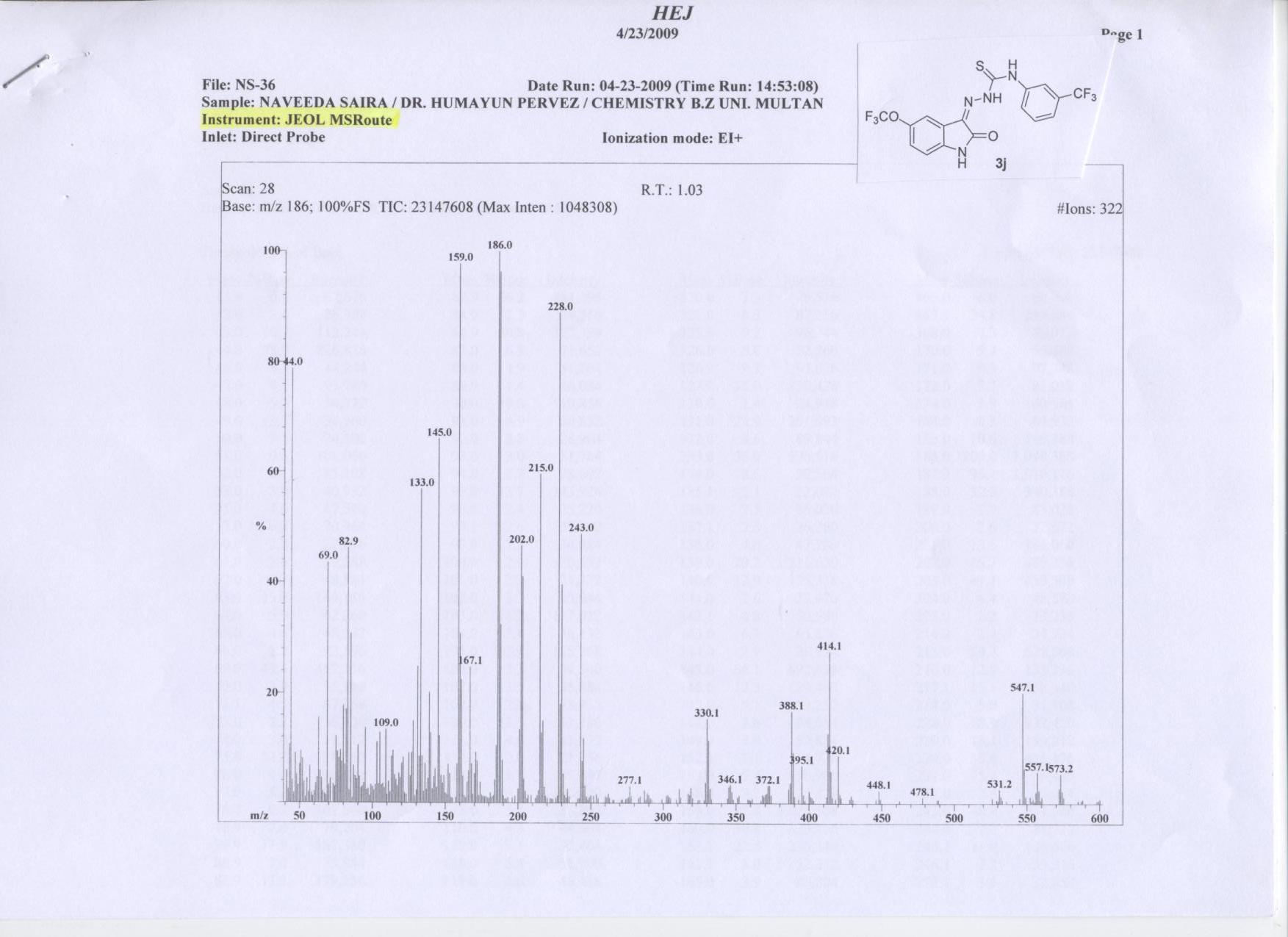

Supplement: Supplementary File 1 [file molecules-16-06408-s001.zip › Spectroscopy/MASS/3j.jpg]

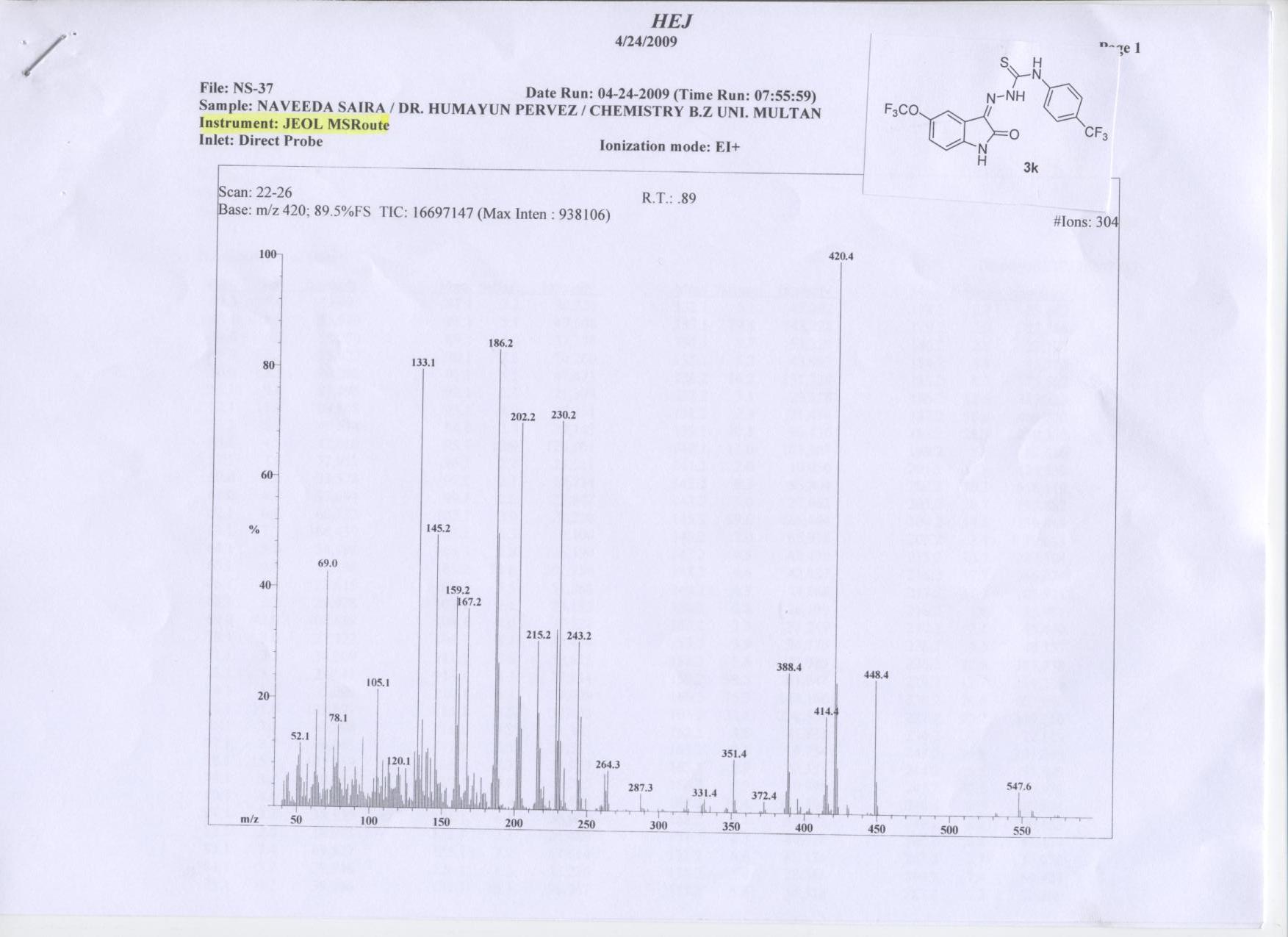

Supplement: Supplementary File 1 [file molecules-16-06408-s001.zip › Spectroscopy/MASS/3k.jpg]

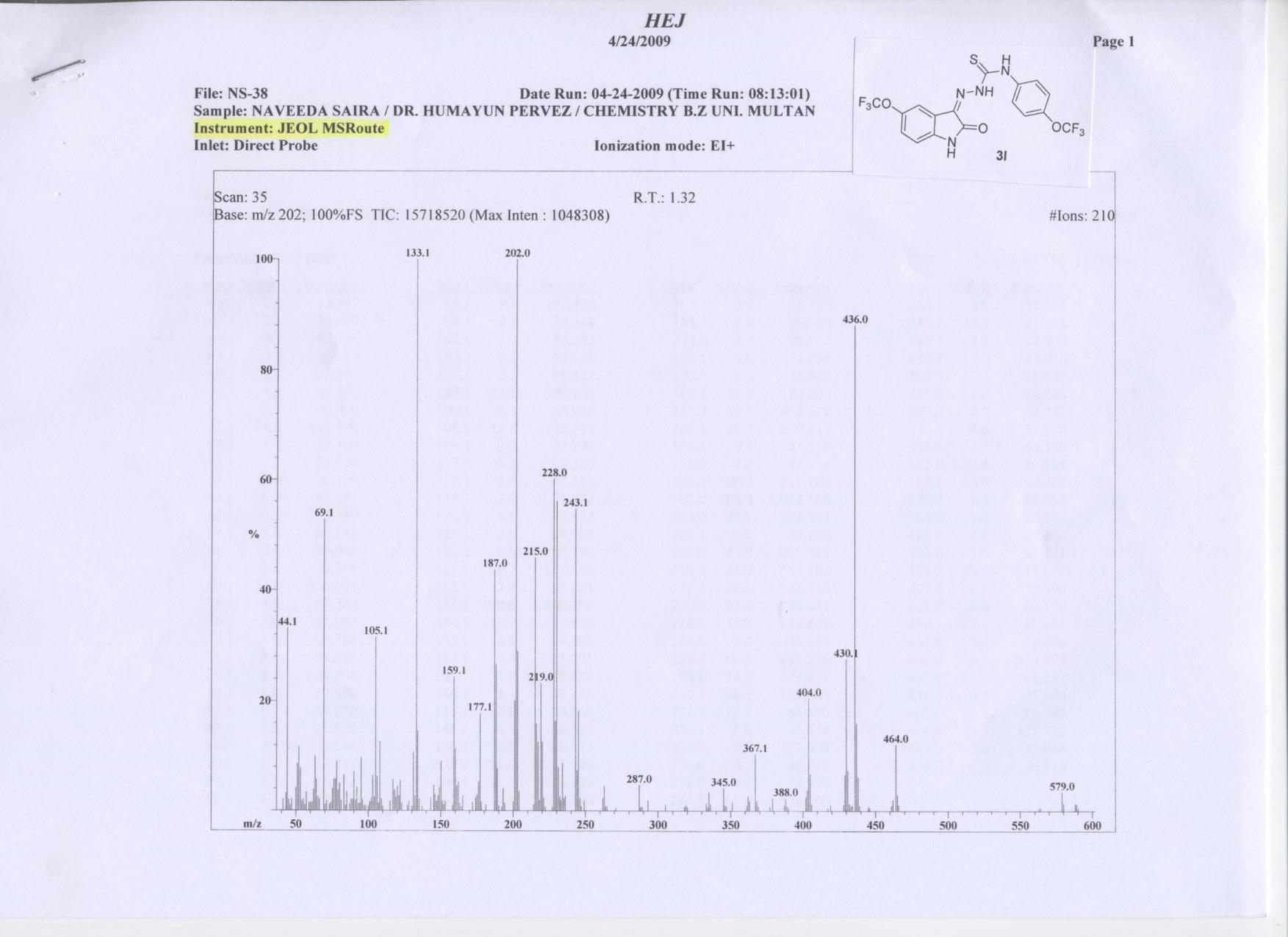

Supplement: Supplementary File 1 [file molecules-16-06408-s001.zip › Spectroscopy/MASS/3l.jpg]

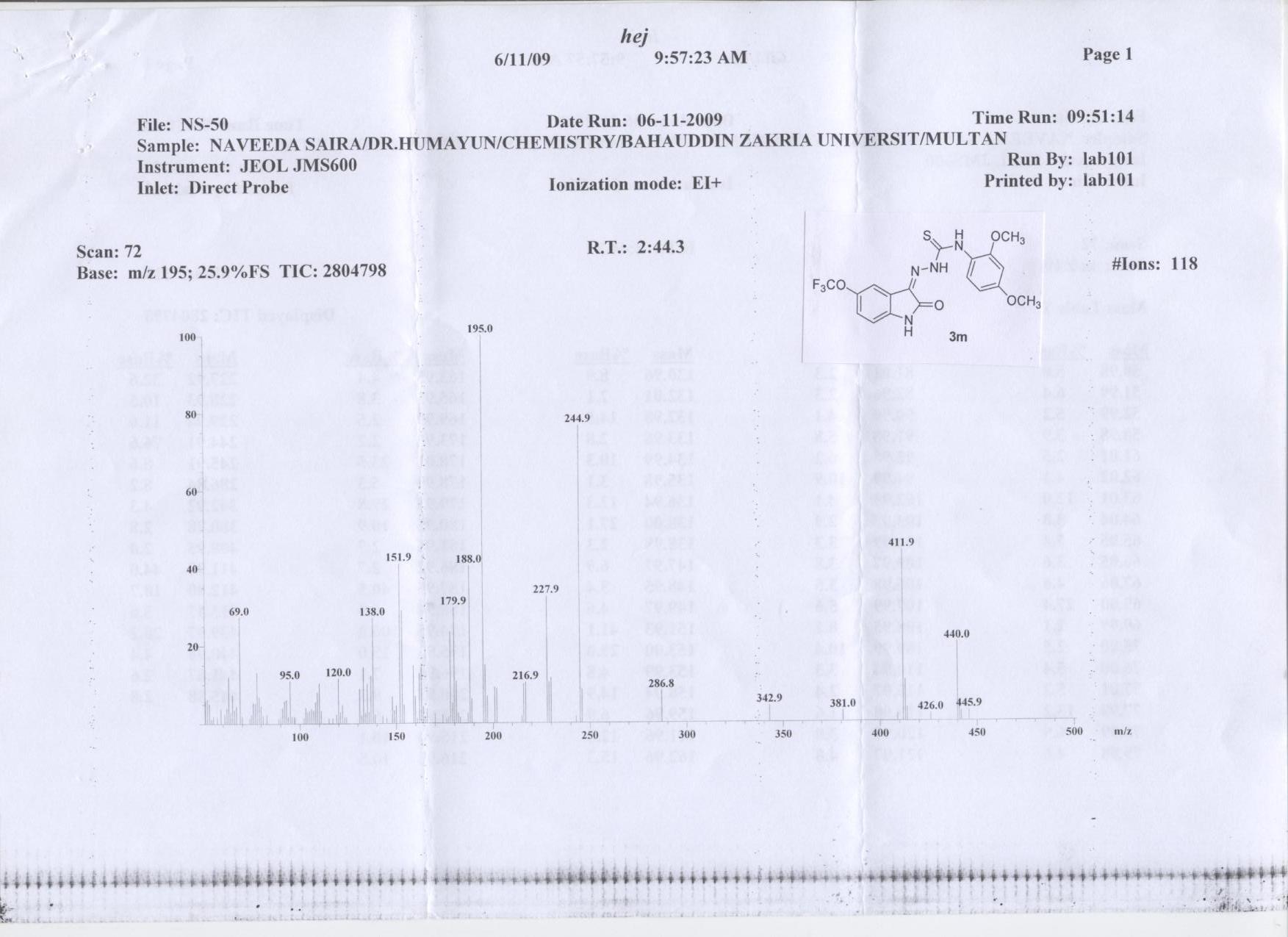

Supplement: Supplementary File 1 [file molecules-16-06408-s001.zip › Spectroscopy/MASS/3m.jpg]

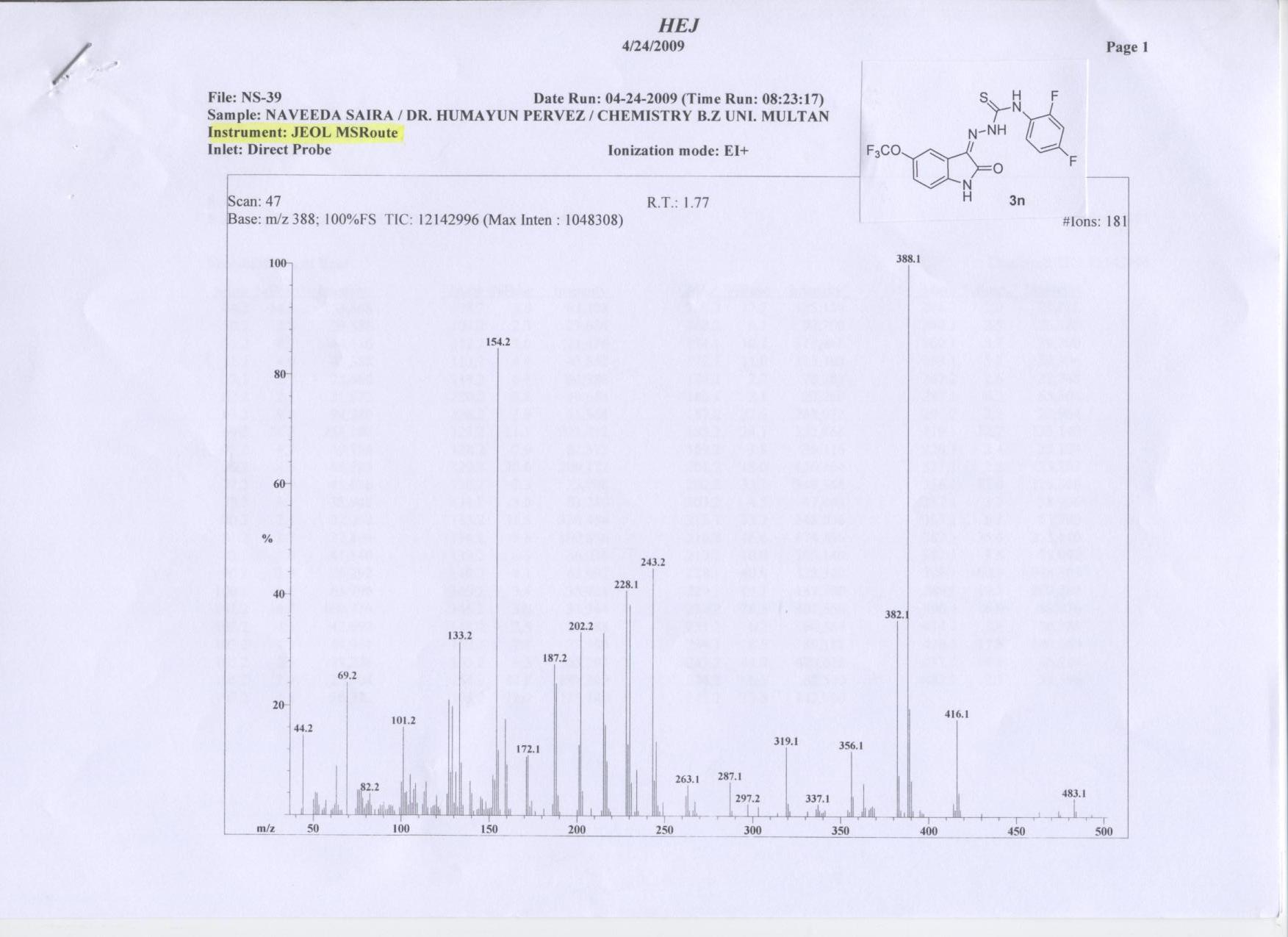

Supplement: Supplementary File 1 [file molecules-16-06408-s001.zip › Spectroscopy/MASS/3n.jpg]

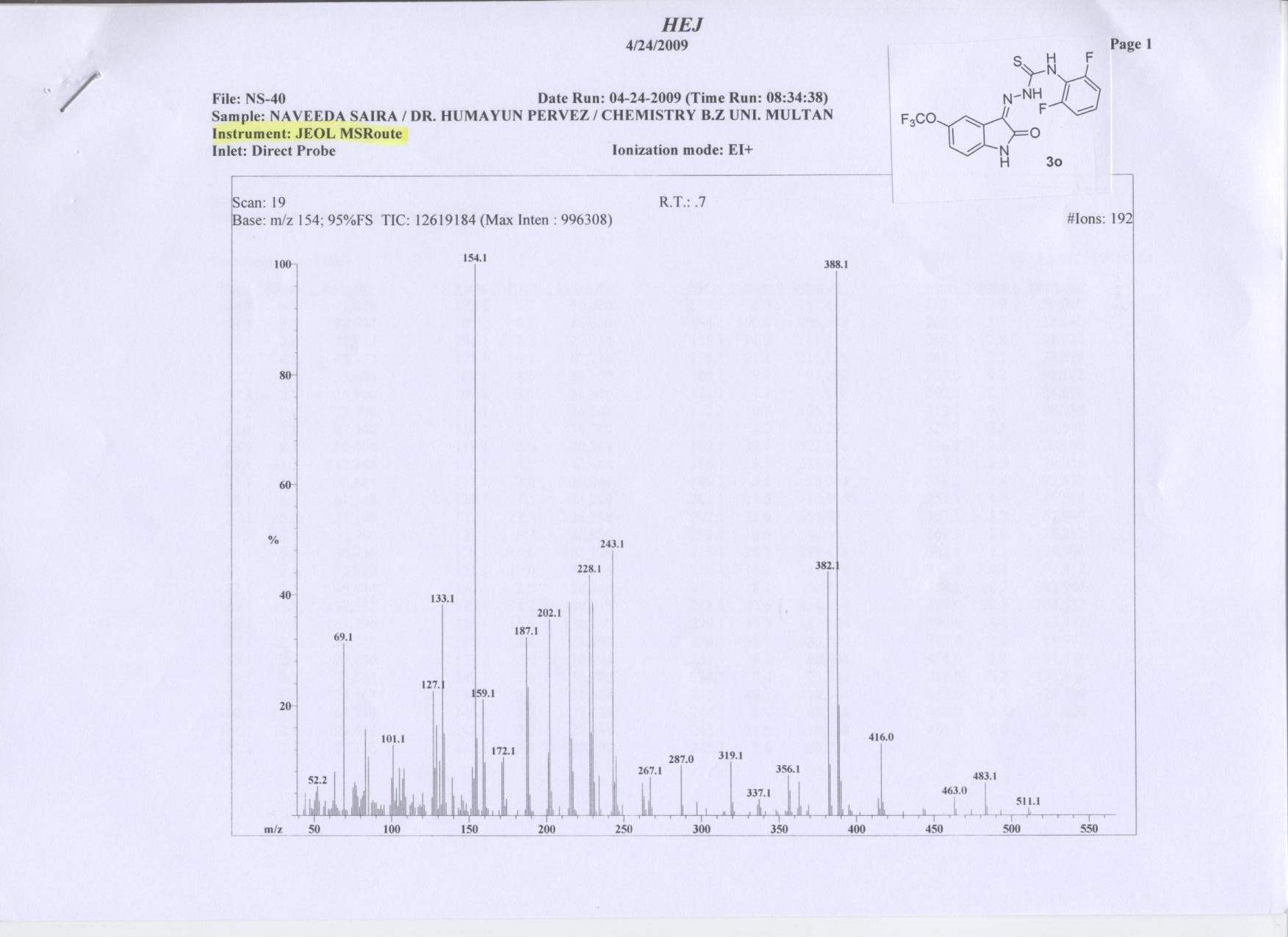

Supplement: Supplementary File 1 [file molecules-16-06408-s001.zip › Spectroscopy/MASS/3o.jpg]

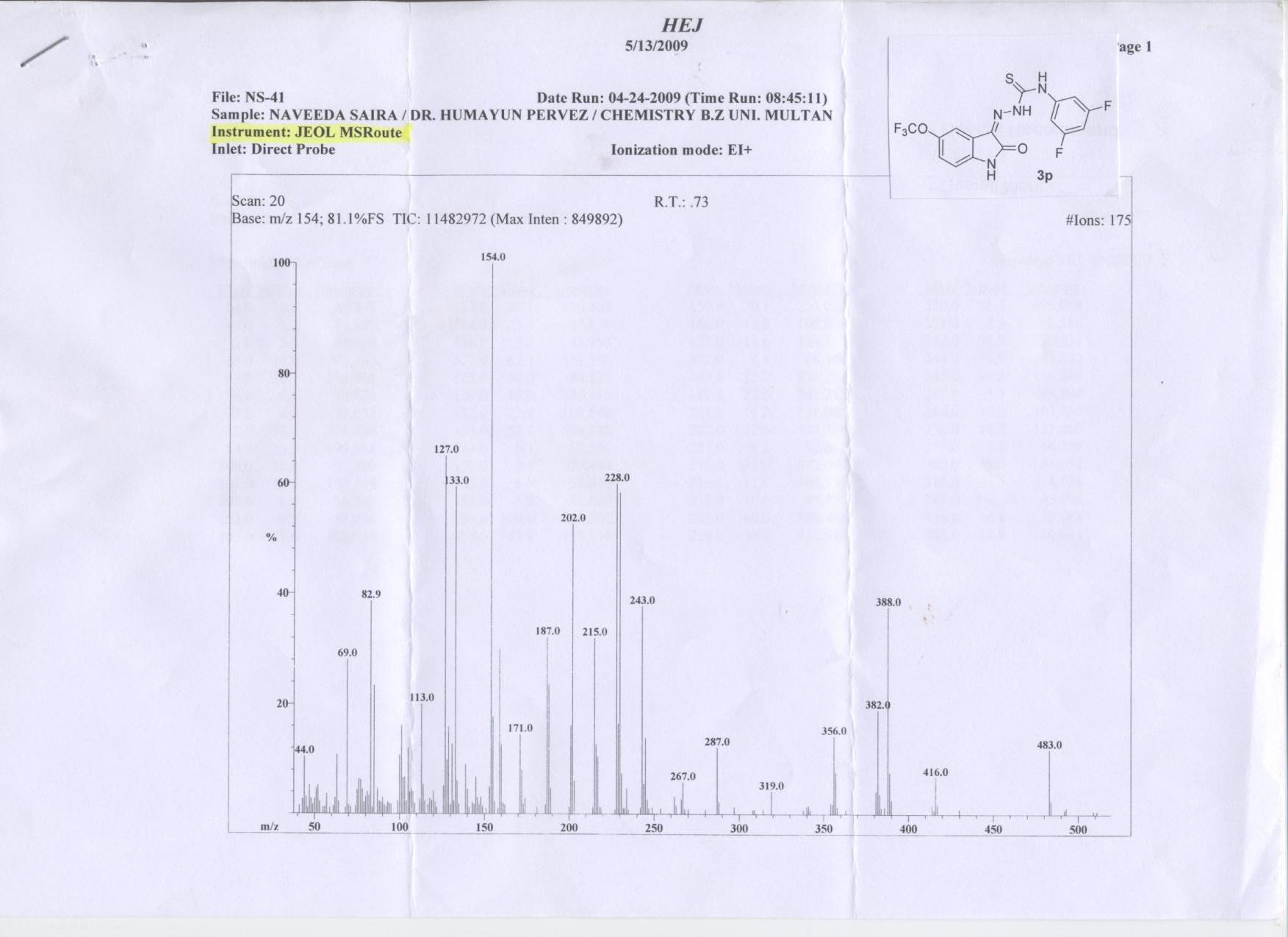

Supplement: Supplementary File 1 [file molecules-16-06408-s001.zip › Spectroscopy/MASS/3p.jpg]

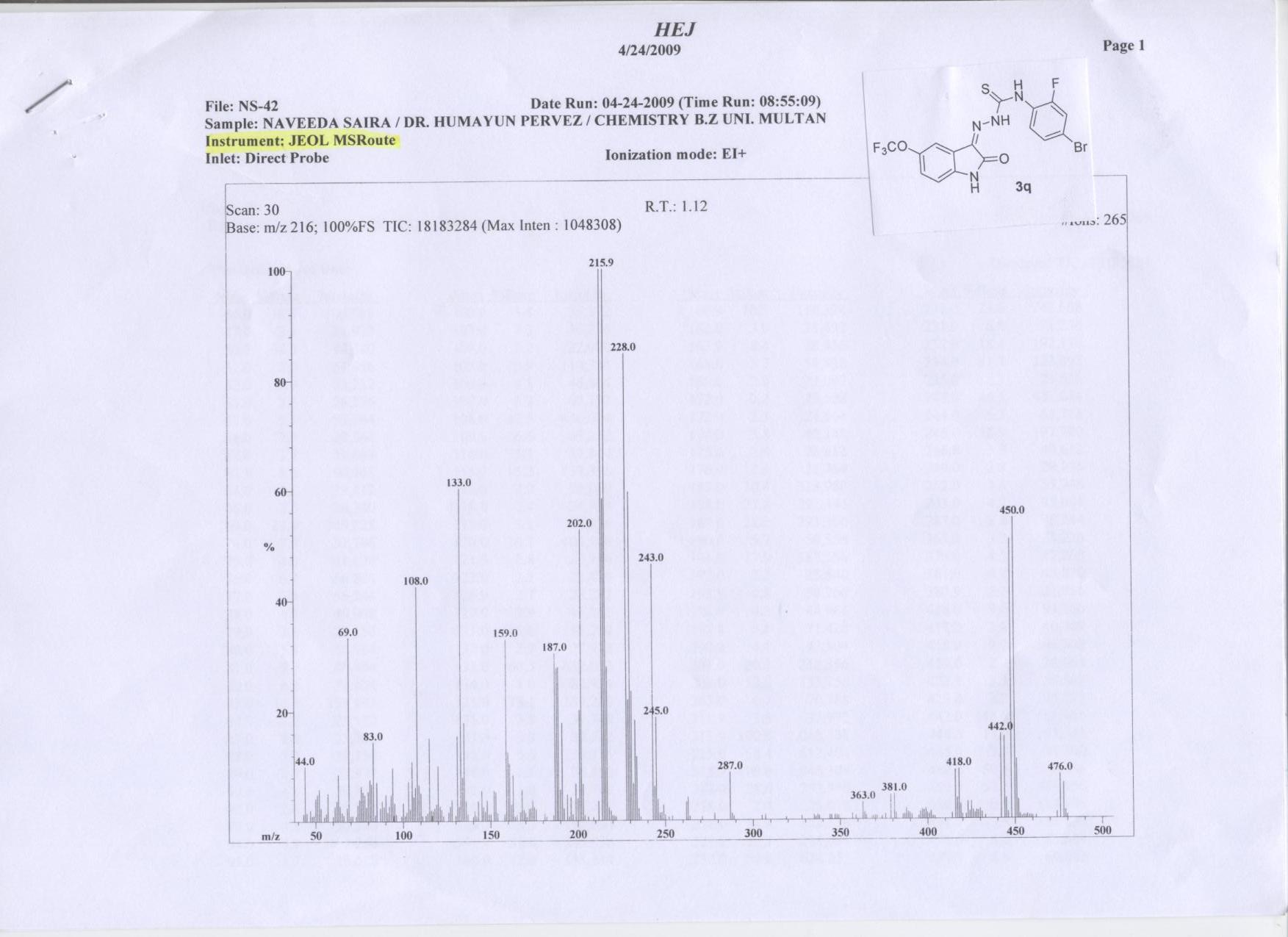

Supplement: Supplementary File 1 [file molecules-16-06408-s001.zip › Spectroscopy/MASS/3q.jpg]

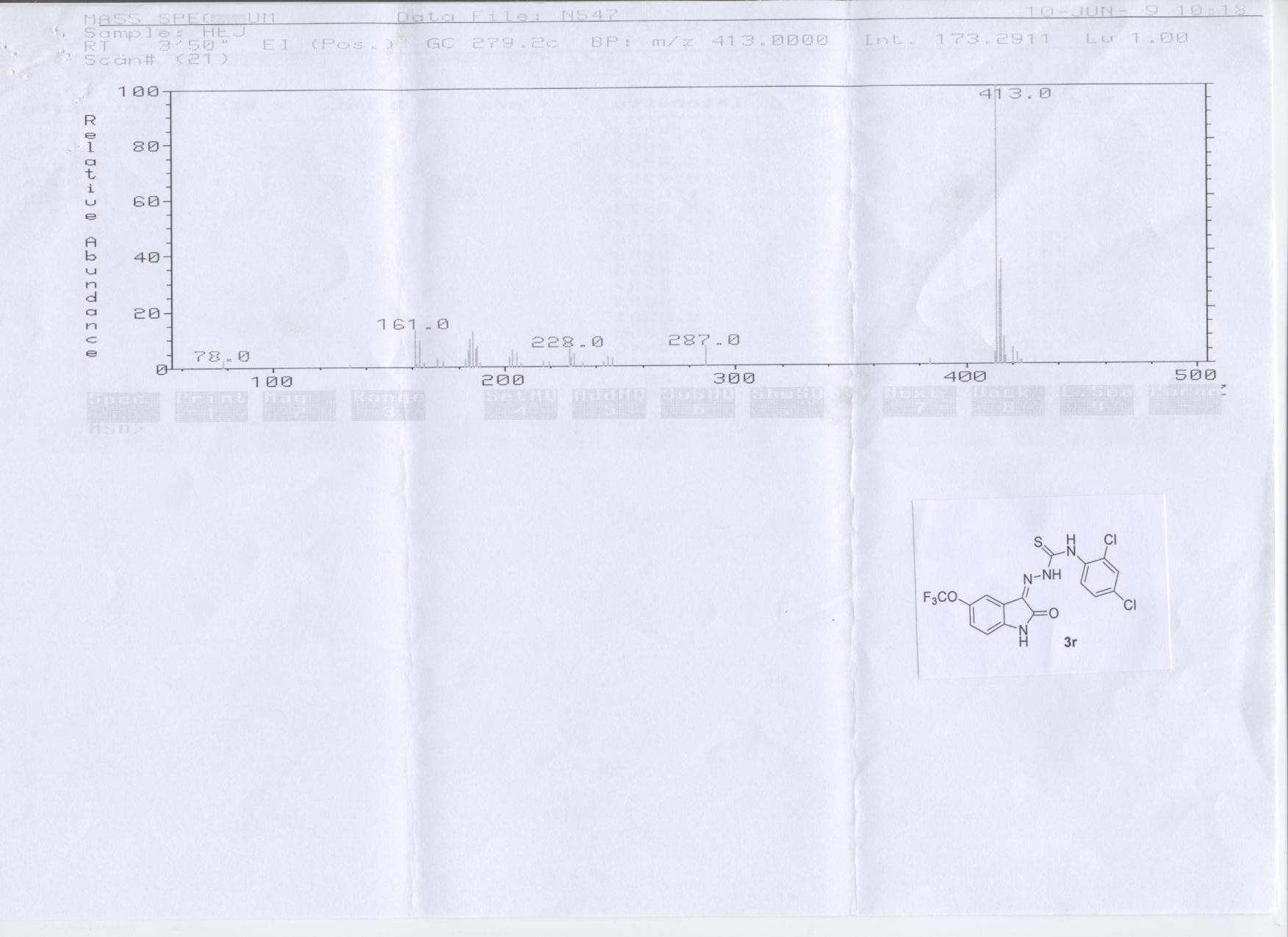

Supplement: Supplementary File 1 [file molecules-16-06408-s001.zip › Spectroscopy/MASS/3r.jpg]

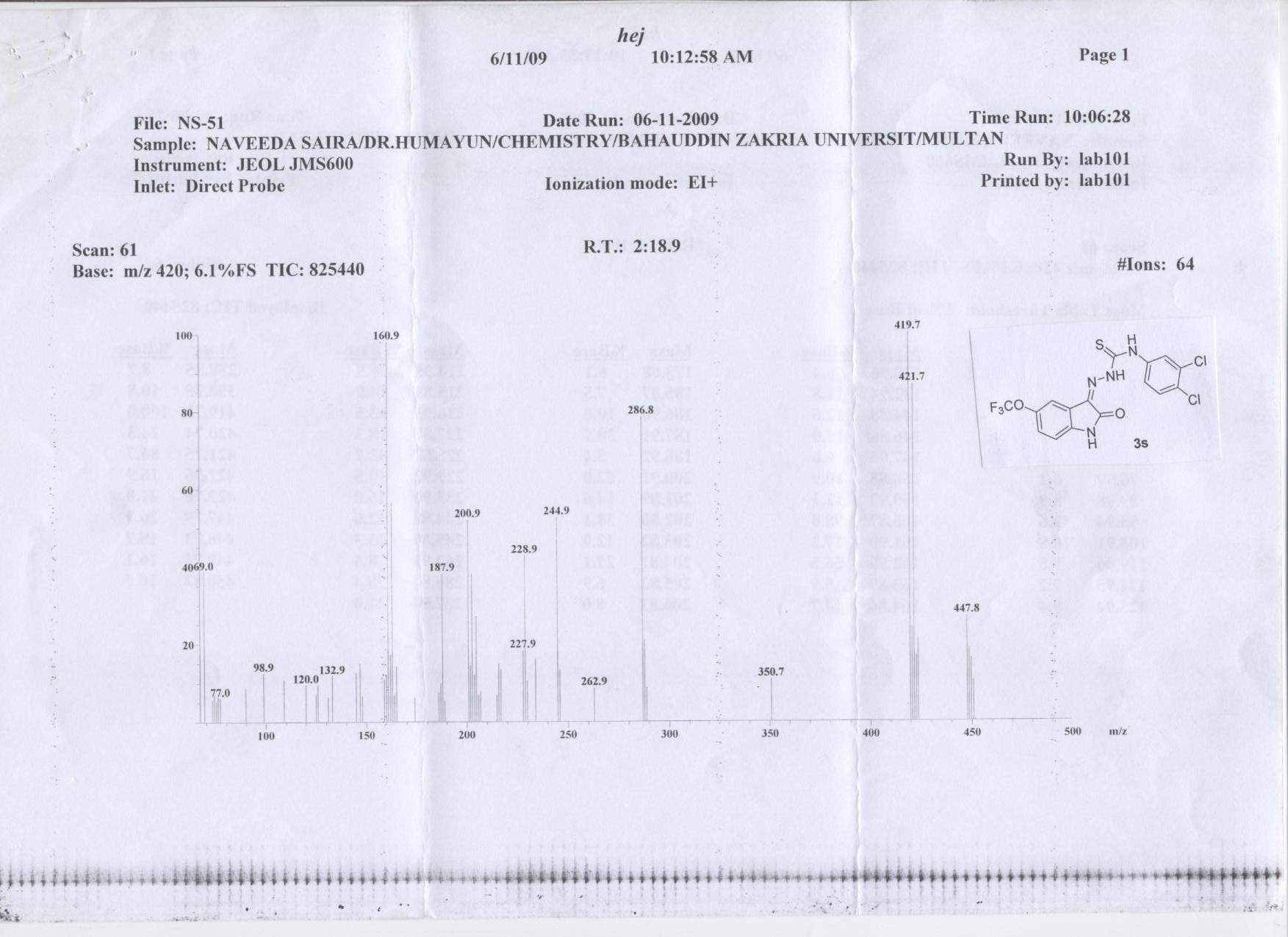

Supplement: Supplementary File 1 [file molecules-16-06408-s001.zip › Spectroscopy/MASS/3s.jpg]

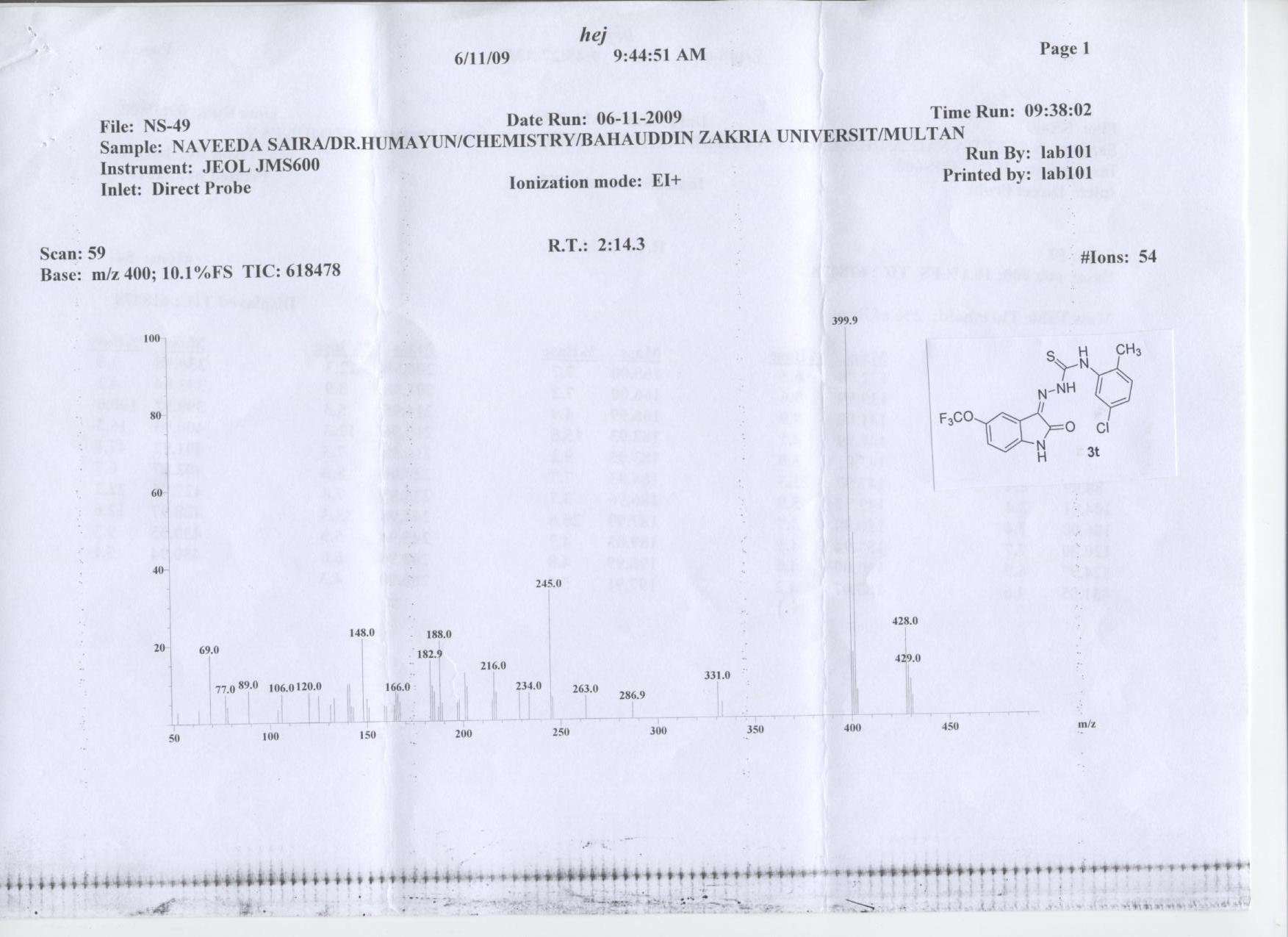

Supplement: Supplementary File 1 [file molecules-16-06408-s001.zip › Spectroscopy/MASS/3t.jpg]

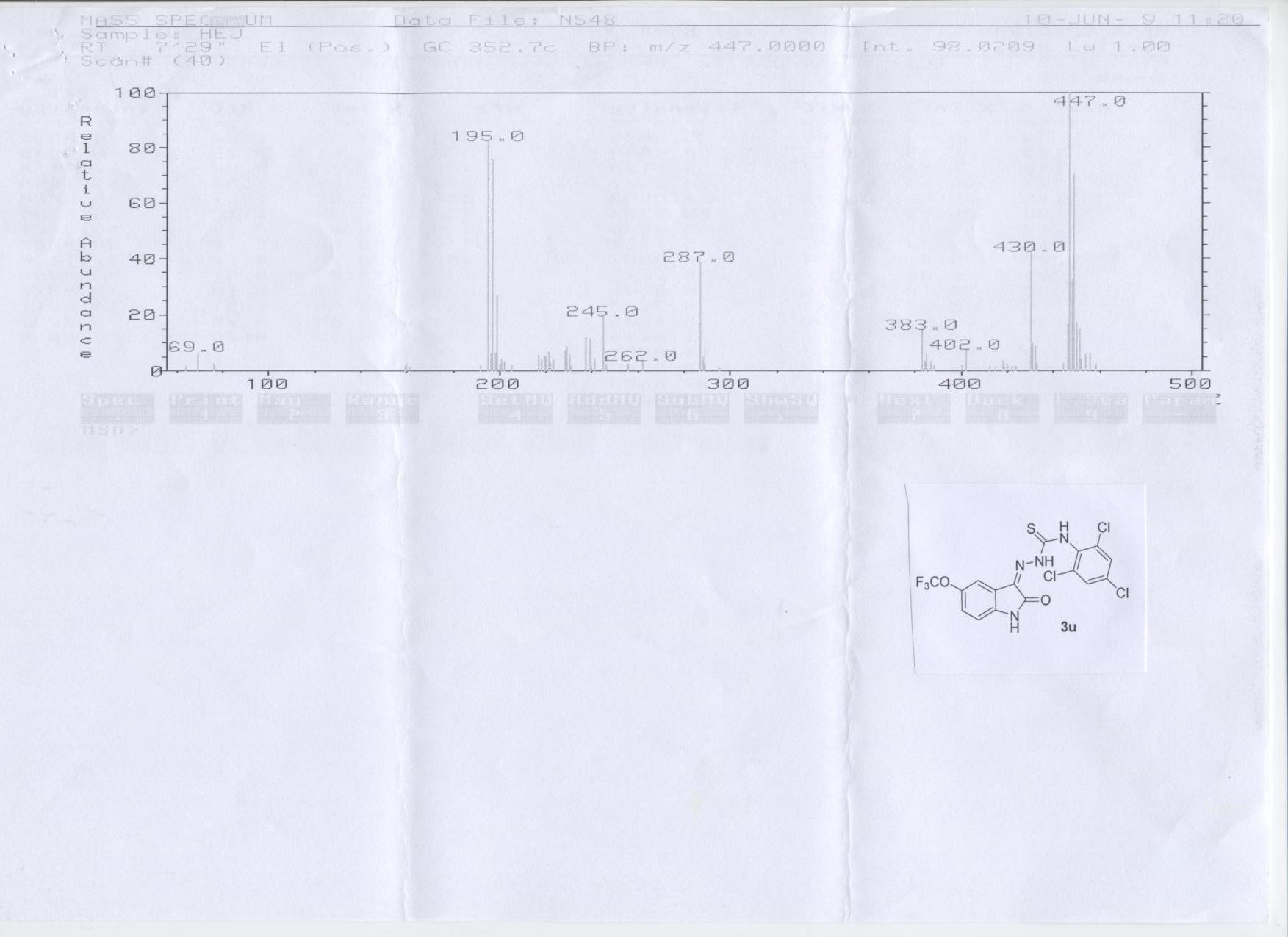

Supplement: Supplementary File 1 [file molecules-16-06408-s001.zip › Spectroscopy/MASS/3u.jpg]

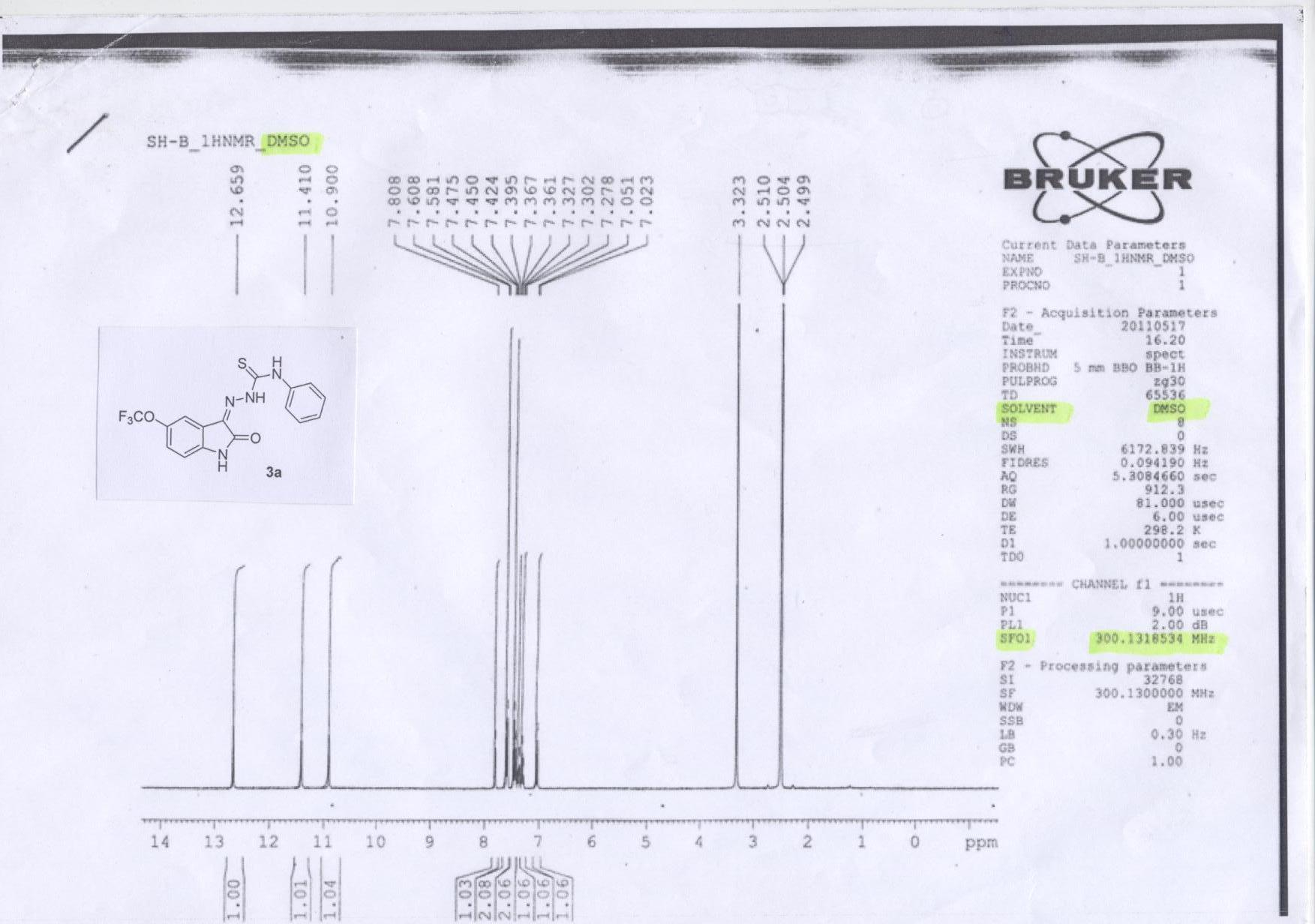

Supplement: Supplementary File 1 [file molecules-16-06408-s001.zip › Spectroscopy/NMR/3a.jpg]

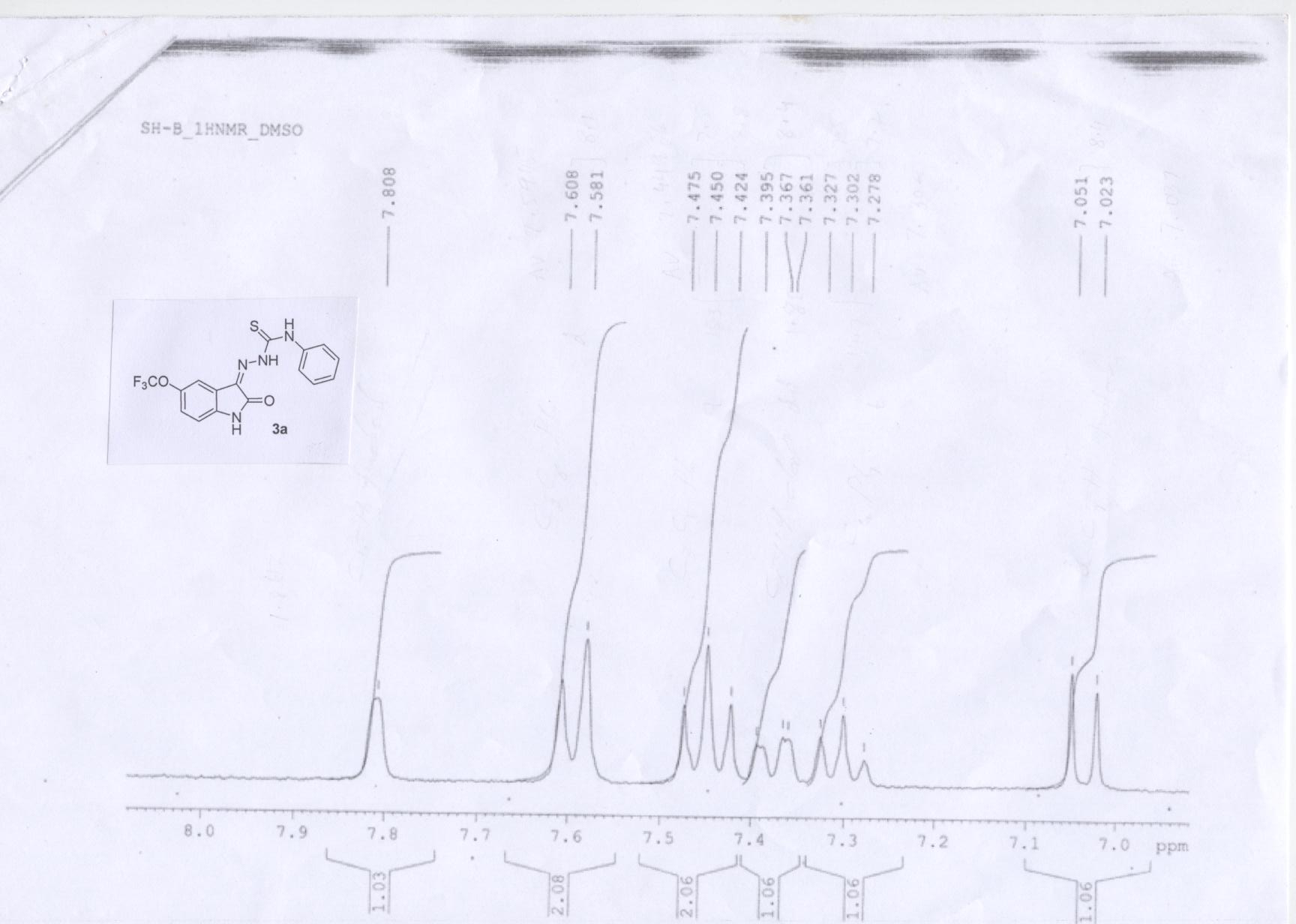

Supplement: Supplementary File 1 [file molecules-16-06408-s001.zip › Spectroscopy/NMR/3a1.jpg]

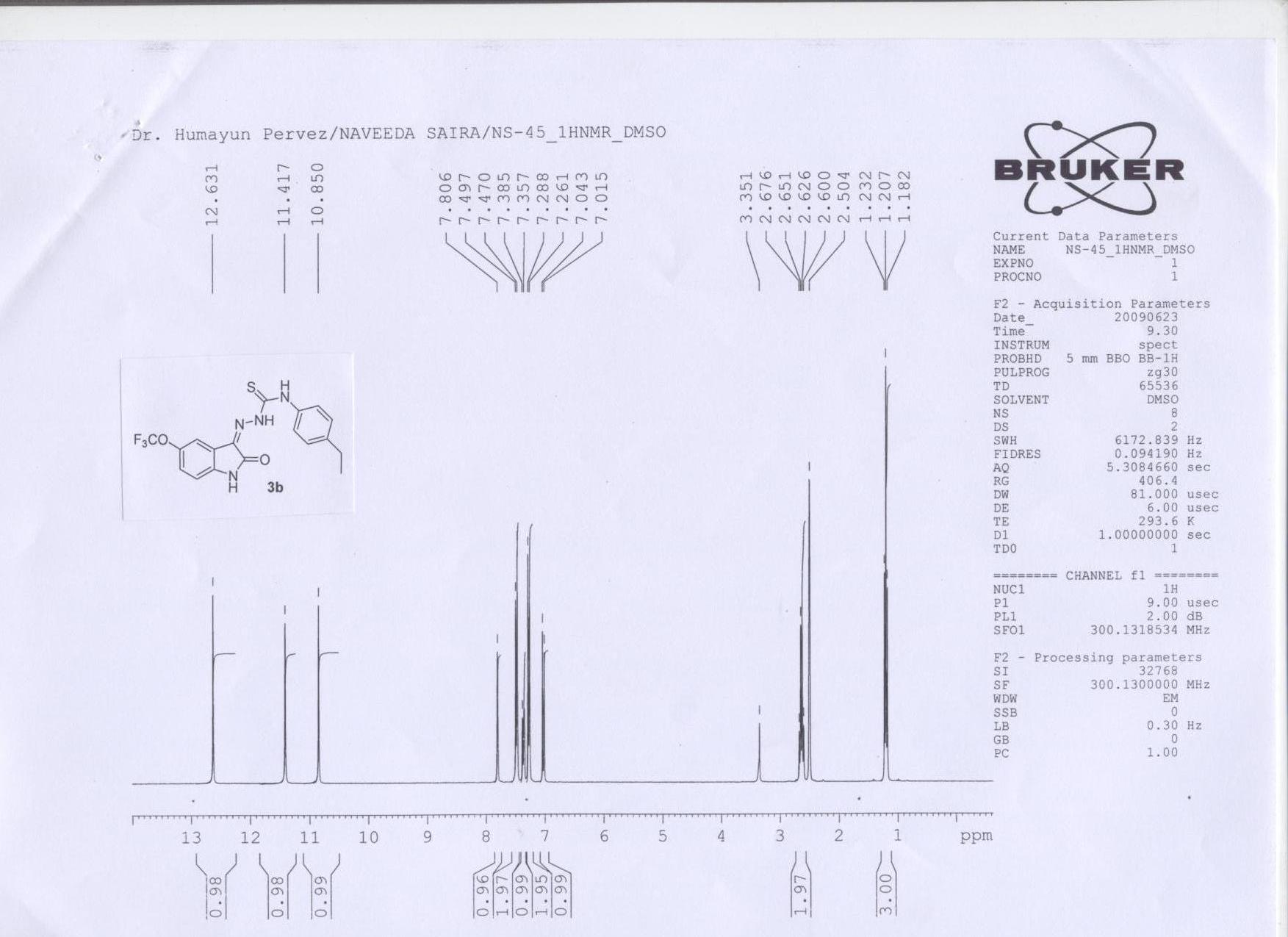

Supplement: Supplementary File 1 [file molecules-16-06408-s001.zip › Spectroscopy/NMR/3b.jpg]

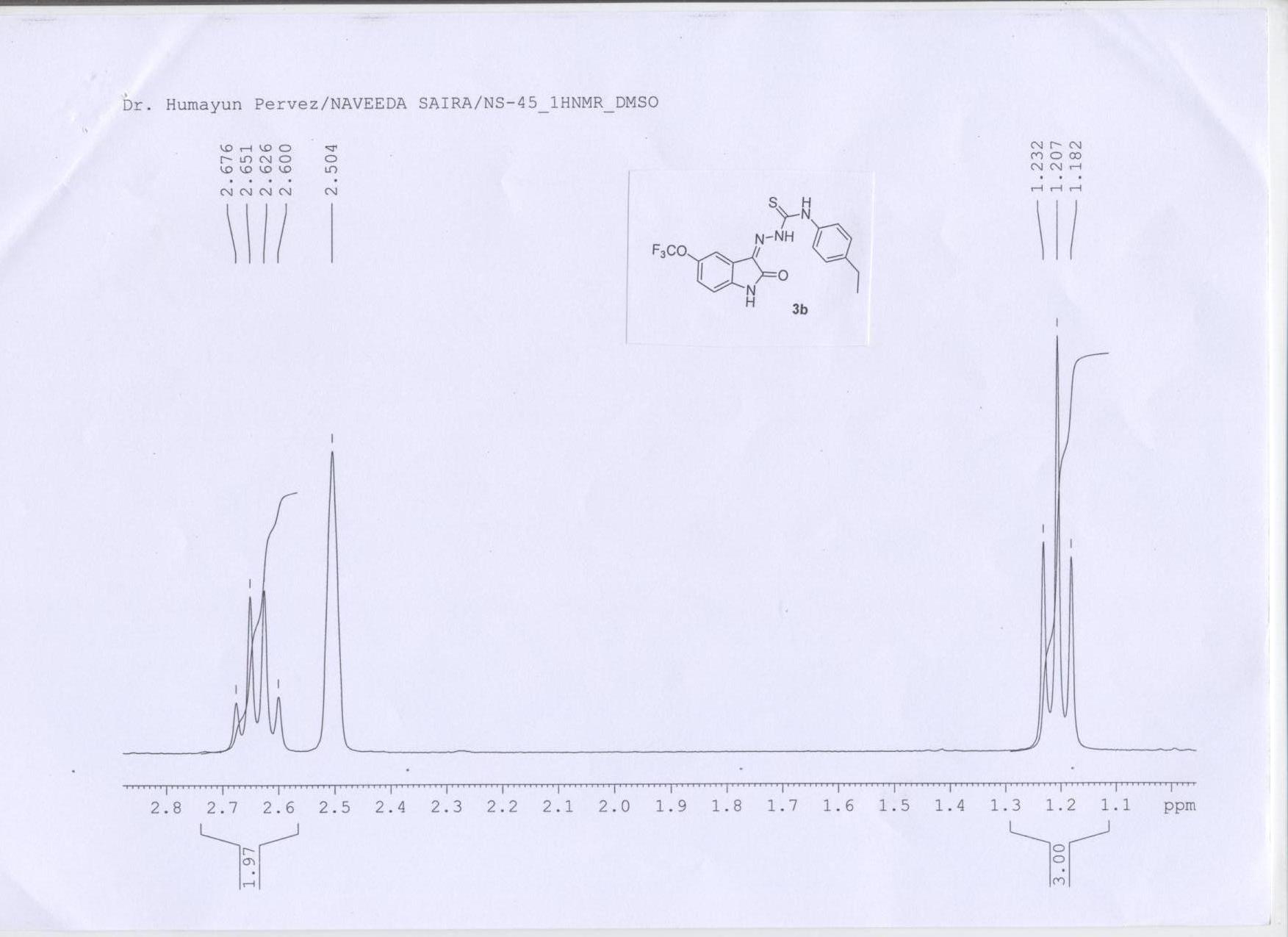

Supplement: Supplementary File 1 [file molecules-16-06408-s001.zip › Spectroscopy/NMR/3b1.jpg]

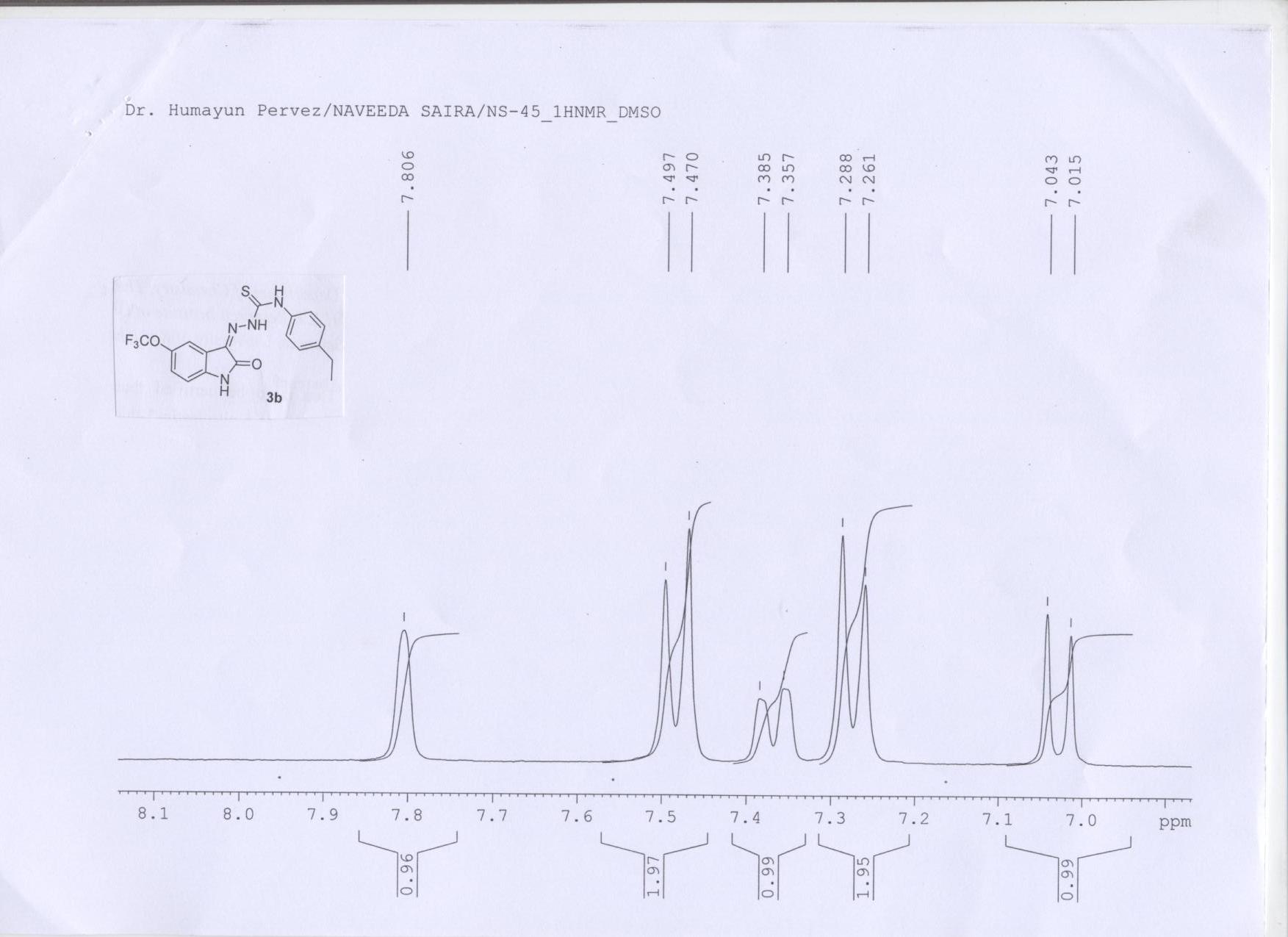

Supplement: Supplementary File 1 [file molecules-16-06408-s001.zip › Spectroscopy/NMR/3b2.jpg]

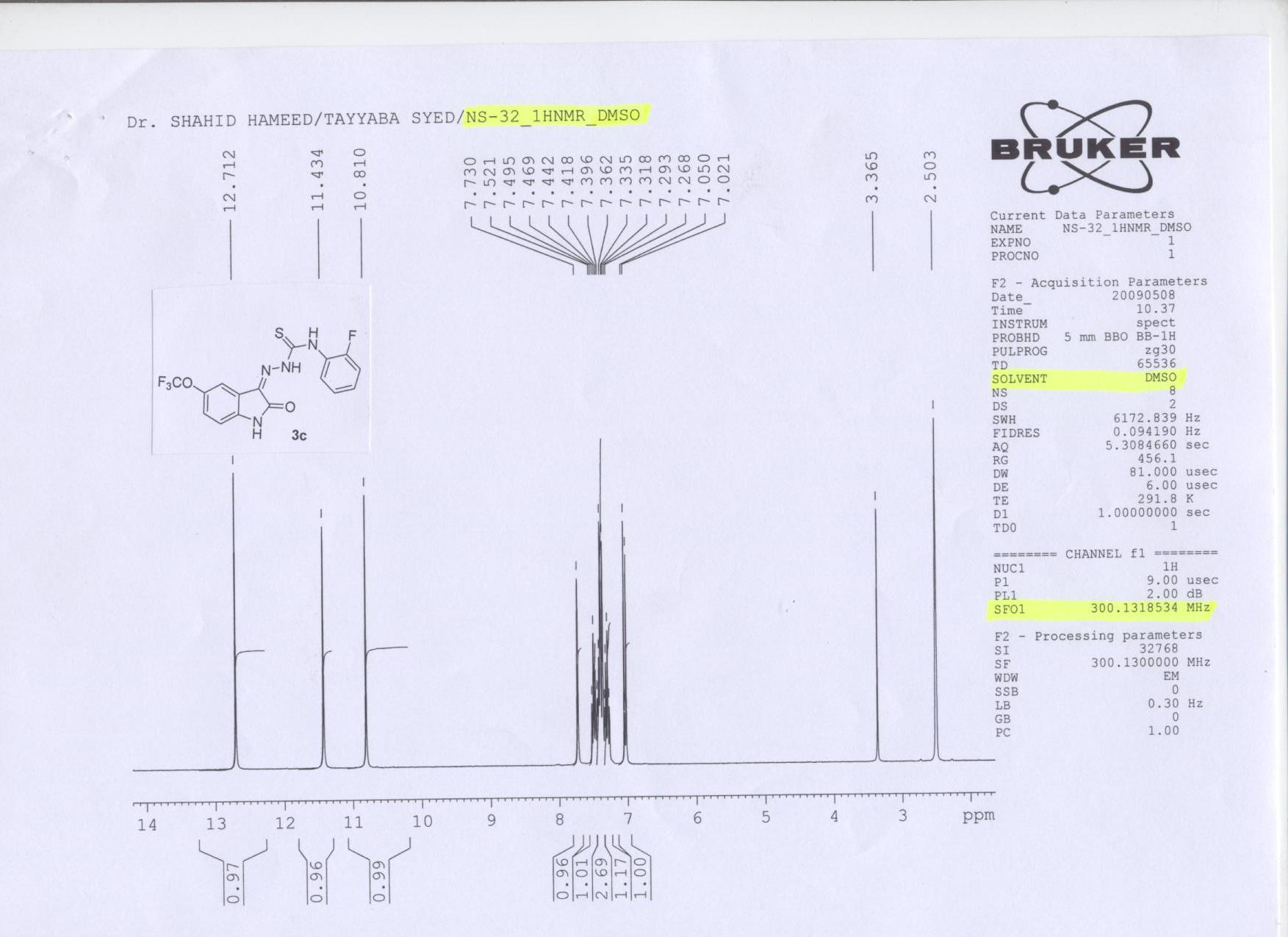

Supplement: Supplementary File 1 [file molecules-16-06408-s001.zip › Spectroscopy/NMR/3c.jpg]

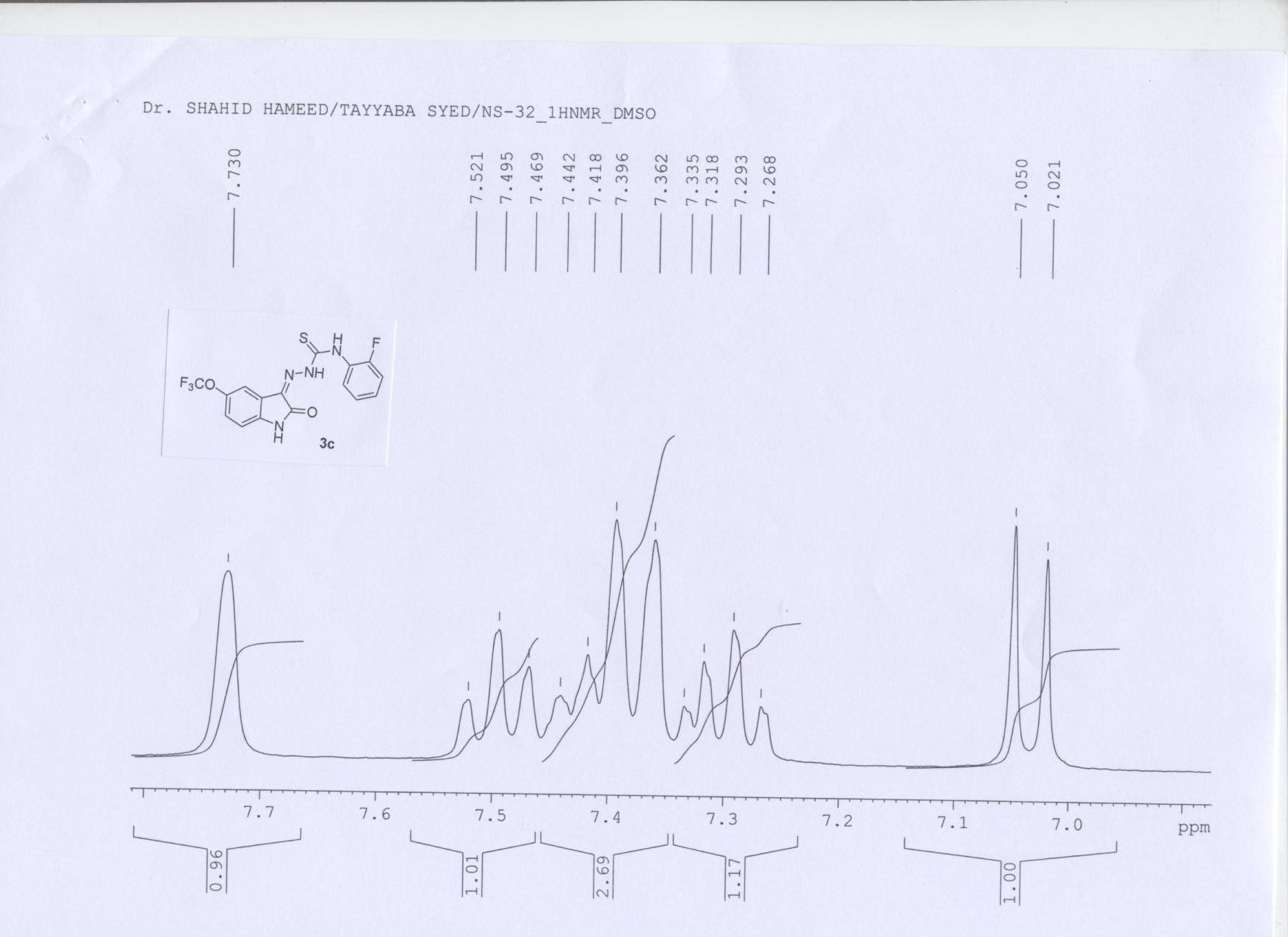

Supplement: Supplementary File 1 [file molecules-16-06408-s001.zip › Spectroscopy/NMR/3c1.jpg]

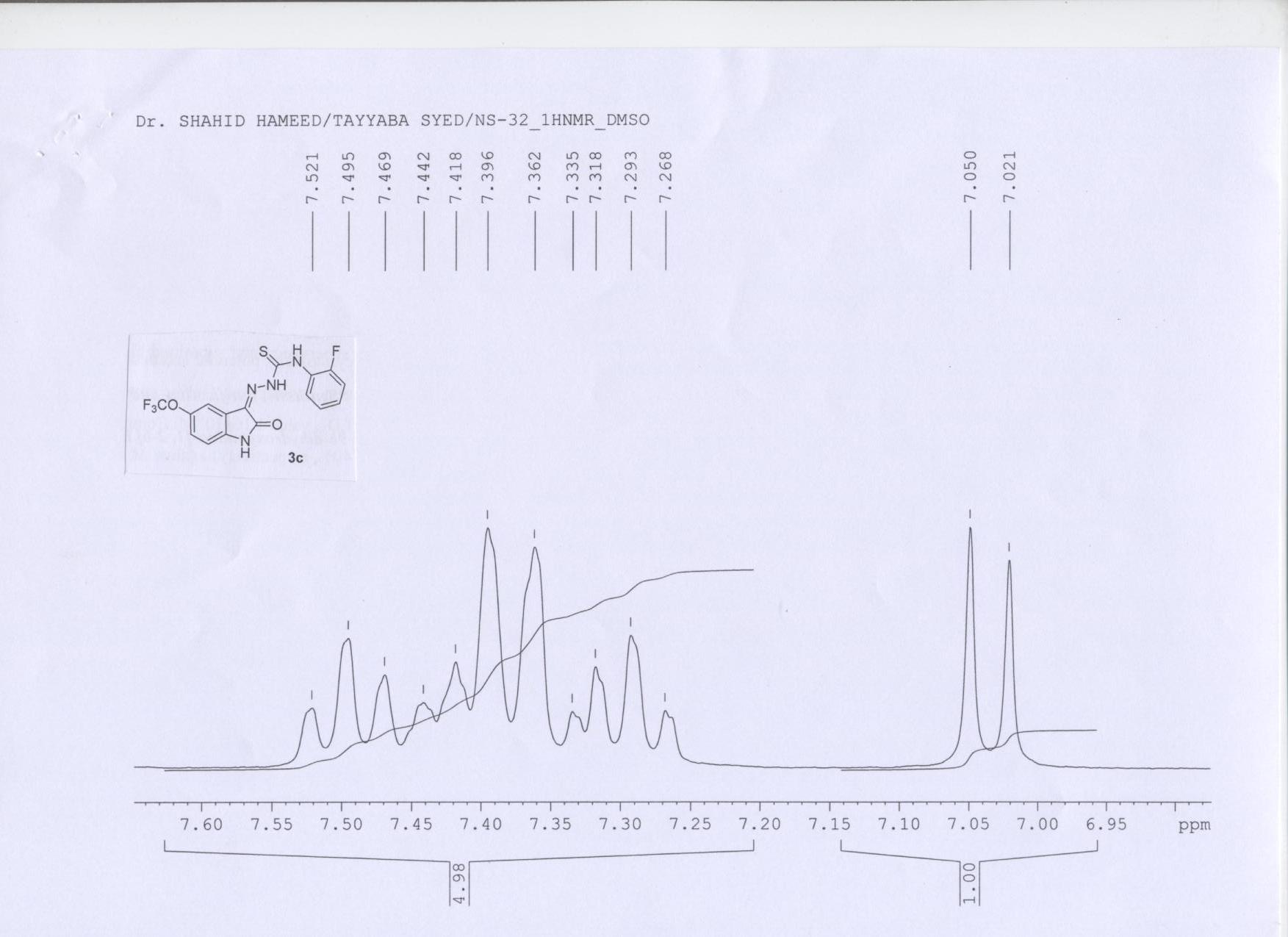

Supplement: Supplementary File 1 [file molecules-16-06408-s001.zip › Spectroscopy/NMR/3c2.jpg]

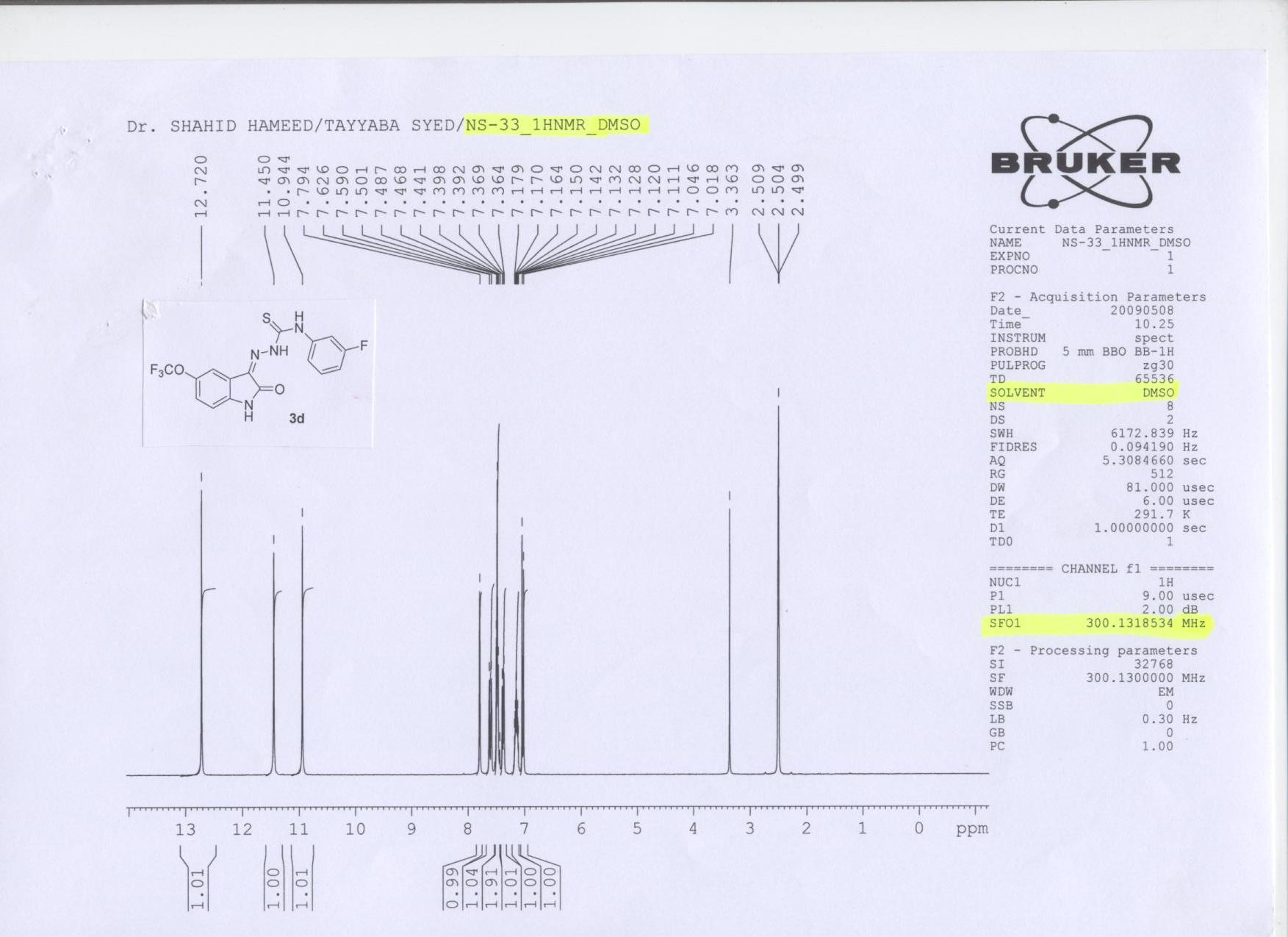

Supplement: Supplementary File 1 [file molecules-16-06408-s001.zip › Spectroscopy/NMR/3d.jpg]

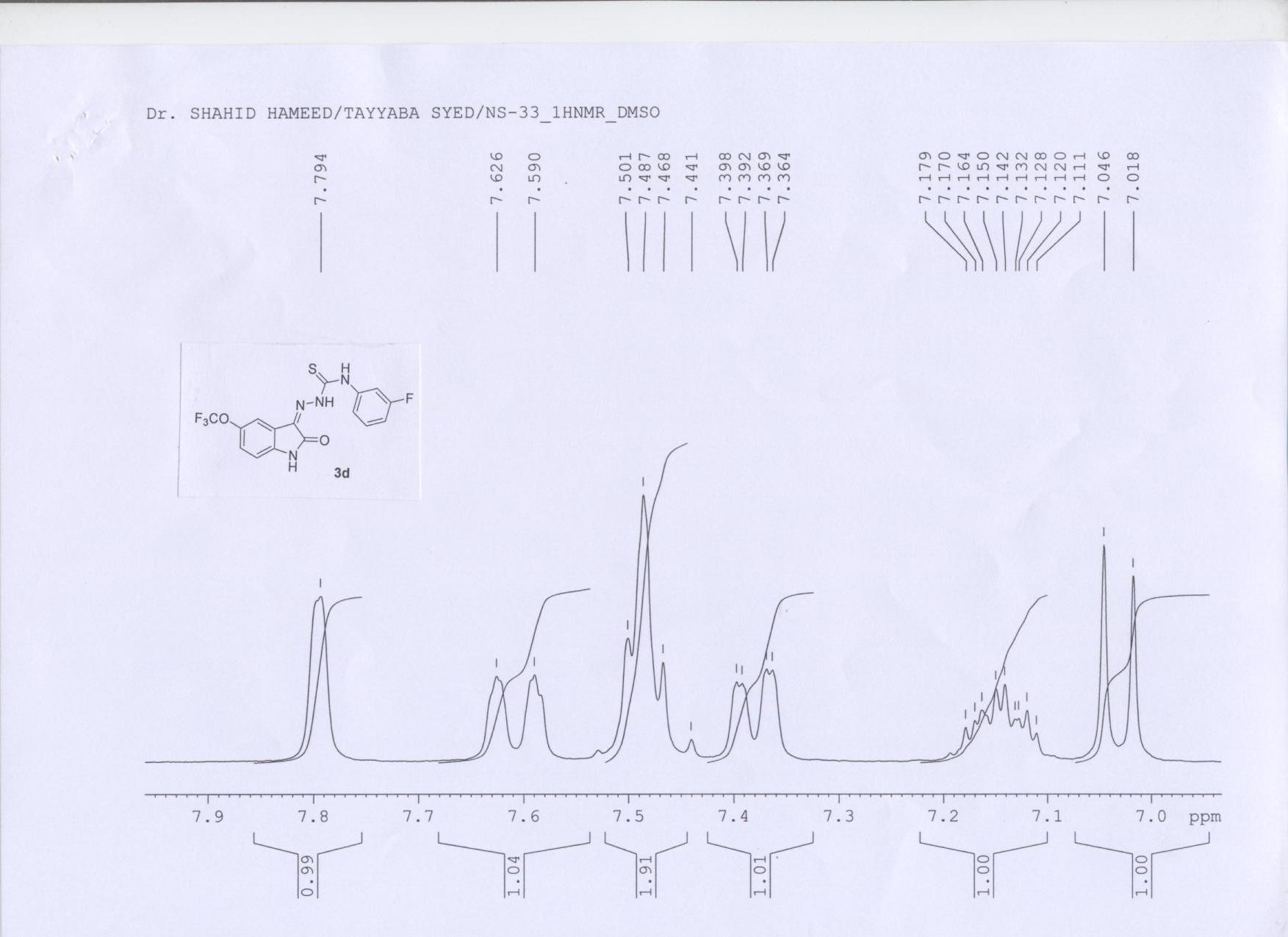

Supplement: Supplementary File 1 [file molecules-16-06408-s001.zip › Spectroscopy/NMR/3d1.jpg]

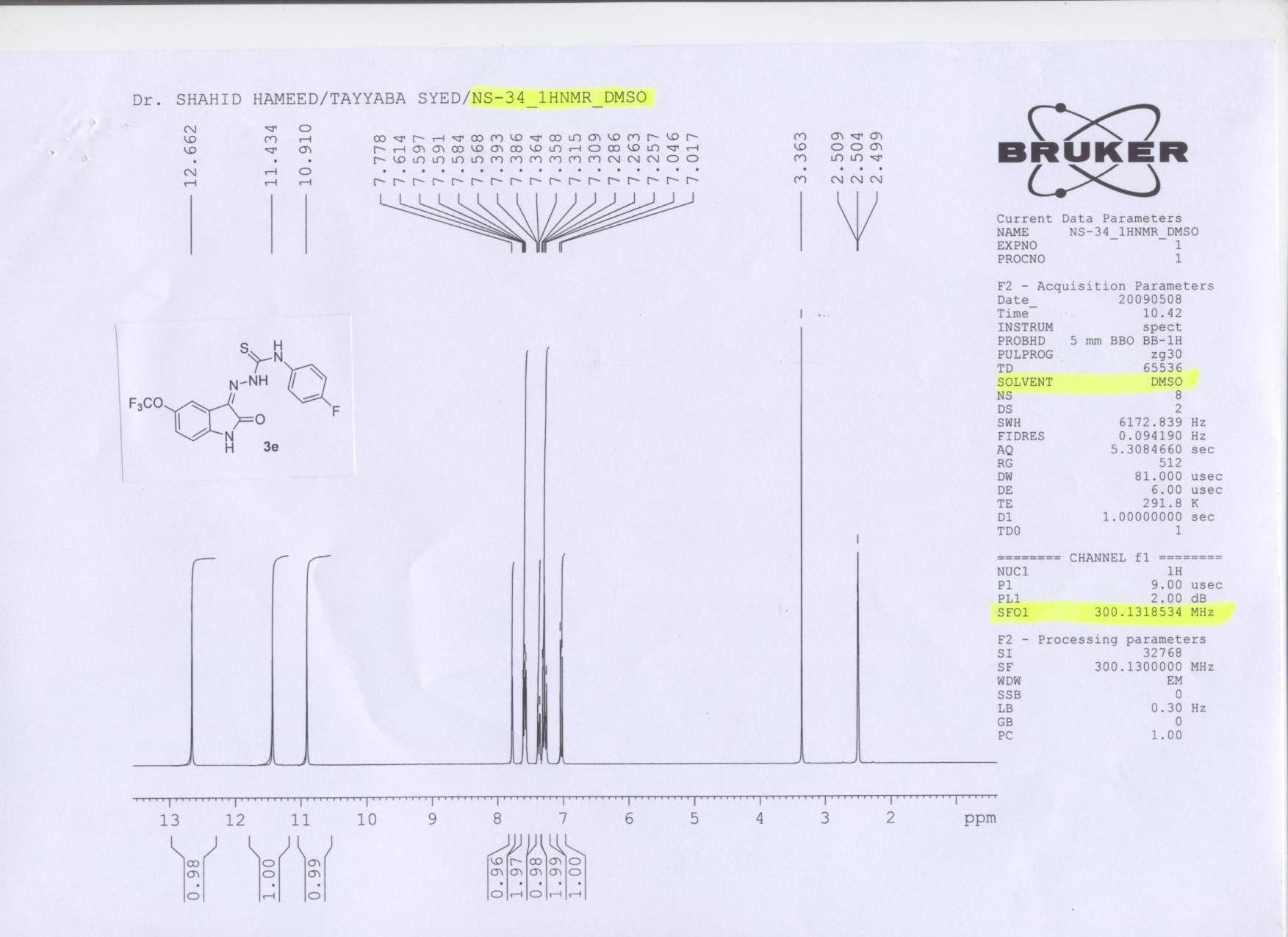

Supplement: Supplementary File 1 [file molecules-16-06408-s001.zip › Spectroscopy/NMR/3e.jpg]

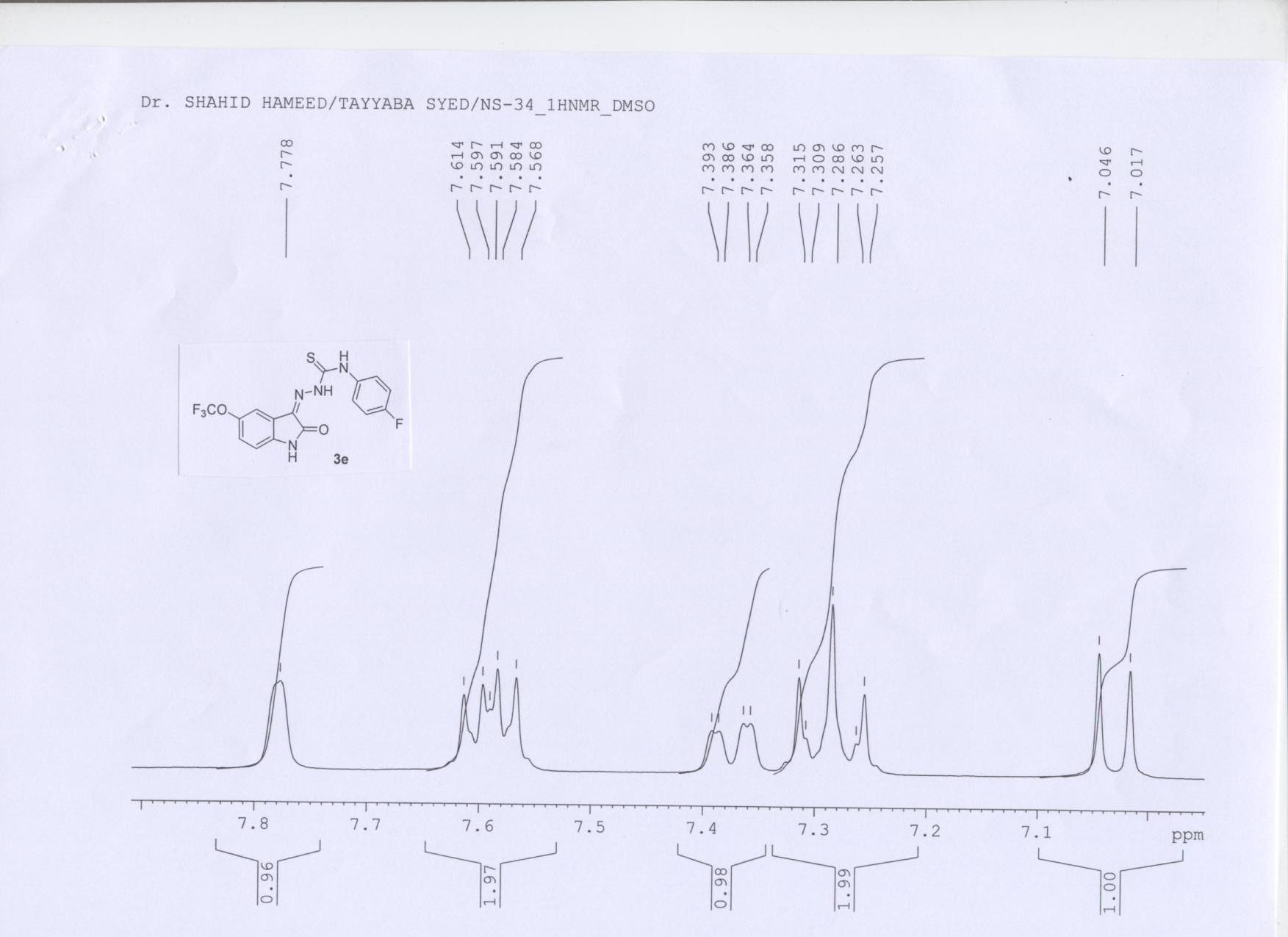

Supplement: Supplementary File 1 [file molecules-16-06408-s001.zip › Spectroscopy/NMR/3e1.jpg]

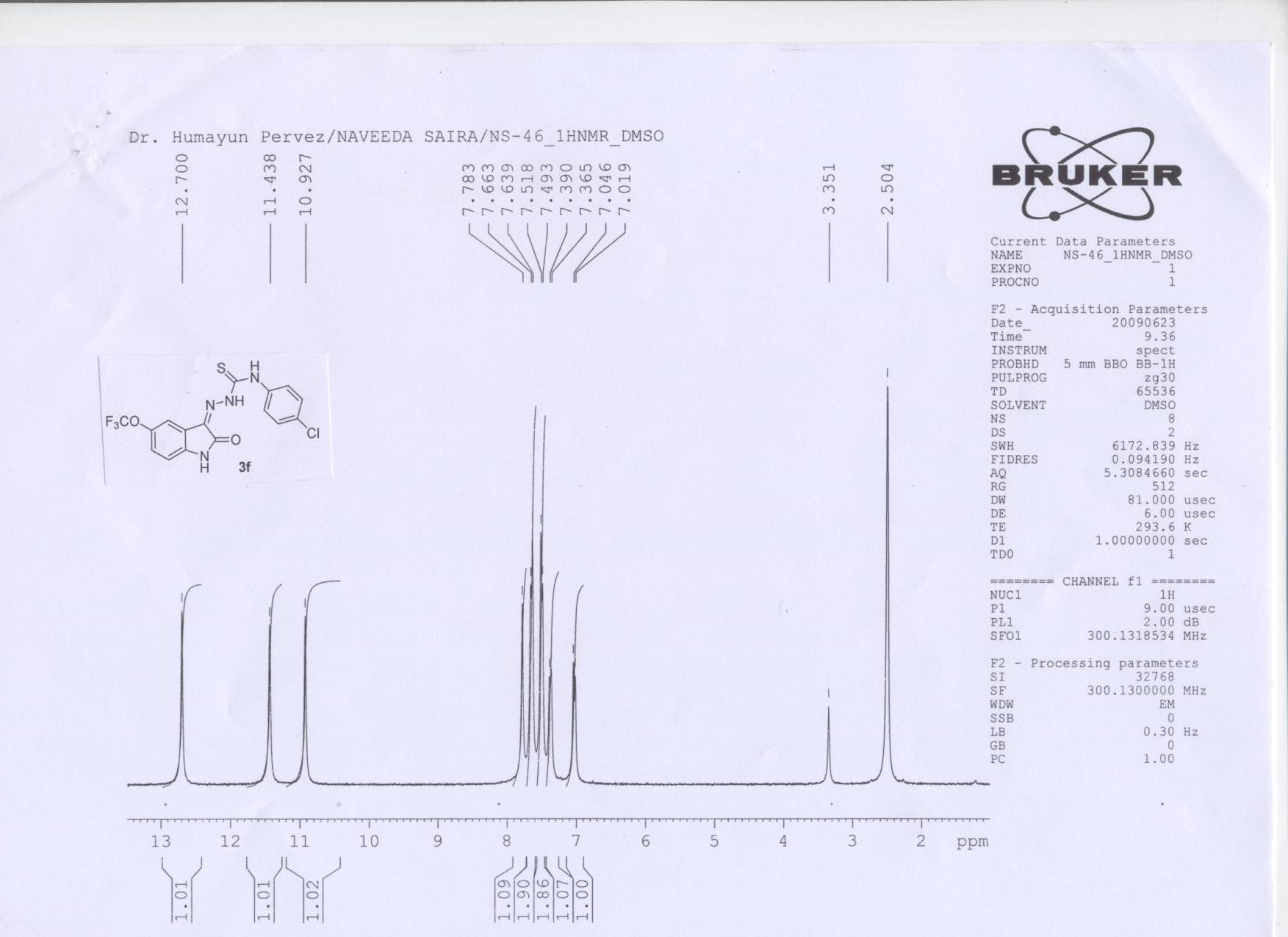

Supplement: Supplementary File 1 [file molecules-16-06408-s001.zip › Spectroscopy/NMR/3f.jpg]

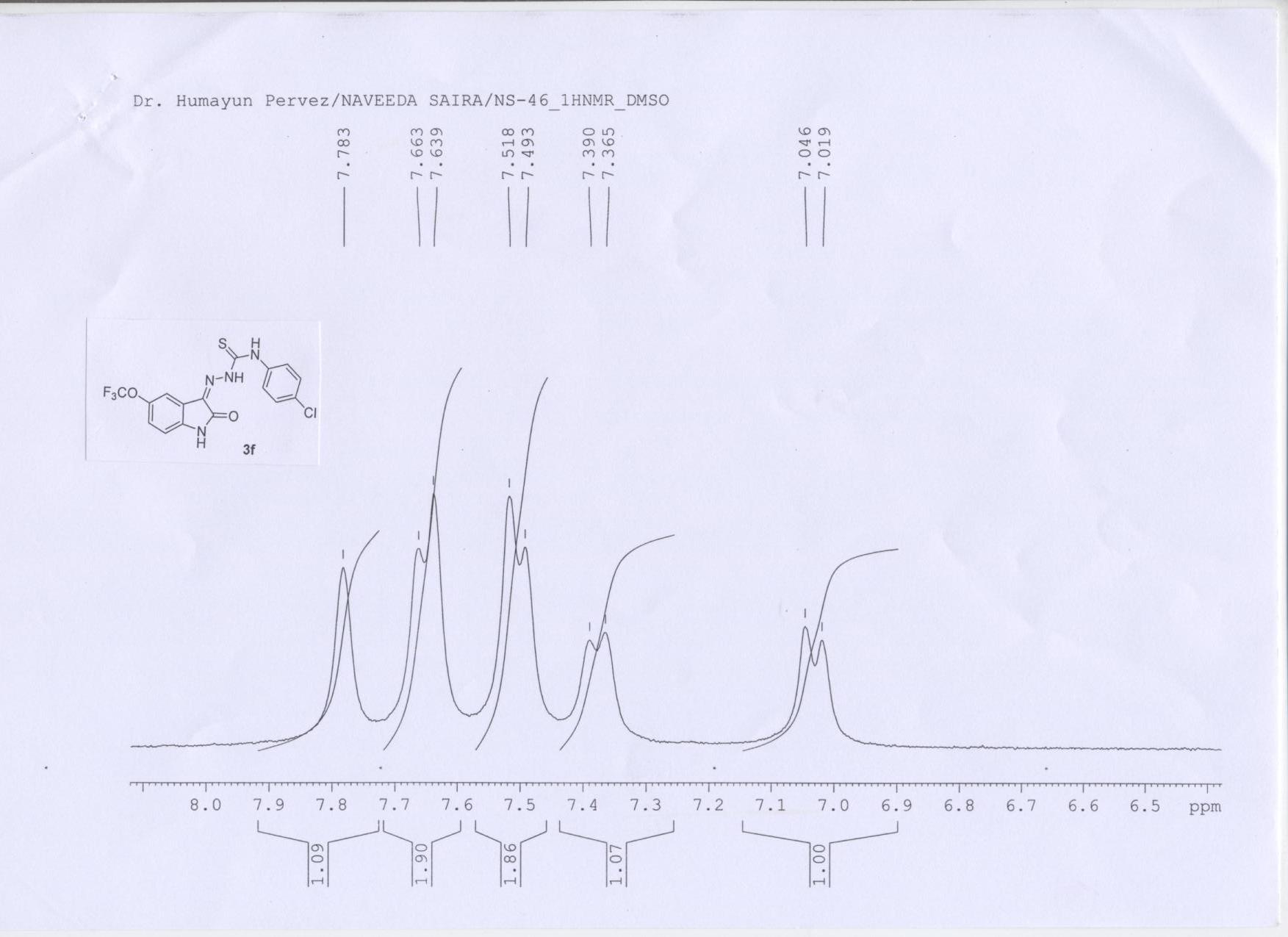

Supplement: Supplementary File 1 [file molecules-16-06408-s001.zip › Spectroscopy/NMR/3f1.jpg]

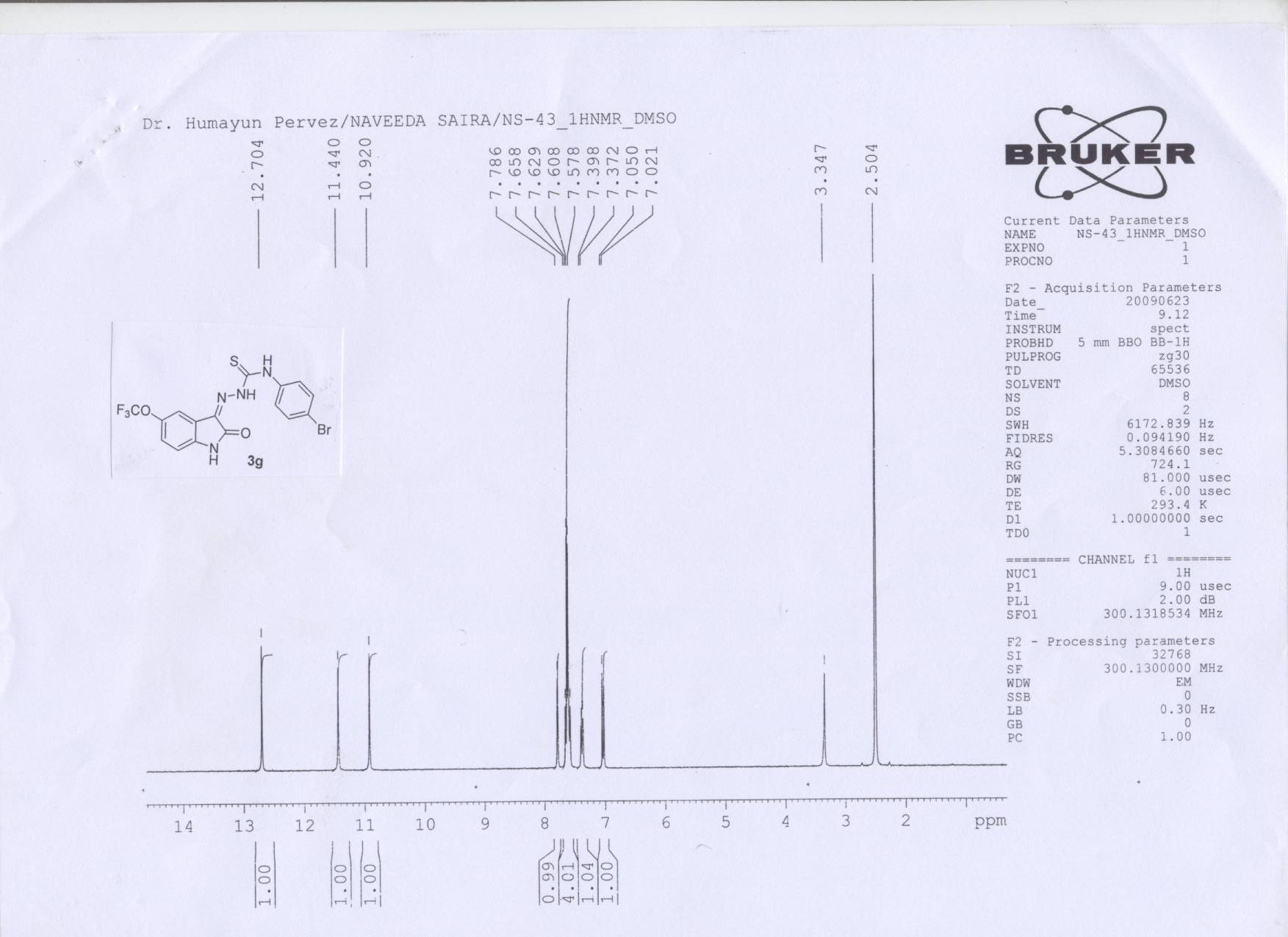

Supplement: Supplementary File 1 [file molecules-16-06408-s001.zip › Spectroscopy/NMR/3g.jpg]

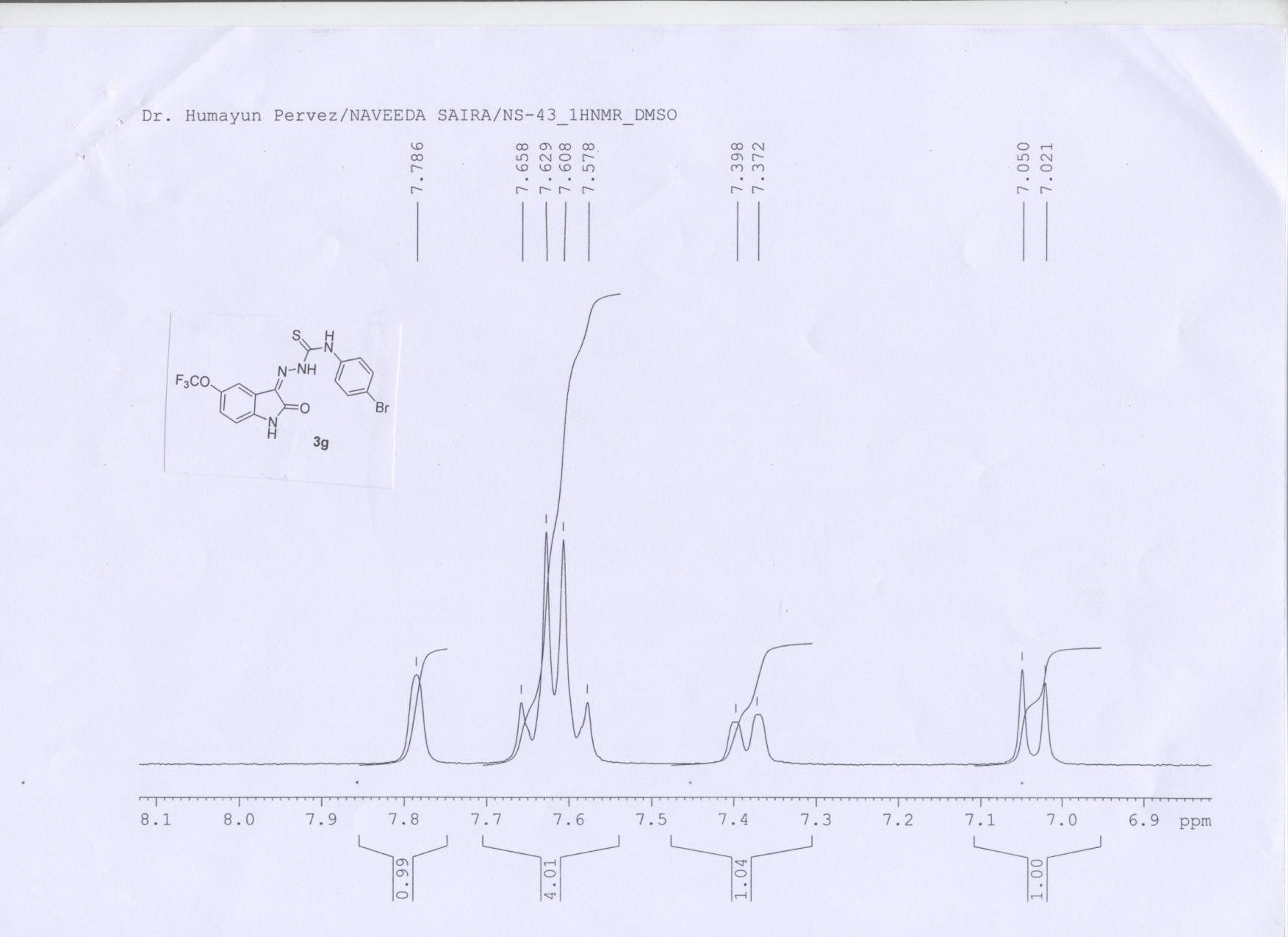

Supplement: Supplementary File 1 [file molecules-16-06408-s001.zip › Spectroscopy/NMR/3g1.jpg]

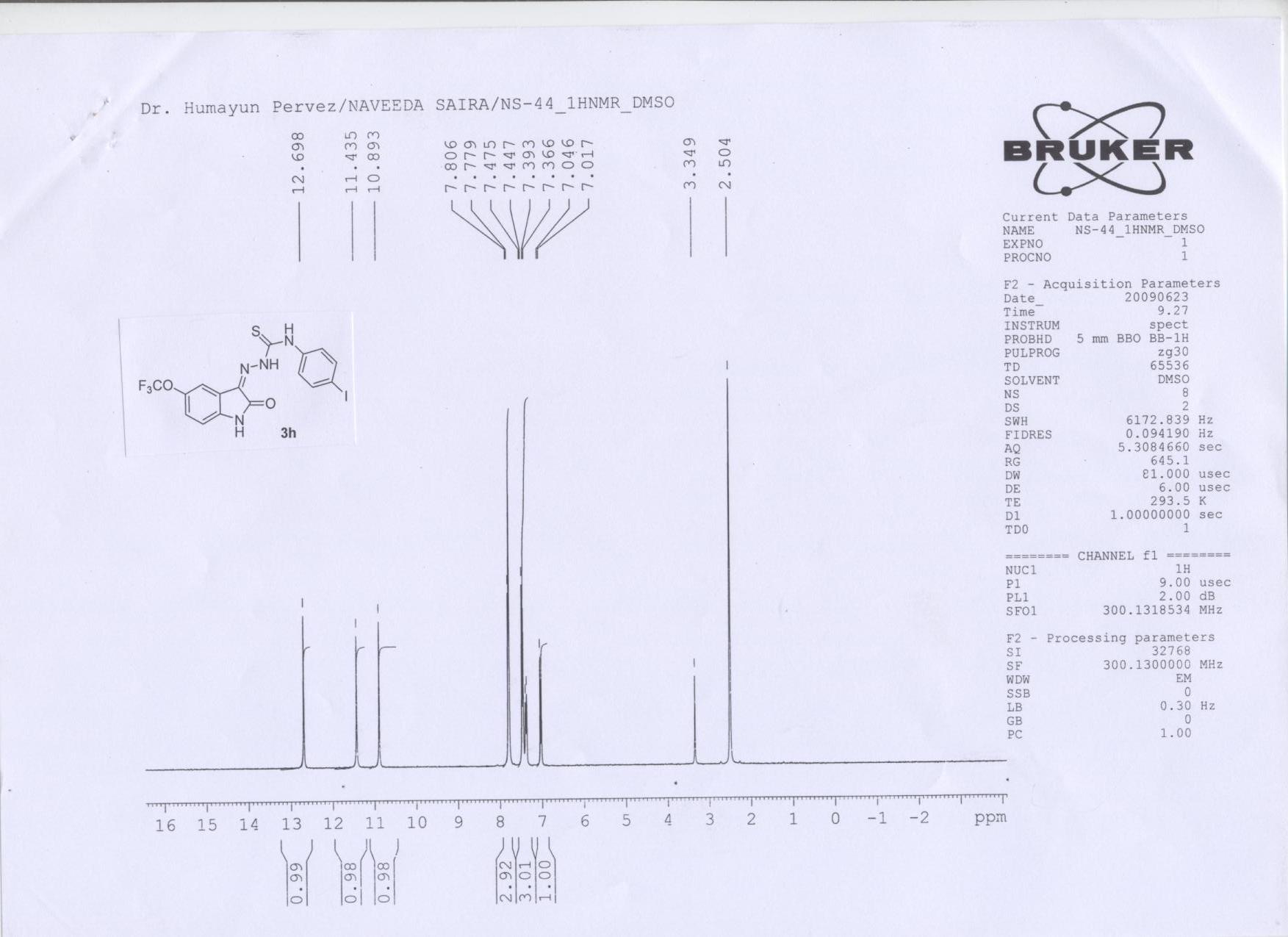

Supplement: Supplementary File 1 [file molecules-16-06408-s001.zip › Spectroscopy/NMR/3h.jpg]

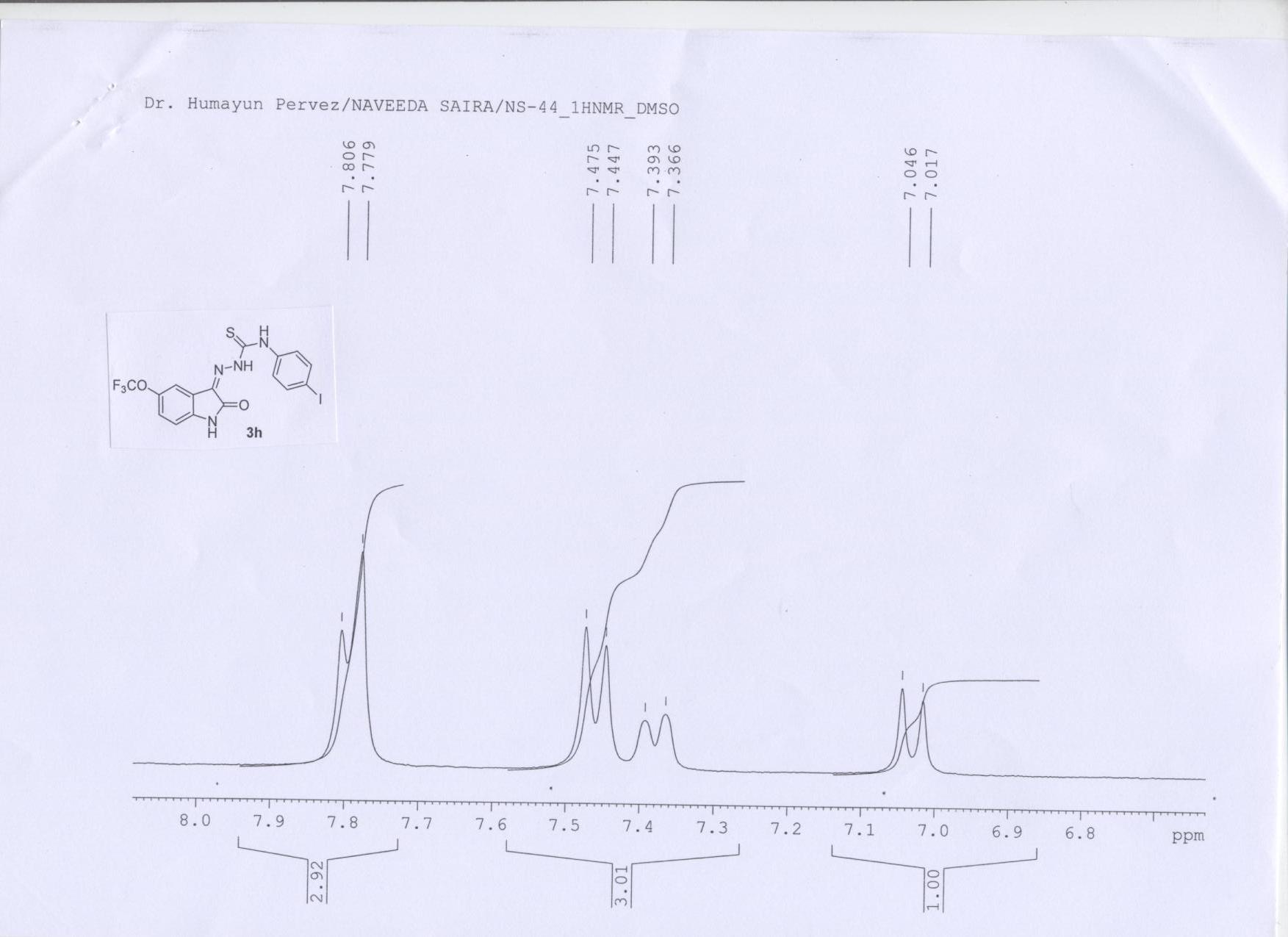

Supplement: Supplementary File 1 [file molecules-16-06408-s001.zip › Spectroscopy/NMR/3h1.jpg]

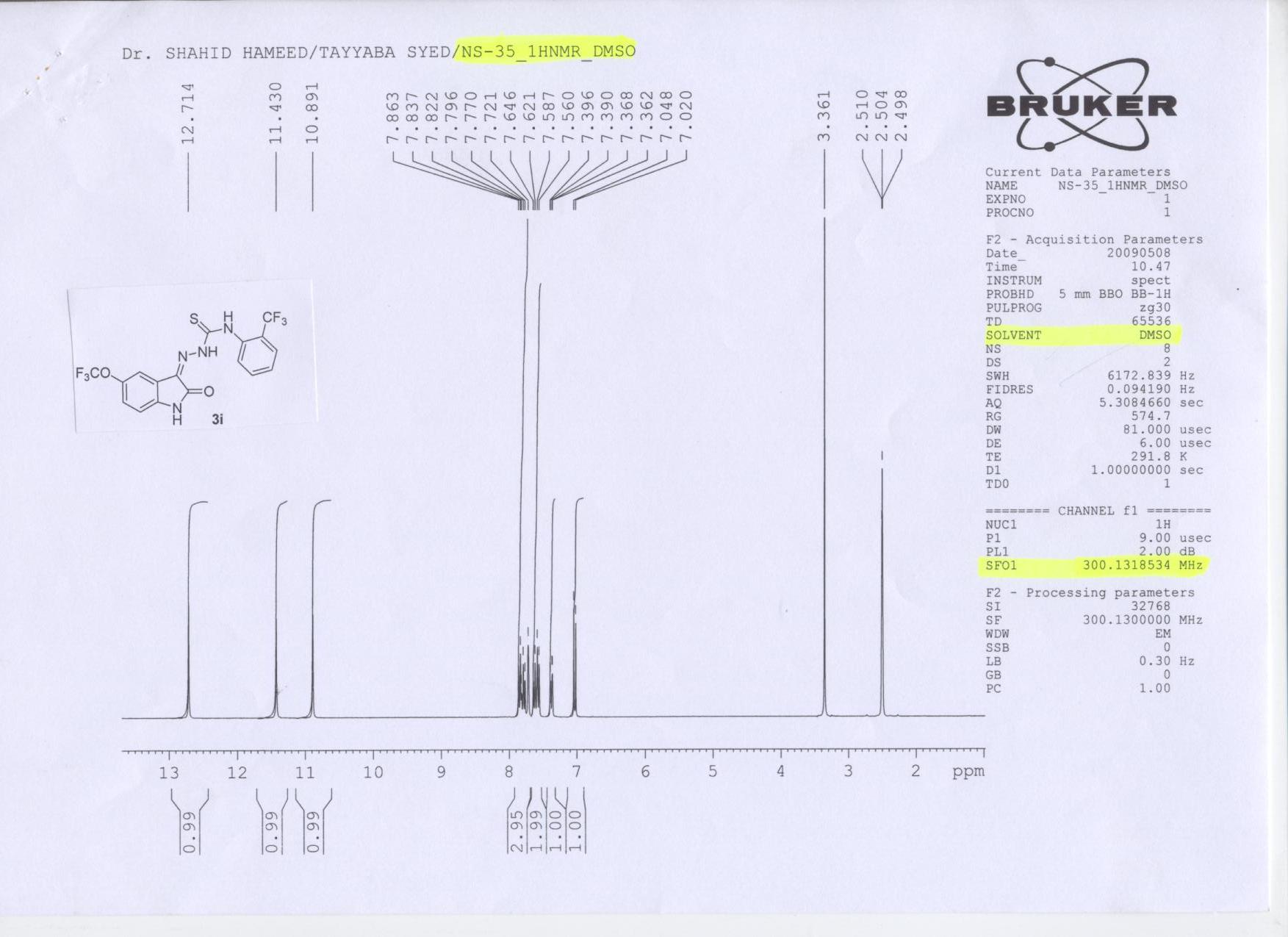

Supplement: Supplementary File 1 [file molecules-16-06408-s001.zip › Spectroscopy/NMR/3i.jpg]

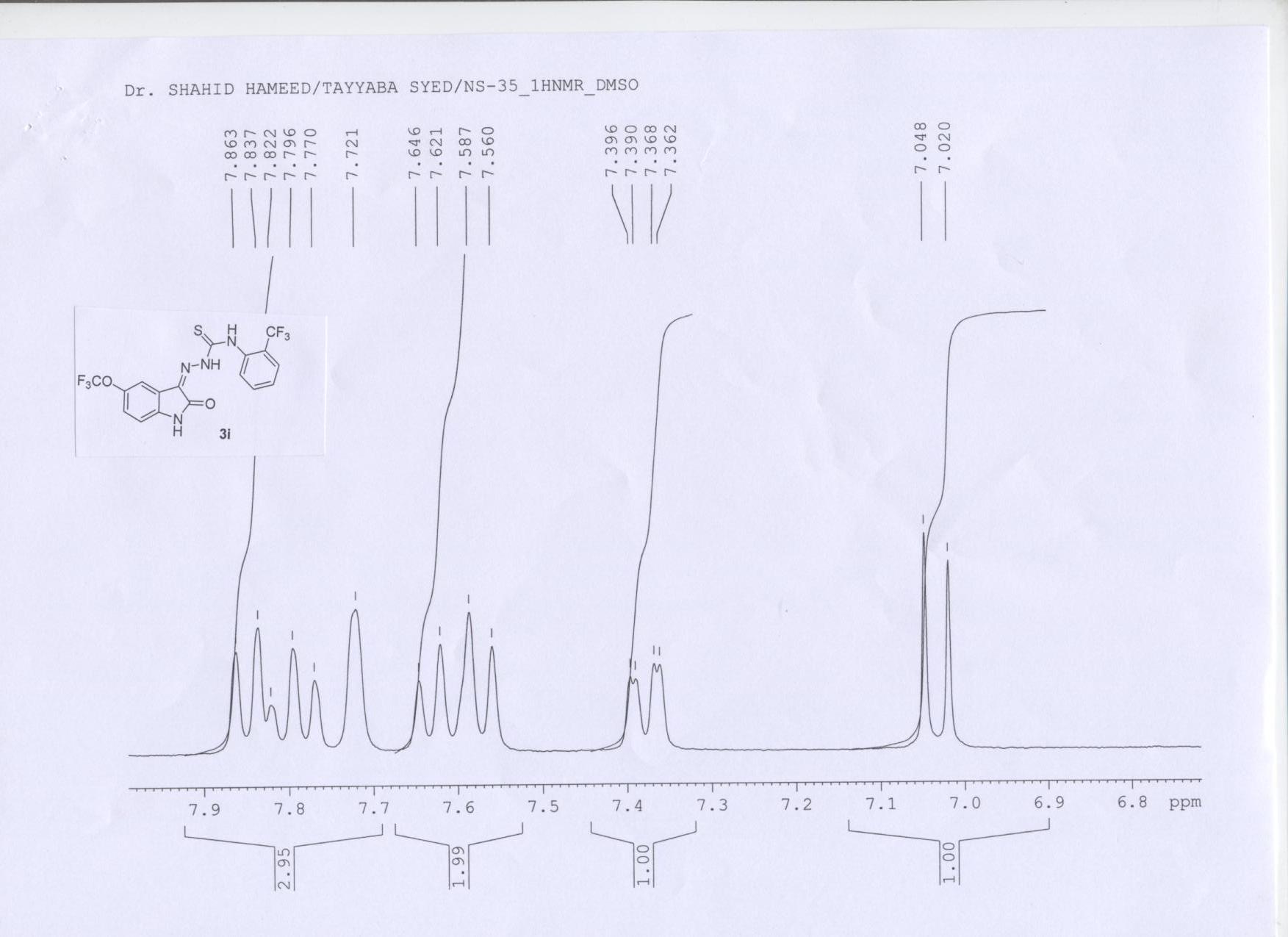

Supplement: Supplementary File 1 [file molecules-16-06408-s001.zip › Spectroscopy/NMR/3i1.jpg]

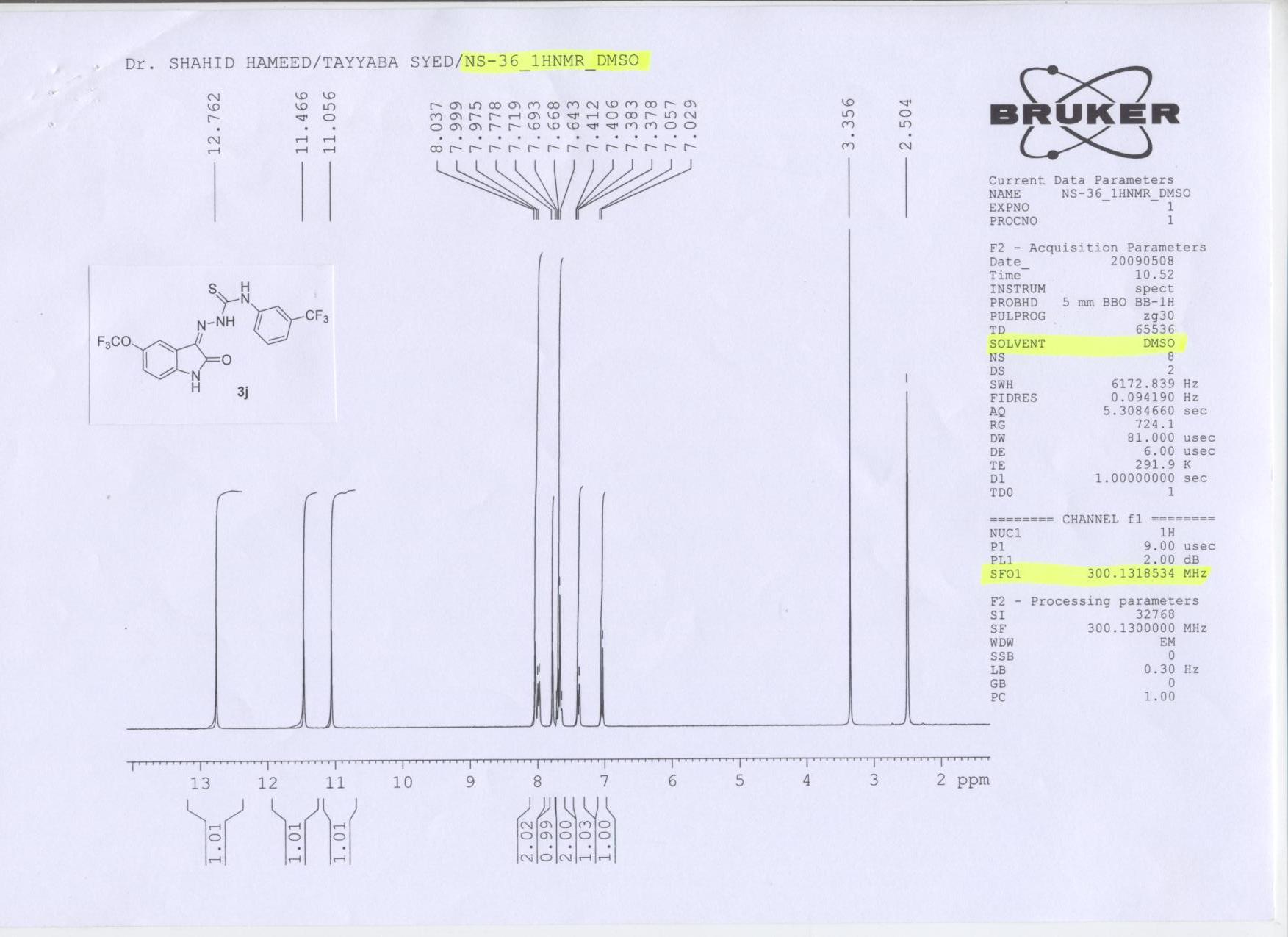

Supplement: Supplementary File 1 [file molecules-16-06408-s001.zip › Spectroscopy/NMR/3j.jpg]

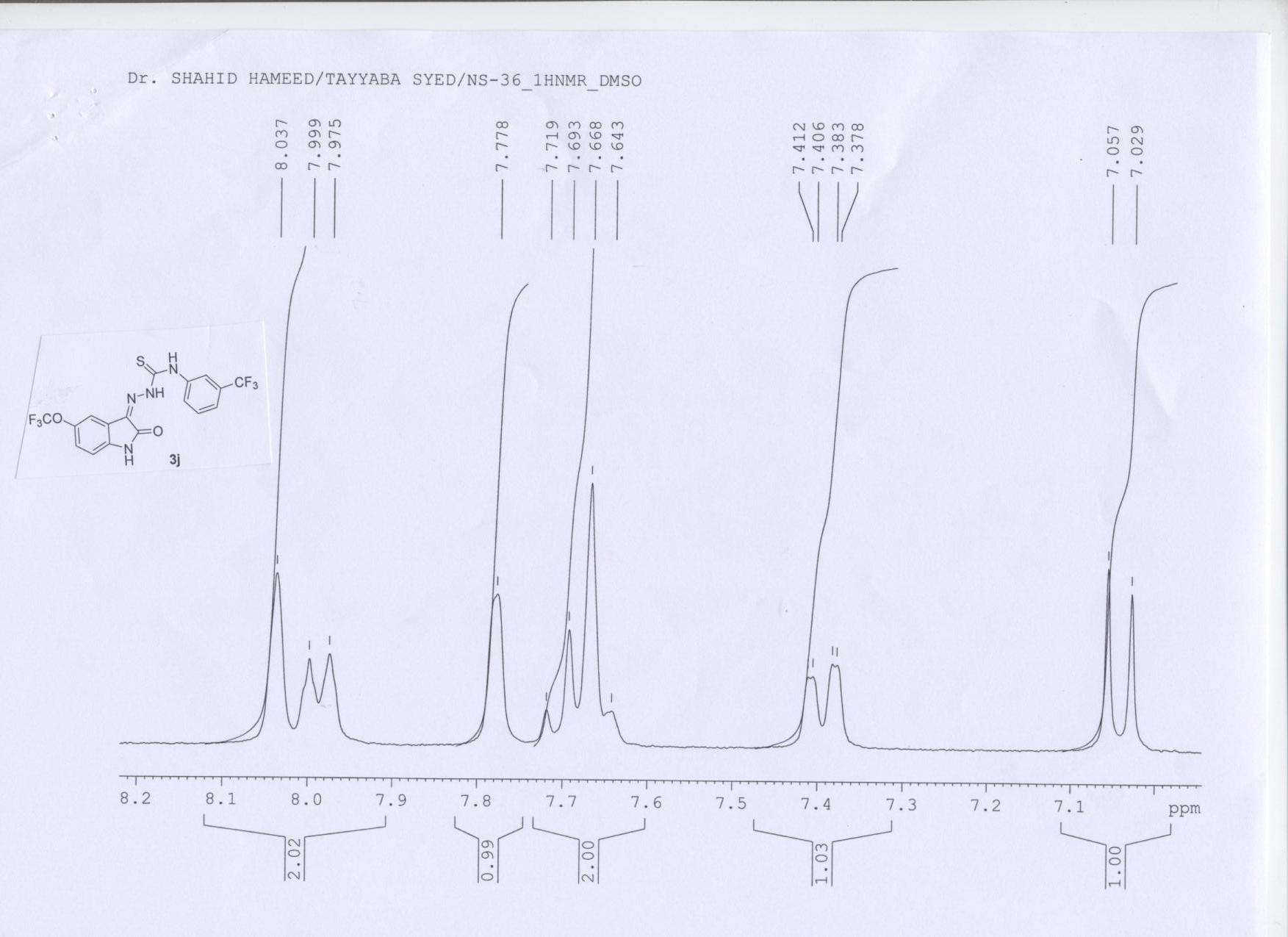

Supplement: Supplementary File 1 [file molecules-16-06408-s001.zip › Spectroscopy/NMR/3j1.jpg]

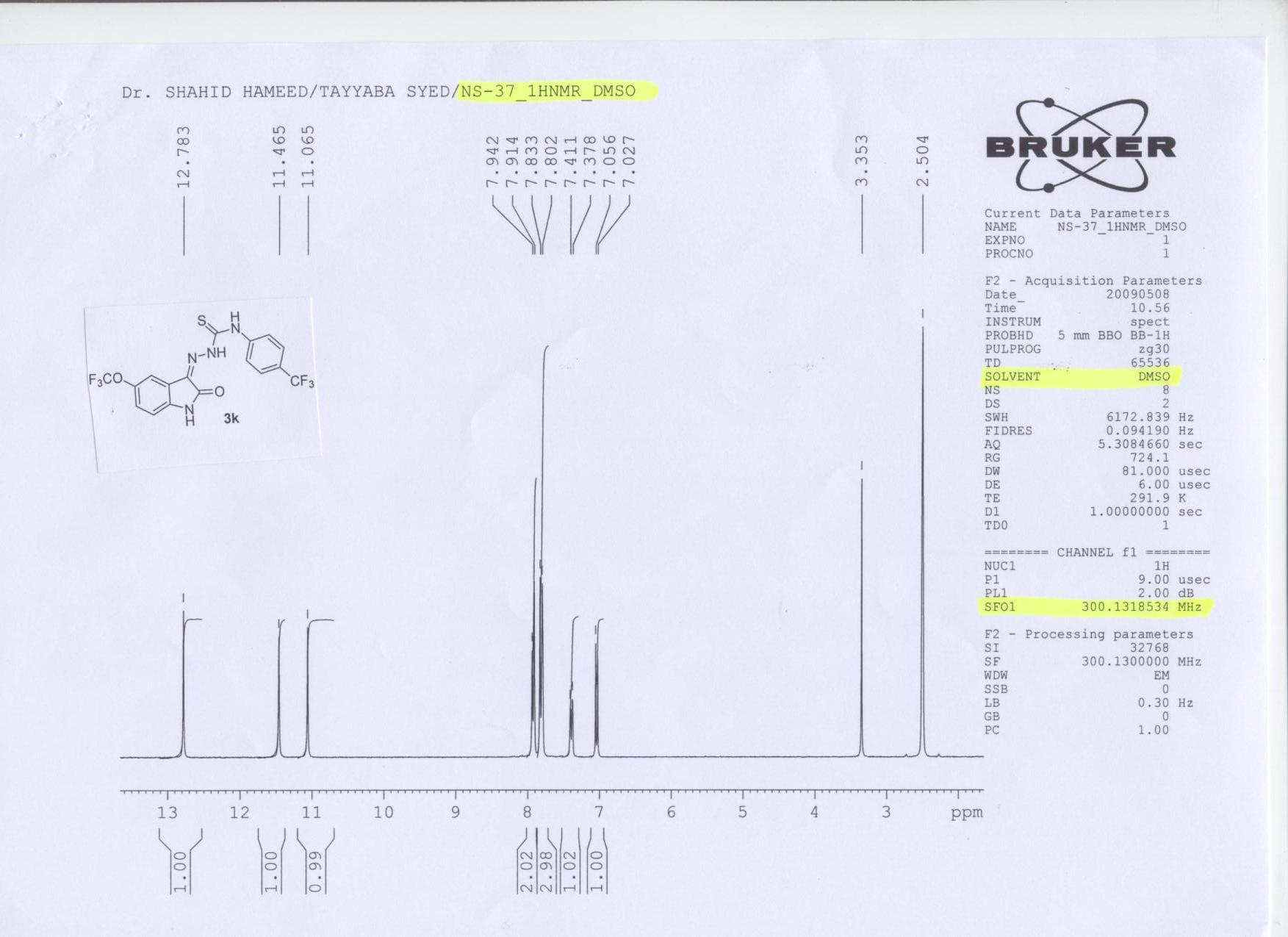

Supplement: Supplementary File 1 [file molecules-16-06408-s001.zip › Spectroscopy/NMR/3k.jpg]

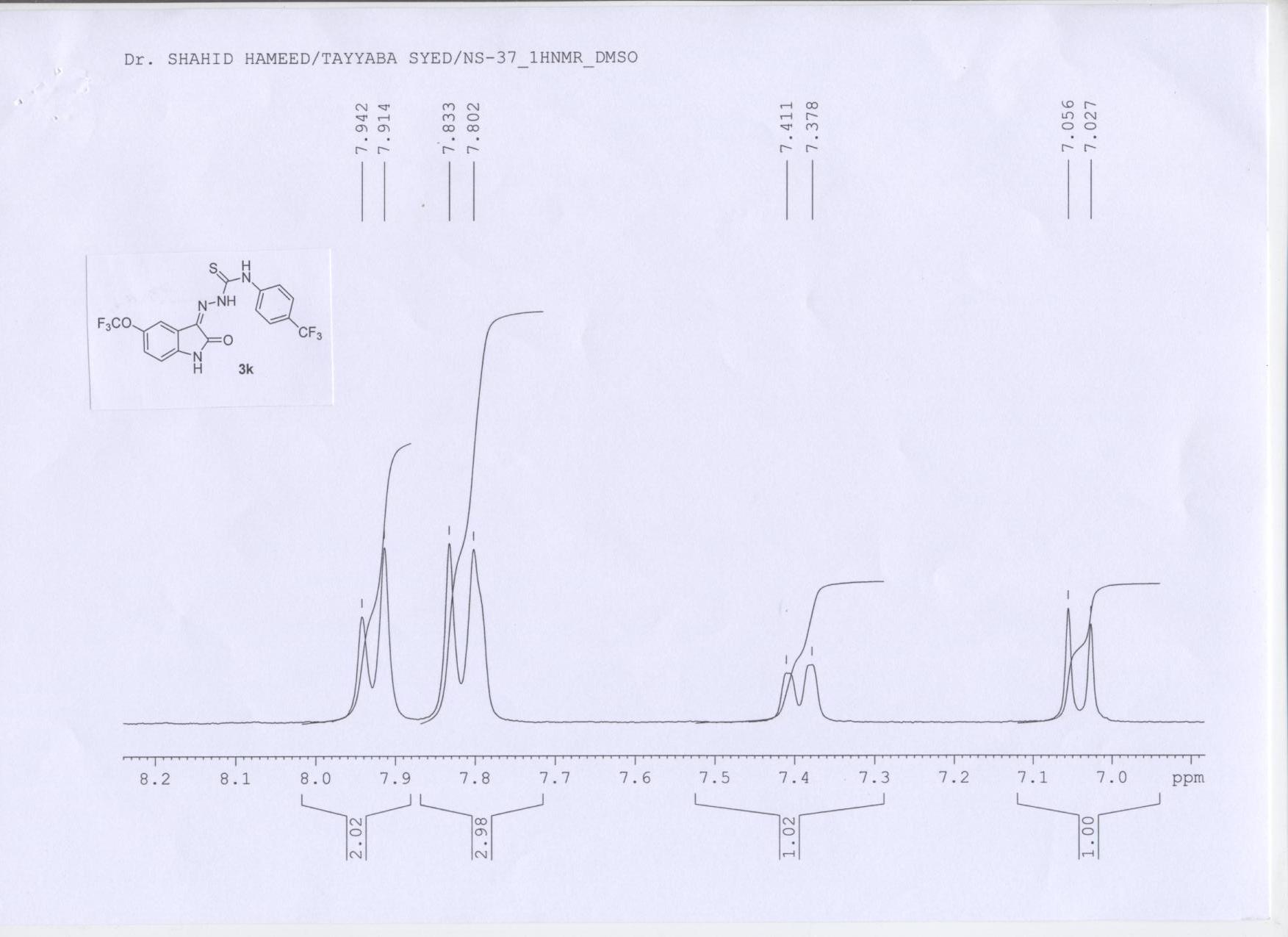

Supplement: Supplementary File 1 [file molecules-16-06408-s001.zip › Spectroscopy/NMR/3k1.jpg]

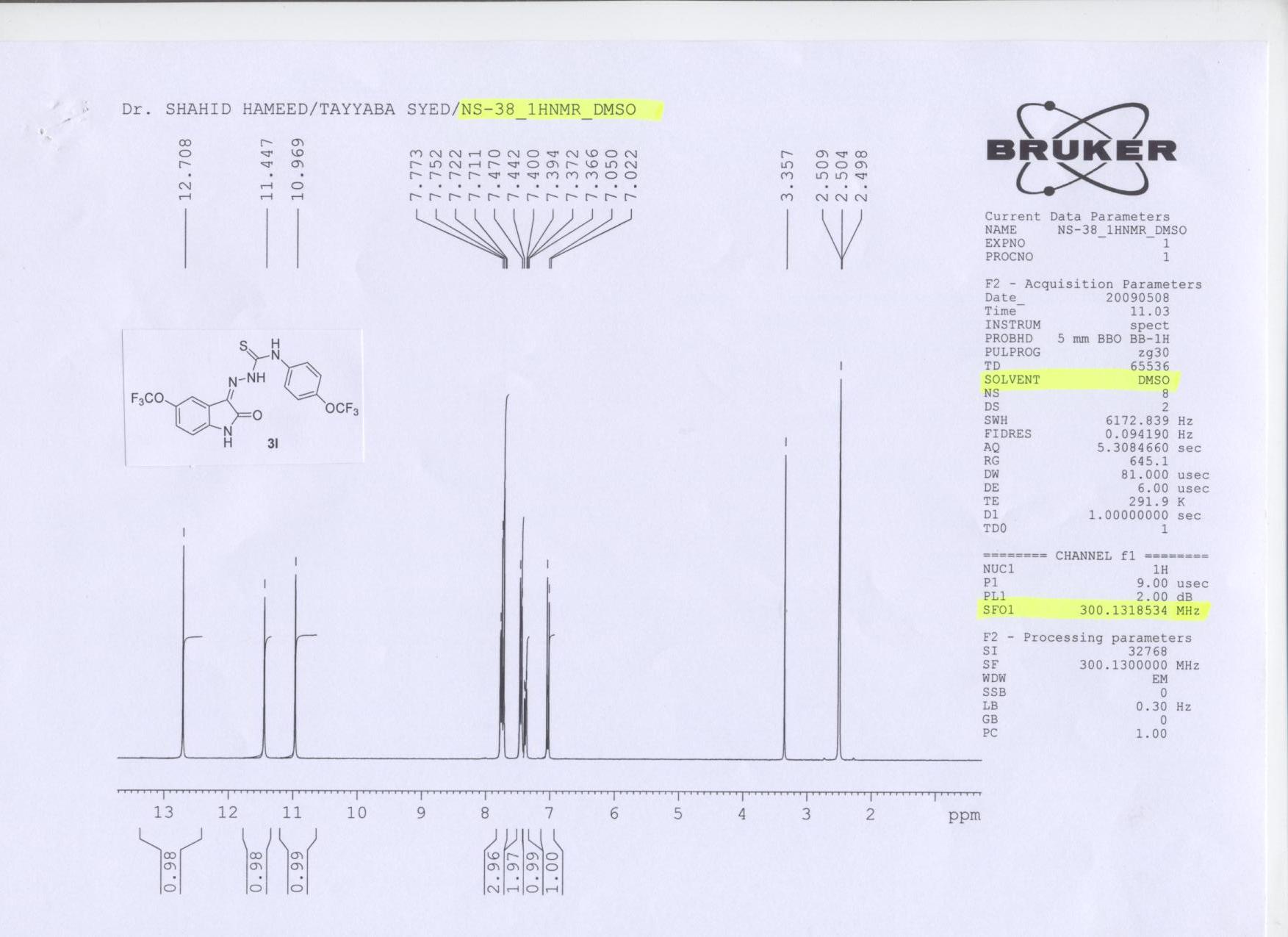

Supplement: Supplementary File 1 [file molecules-16-06408-s001.zip › Spectroscopy/NMR/3l.jpg]

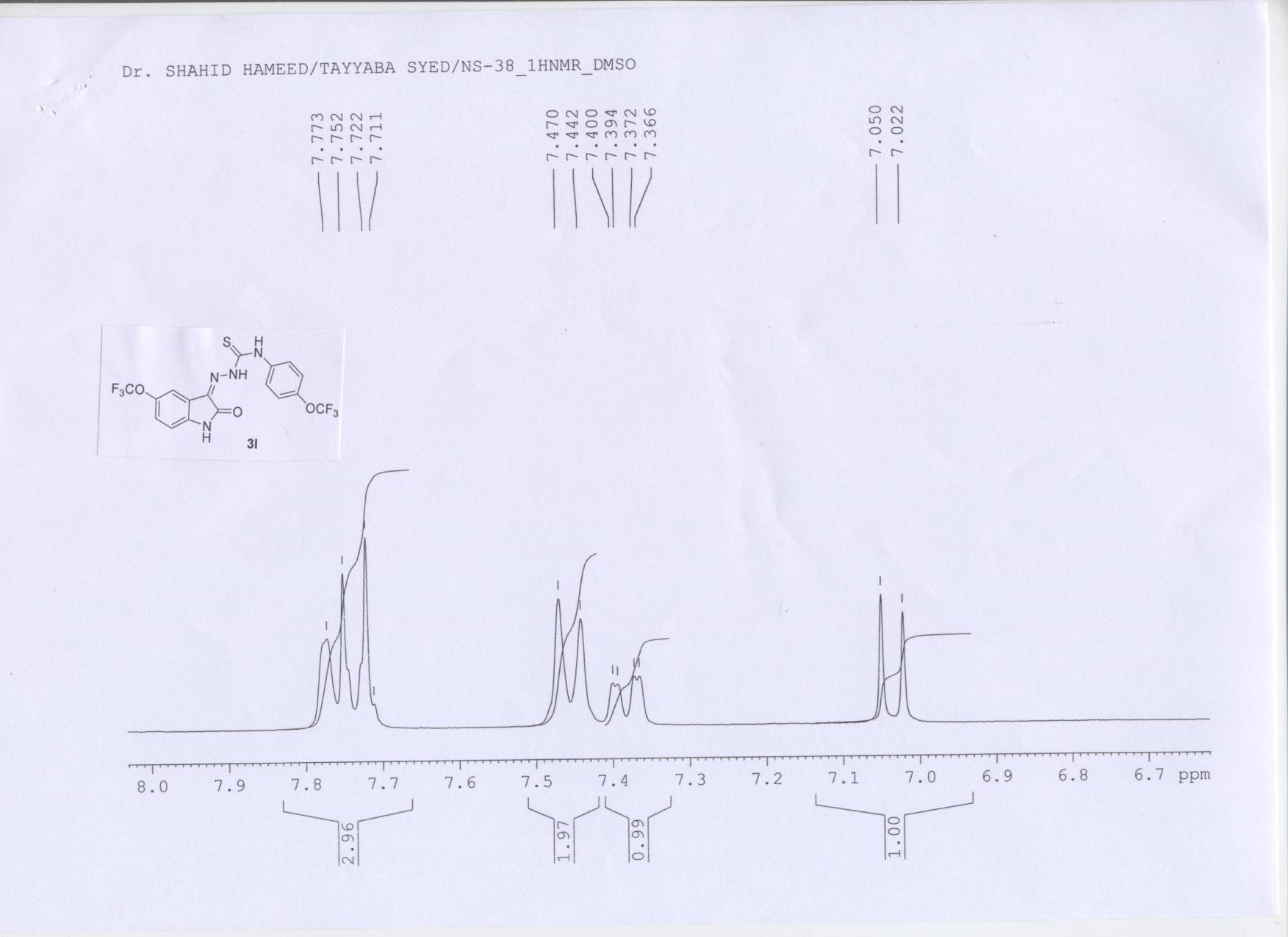

Supplement: Supplementary File 1 [file molecules-16-06408-s001.zip › Spectroscopy/NMR/3l1.jpg]

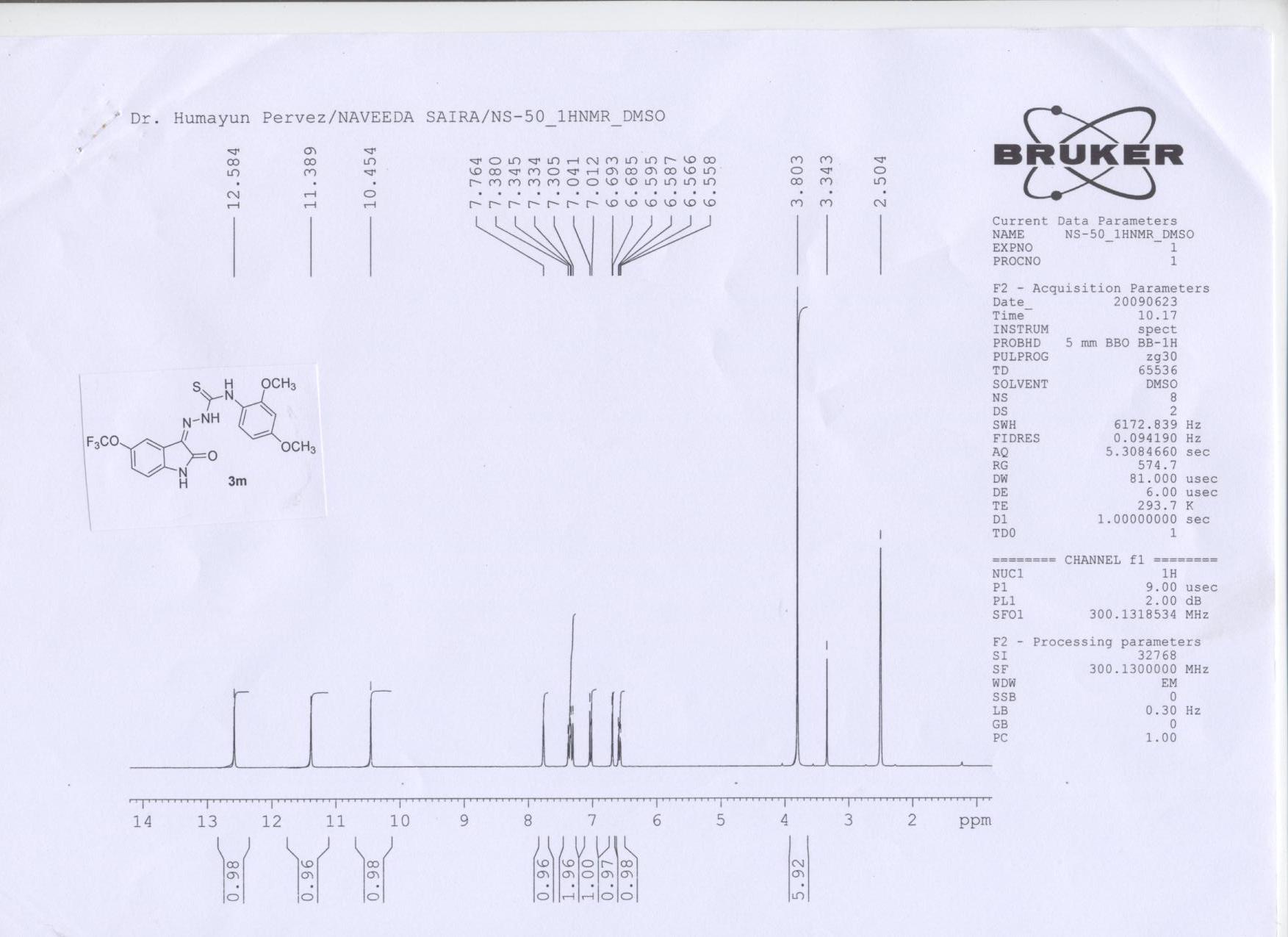

Supplement: Supplementary File 1 [file molecules-16-06408-s001.zip › Spectroscopy/NMR/3m.jpg]

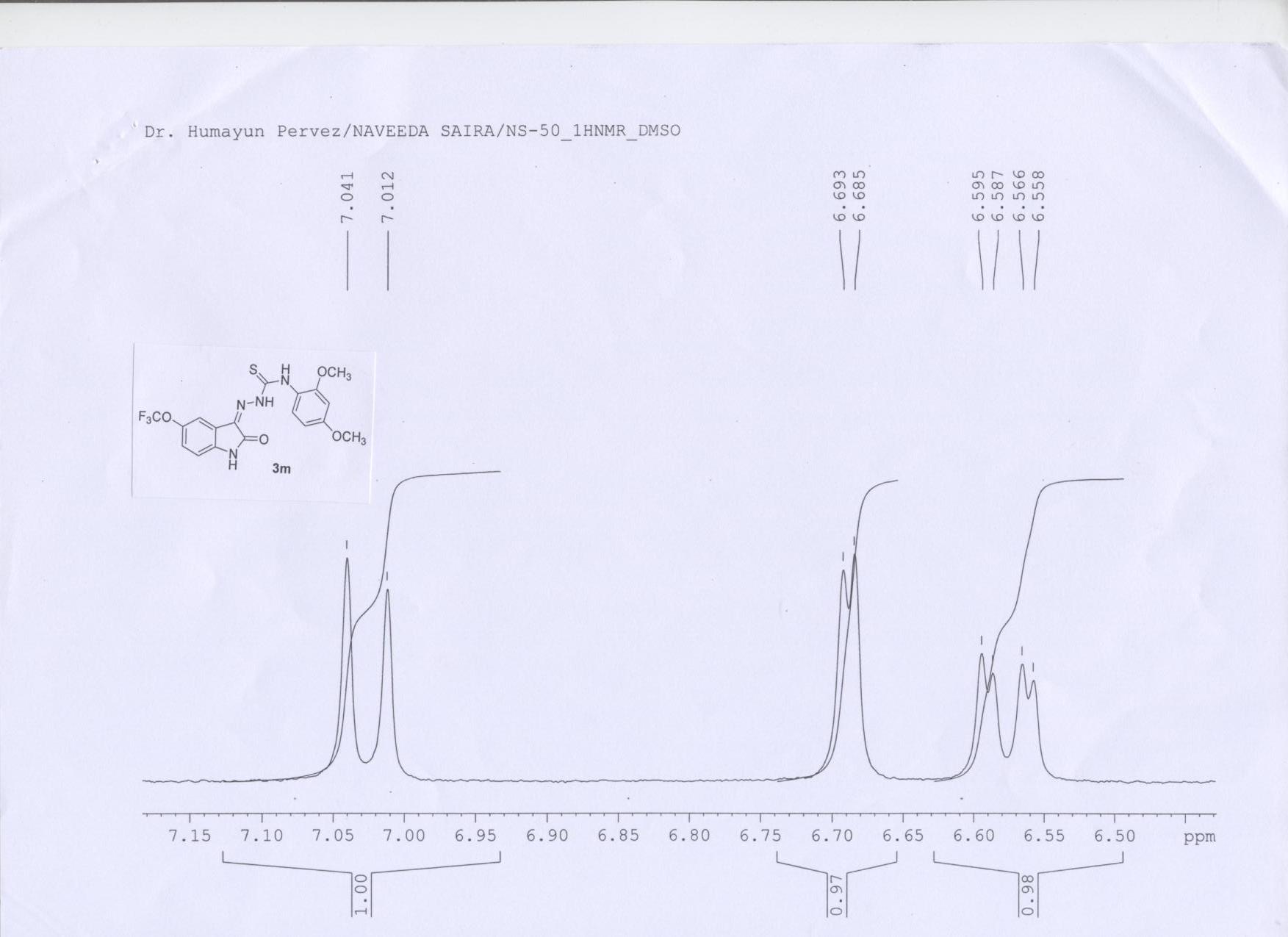

Supplement: Supplementary File 1 [file molecules-16-06408-s001.zip › Spectroscopy/NMR/3m1.jpg]

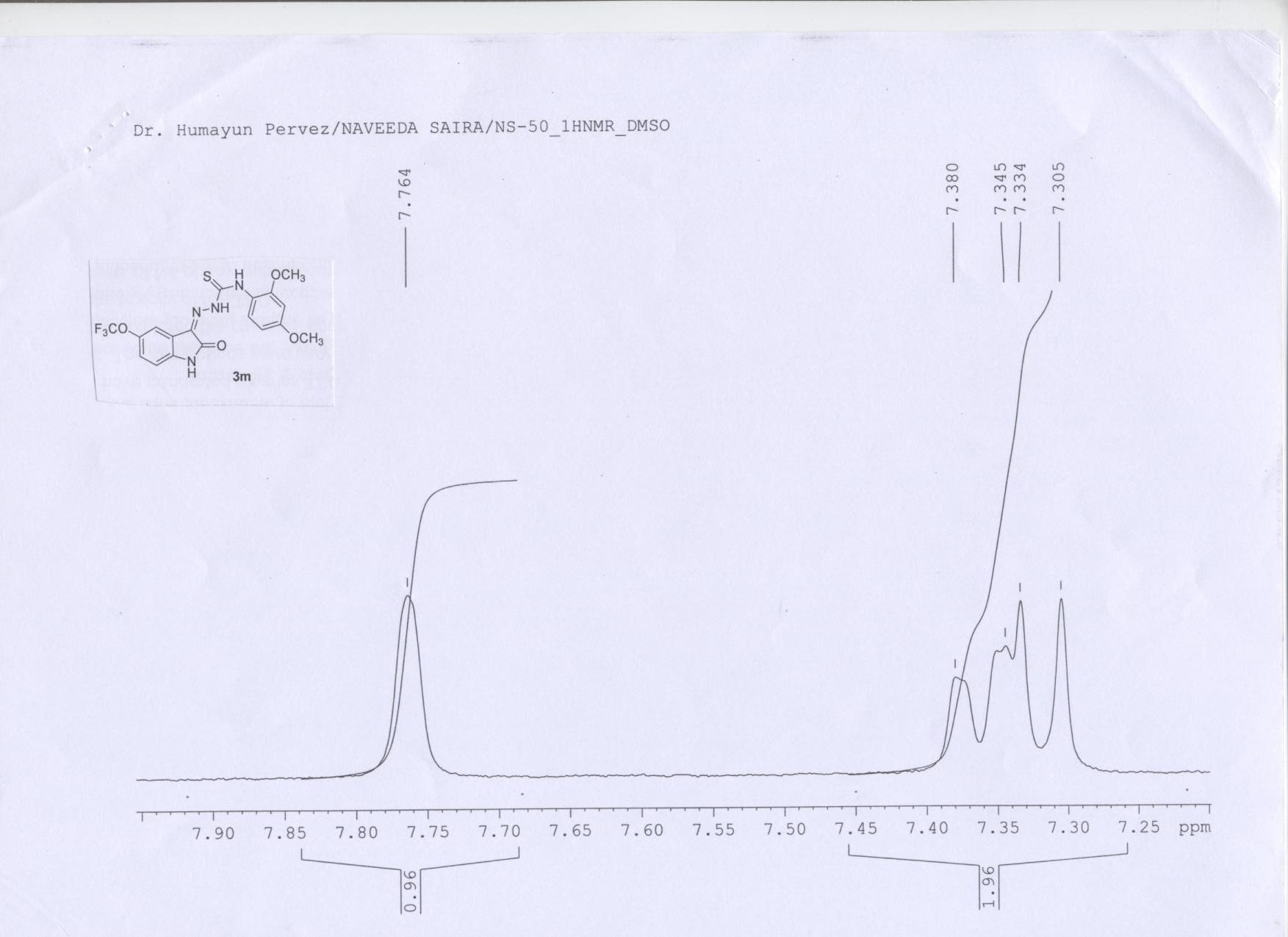

Supplement: Supplementary File 1 [file molecules-16-06408-s001.zip › Spectroscopy/NMR/3m2.jpg]

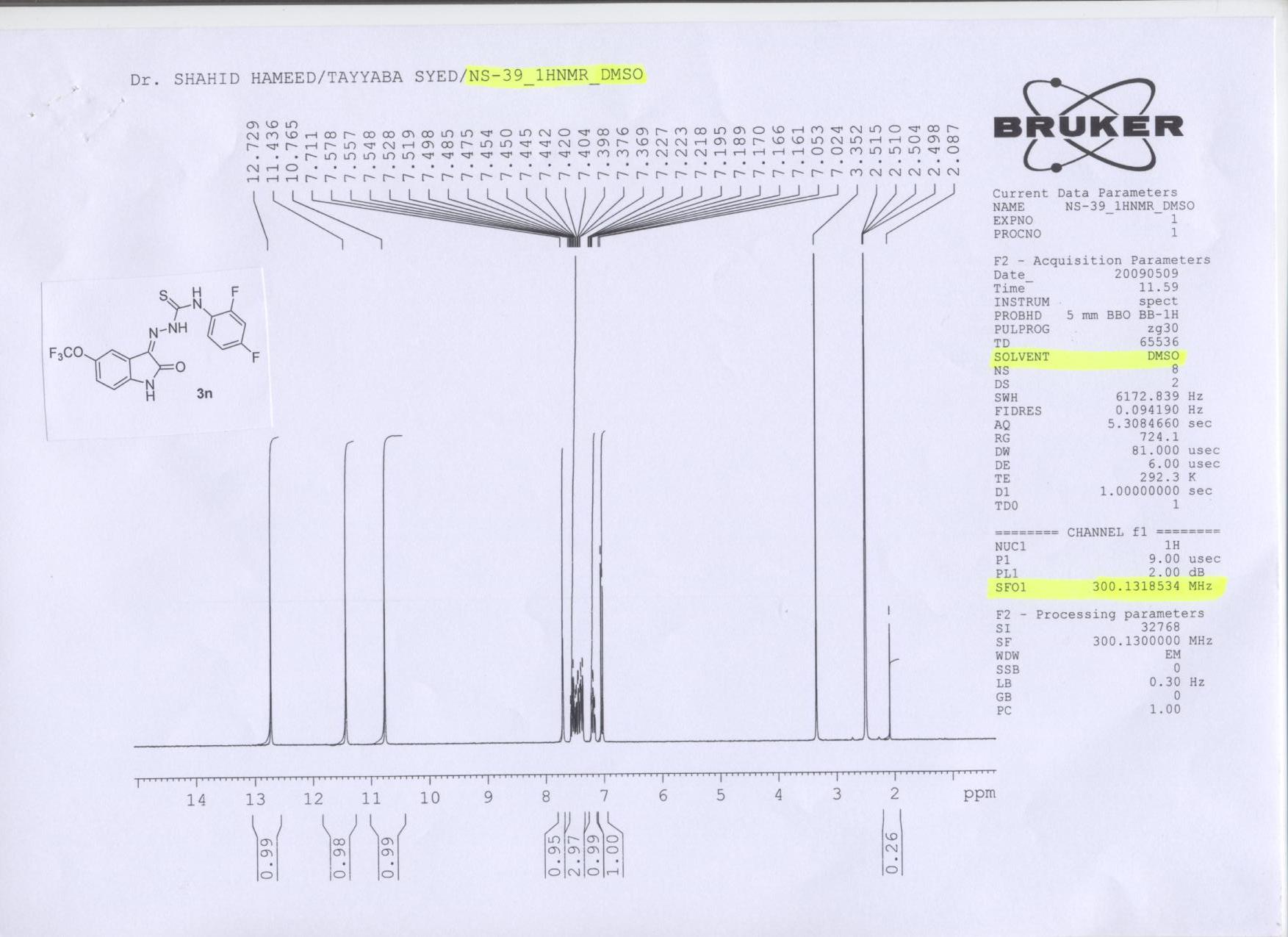

Supplement: Supplementary File 1 [file molecules-16-06408-s001.zip › Spectroscopy/NMR/3n.jpg]

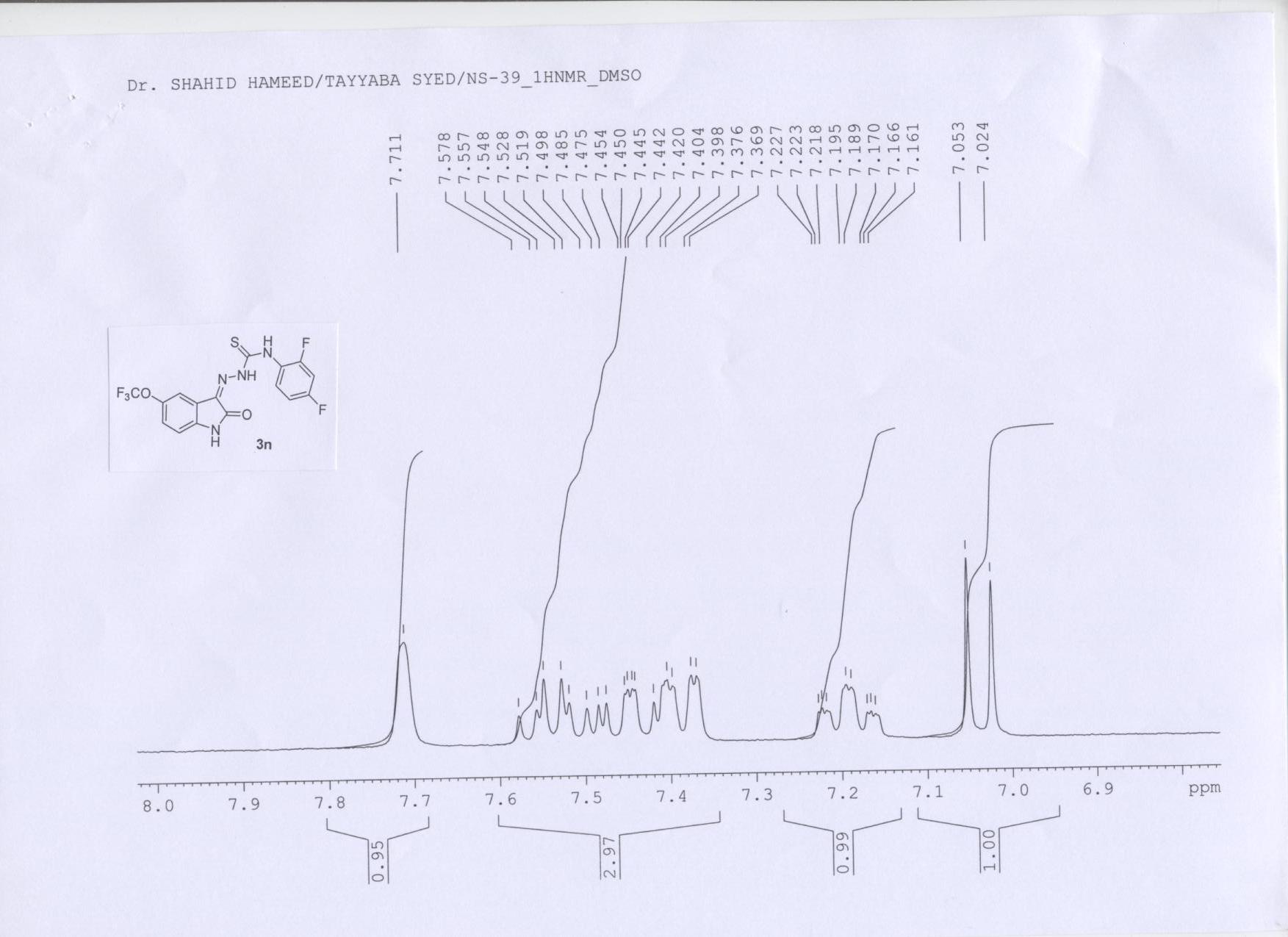

Supplement: Supplementary File 1 [file molecules-16-06408-s001.zip › Spectroscopy/NMR/3n1.jpg]

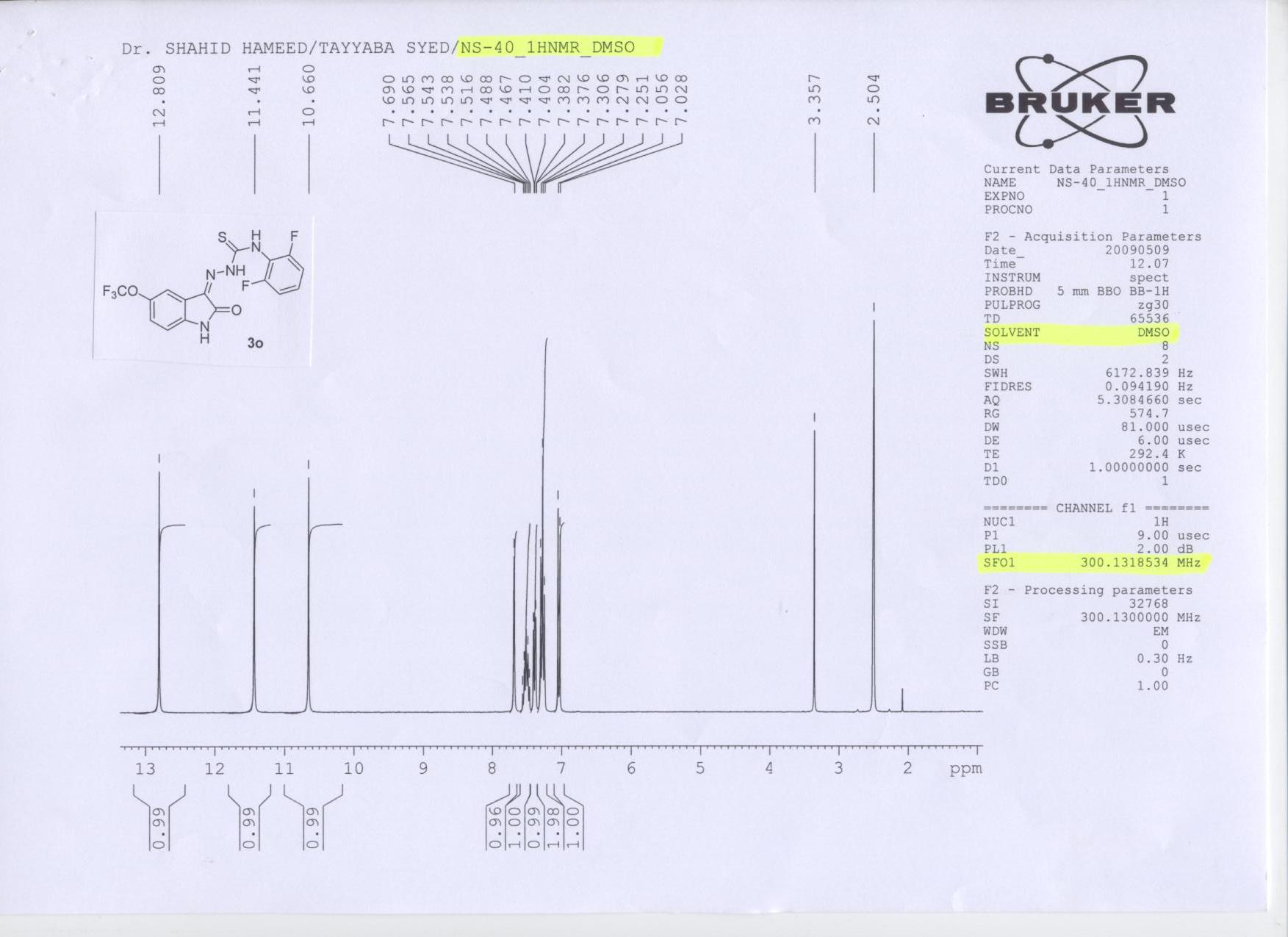

Supplement: Supplementary File 1 [file molecules-16-06408-s001.zip › Spectroscopy/NMR/3o.jpg]

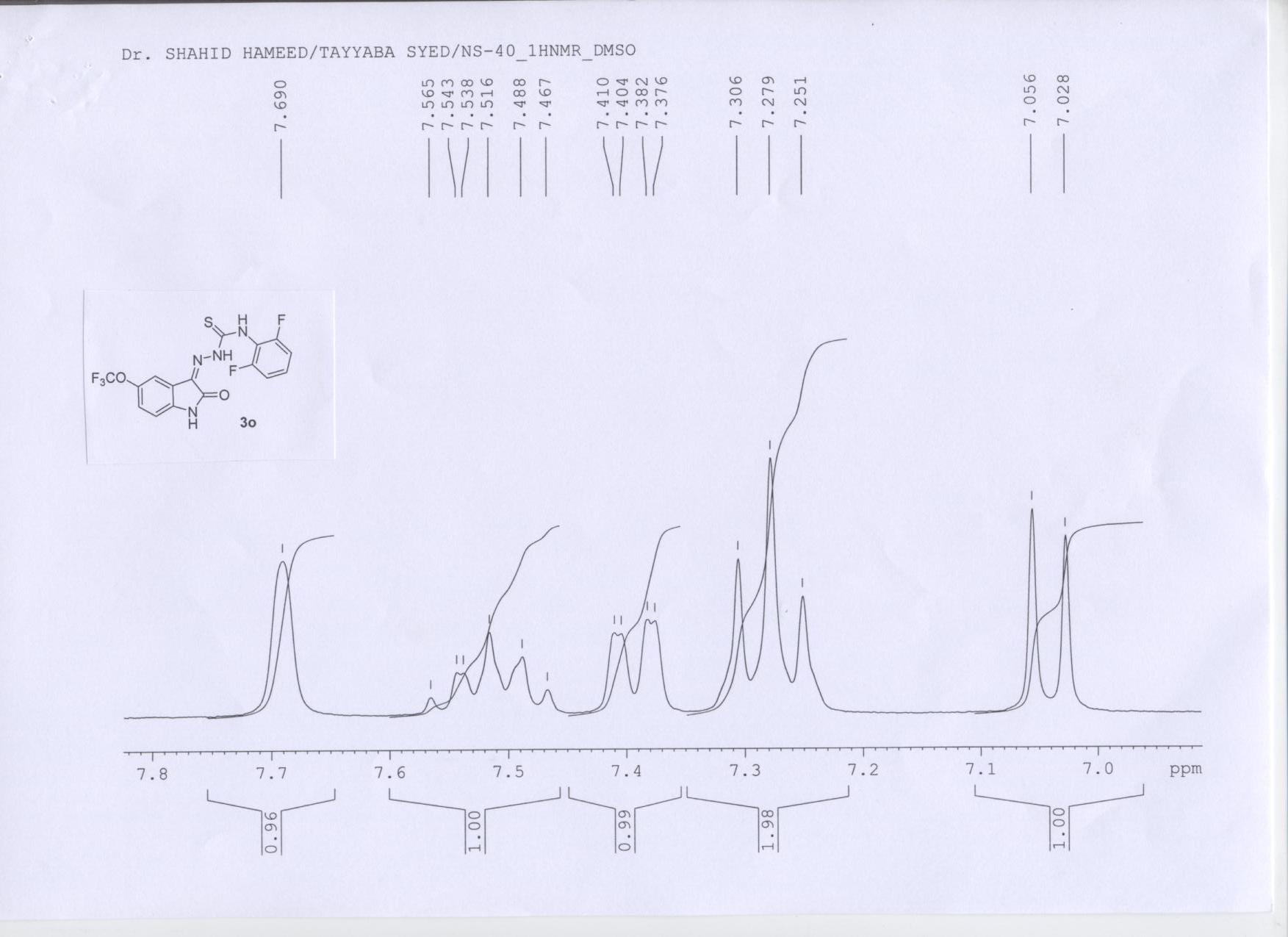

Supplement: Supplementary File 1 [file molecules-16-06408-s001.zip › Spectroscopy/NMR/3o1.jpg]

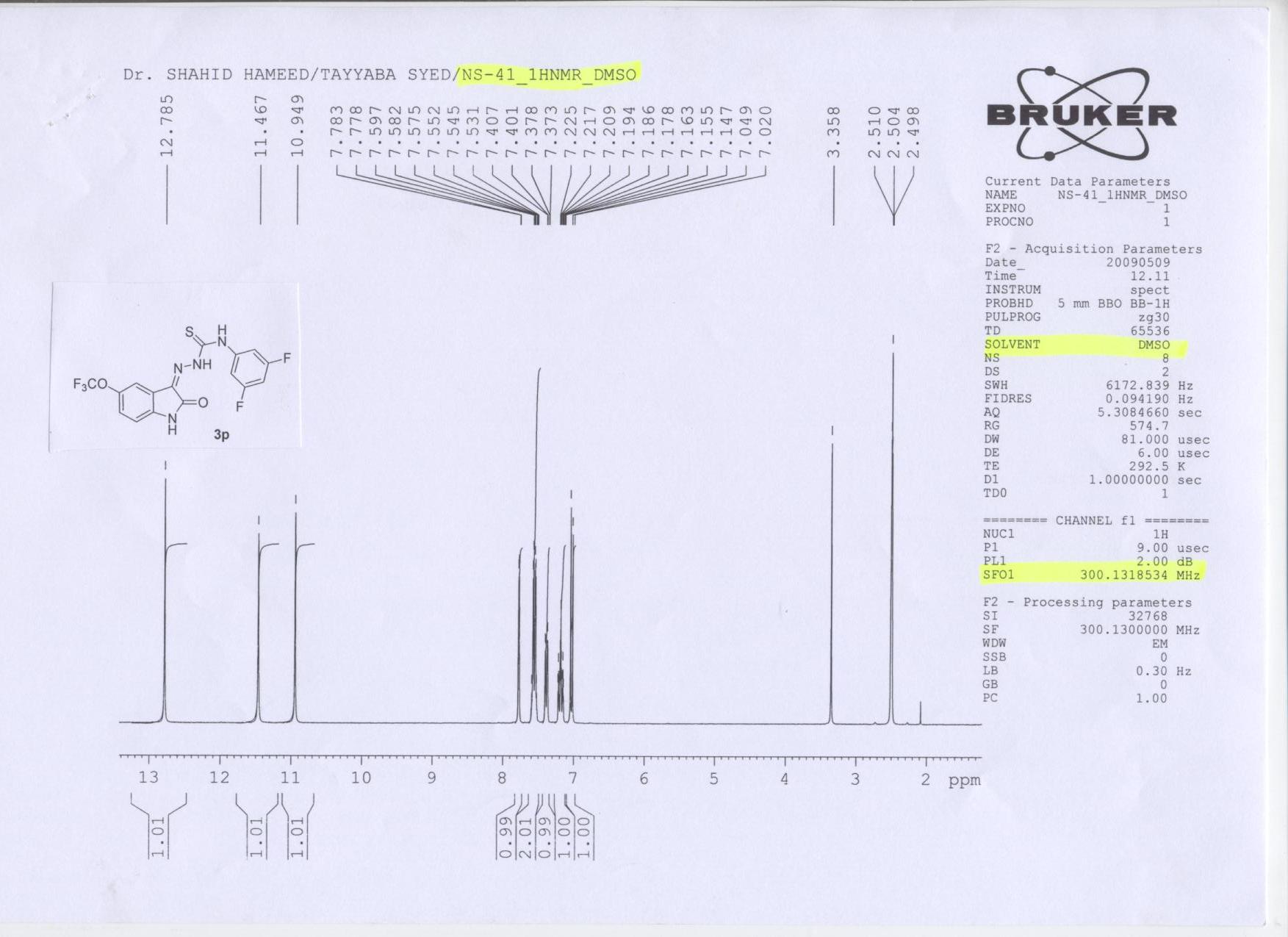

Supplement: Supplementary File 1 [file molecules-16-06408-s001.zip › Spectroscopy/NMR/3p.jpg]

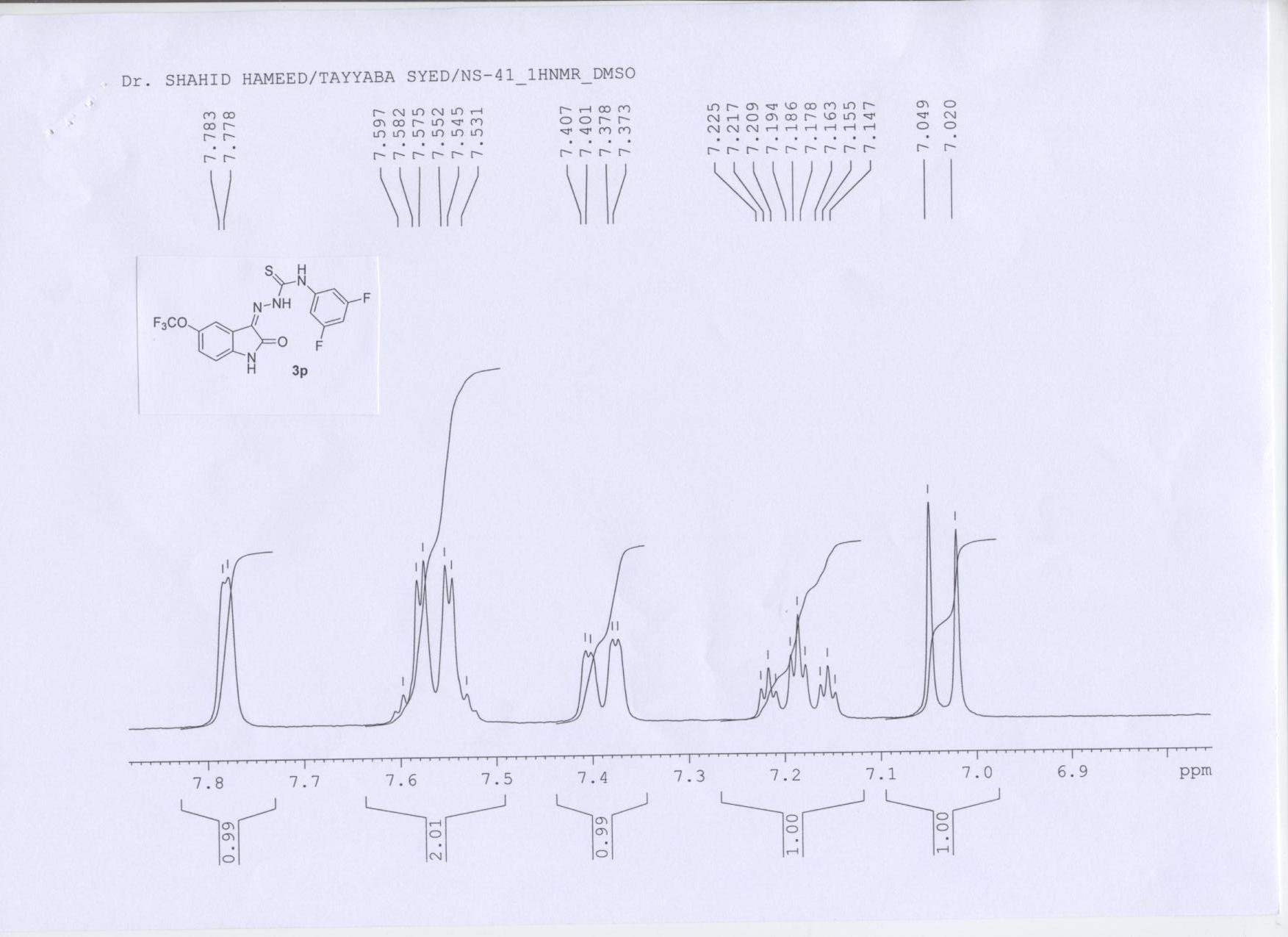

Supplement: Supplementary File 1 [file molecules-16-06408-s001.zip › Spectroscopy/NMR/3p1.jpg]

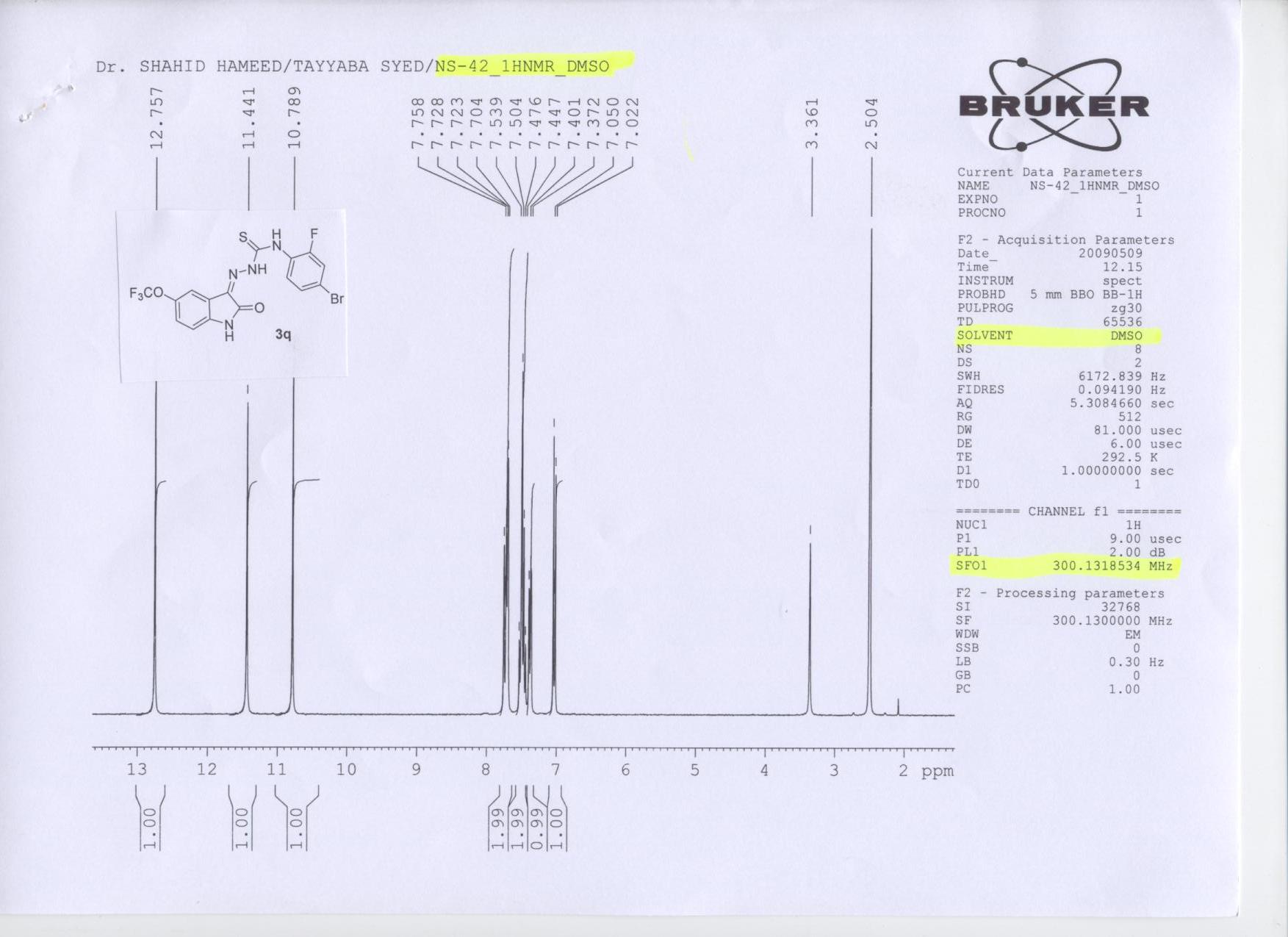

Supplement: Supplementary File 1 [file molecules-16-06408-s001.zip › Spectroscopy/NMR/3q.jpg]

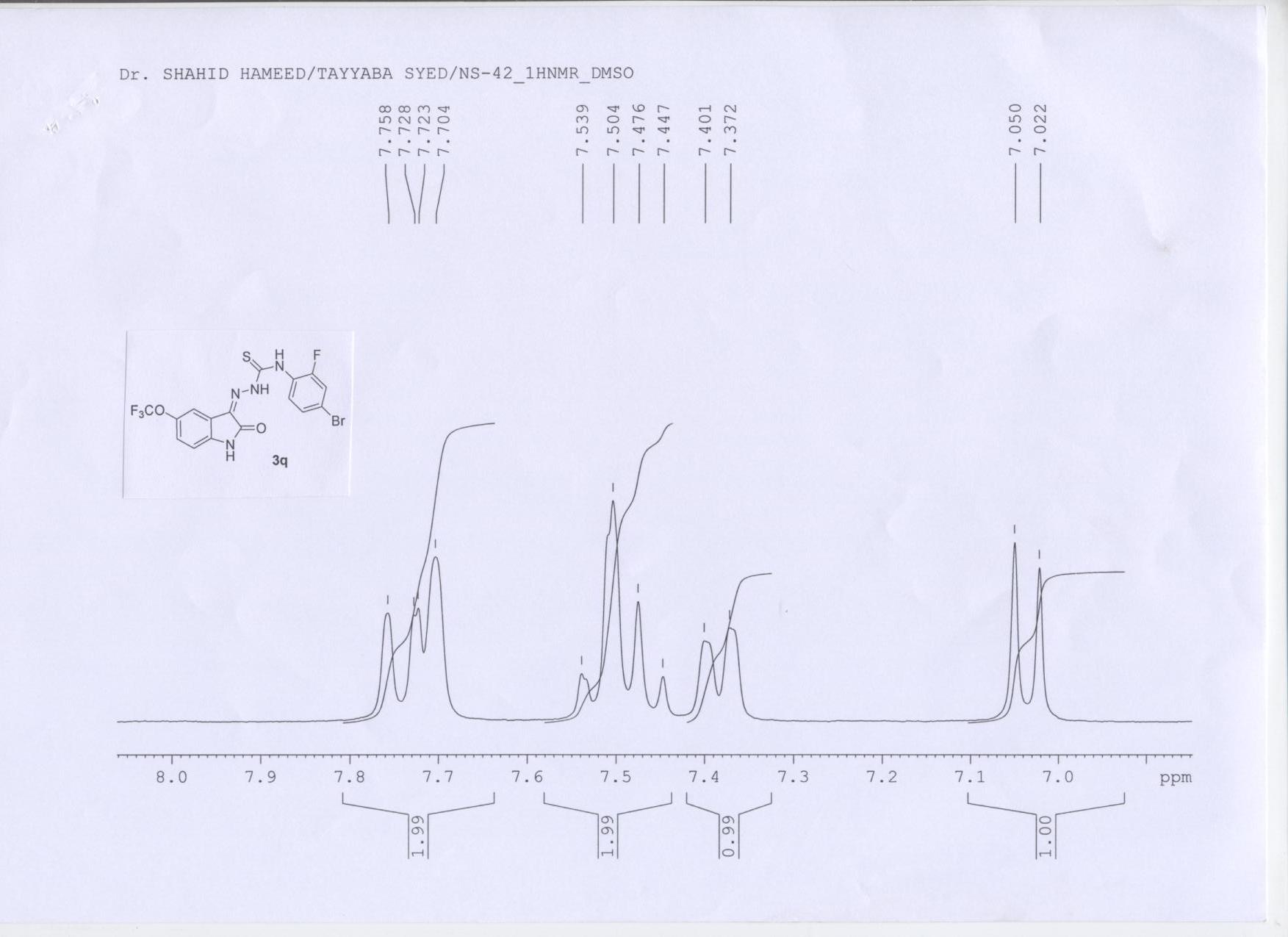

Supplement: Supplementary File 1 [file molecules-16-06408-s001.zip › Spectroscopy/NMR/3q1.jpg]

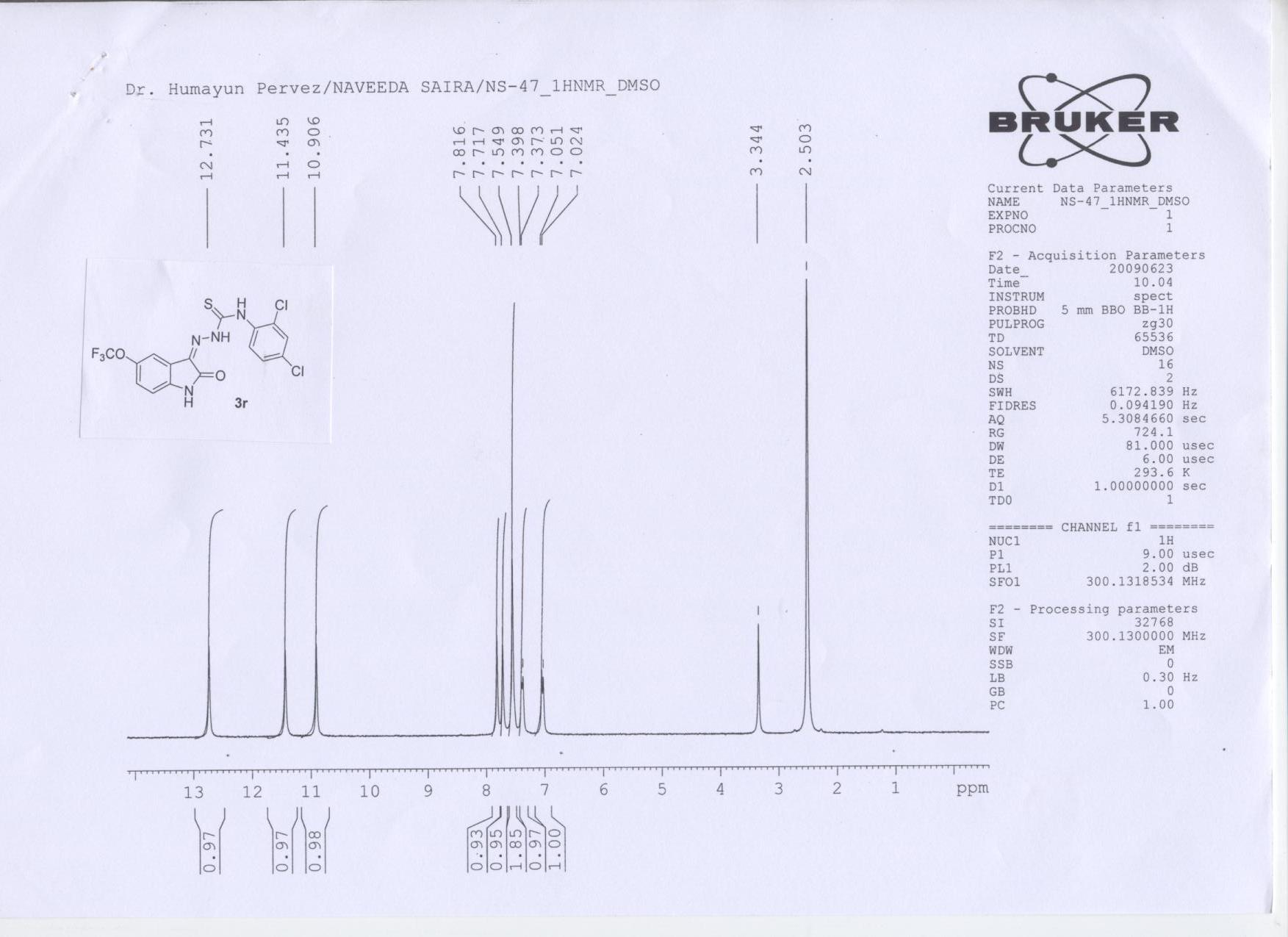

Supplement: Supplementary File 1 [file molecules-16-06408-s001.zip › Spectroscopy/NMR/3r.jpg]

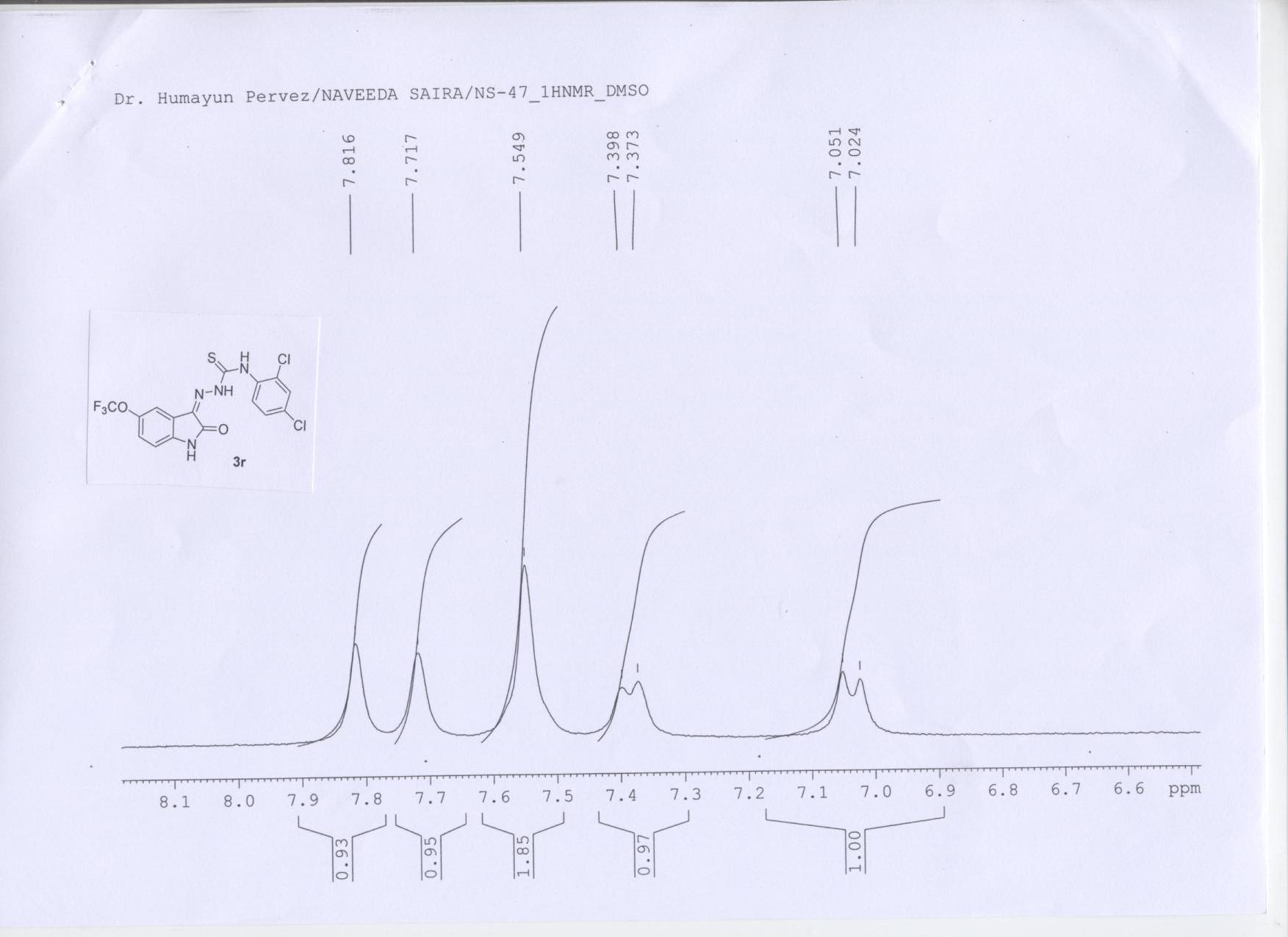

Supplement: Supplementary File 1 [file molecules-16-06408-s001.zip › Spectroscopy/NMR/3r1.jpg]

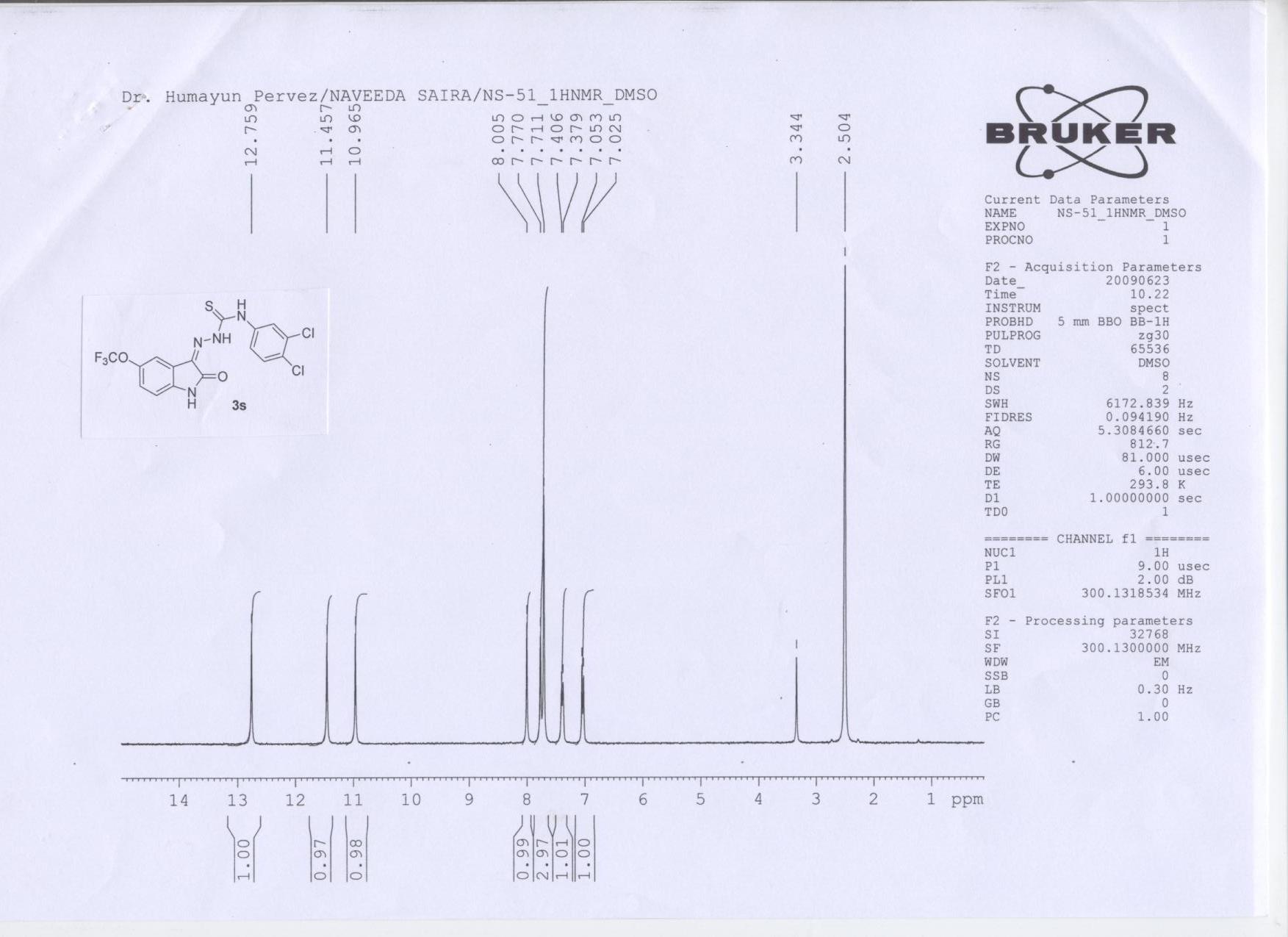

Supplement: Supplementary File 1 [file molecules-16-06408-s001.zip › Spectroscopy/NMR/3s.jpg]

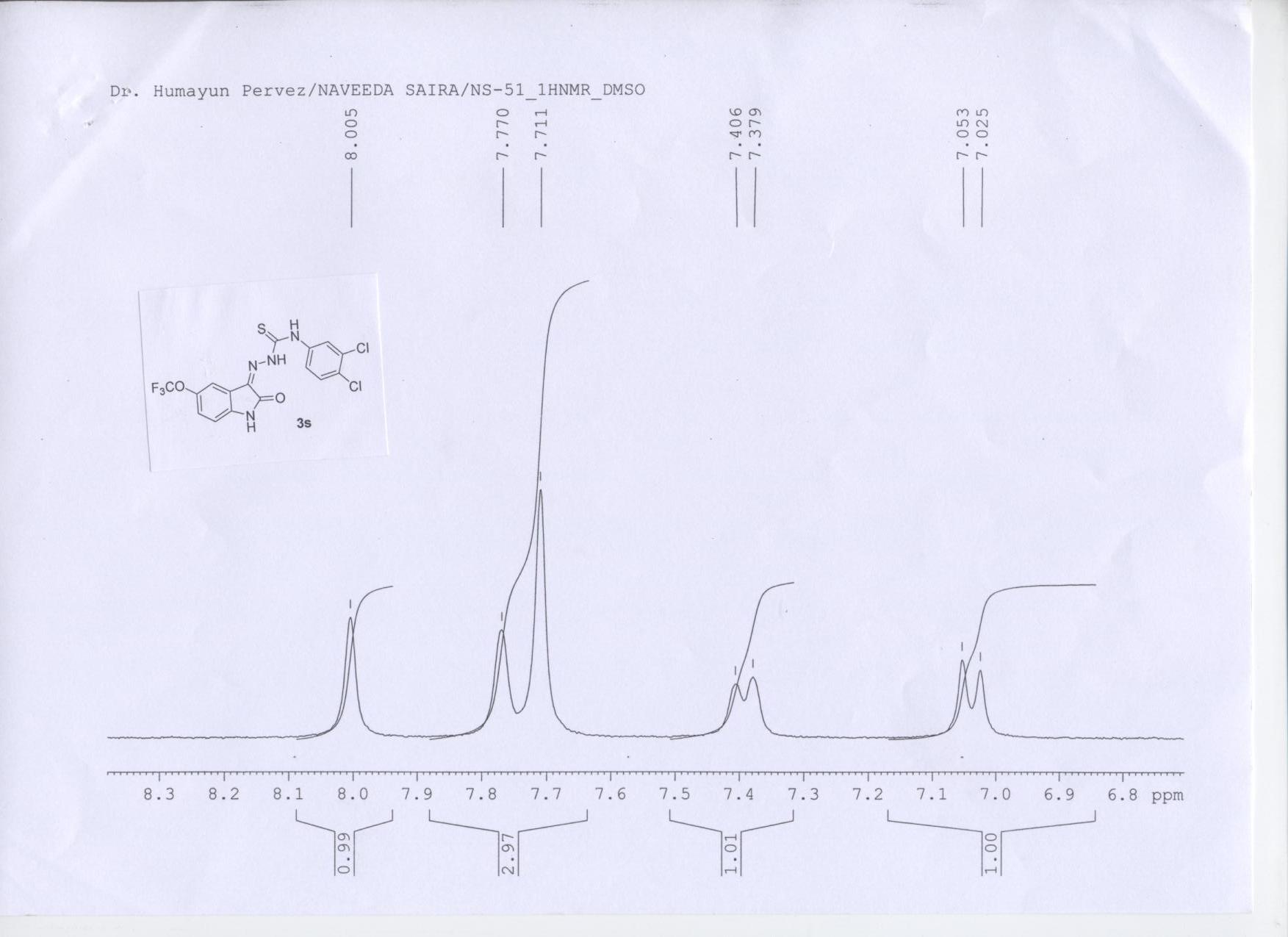

Supplement: Supplementary File 1 [file molecules-16-06408-s001.zip › Spectroscopy/NMR/3s1.jpg]

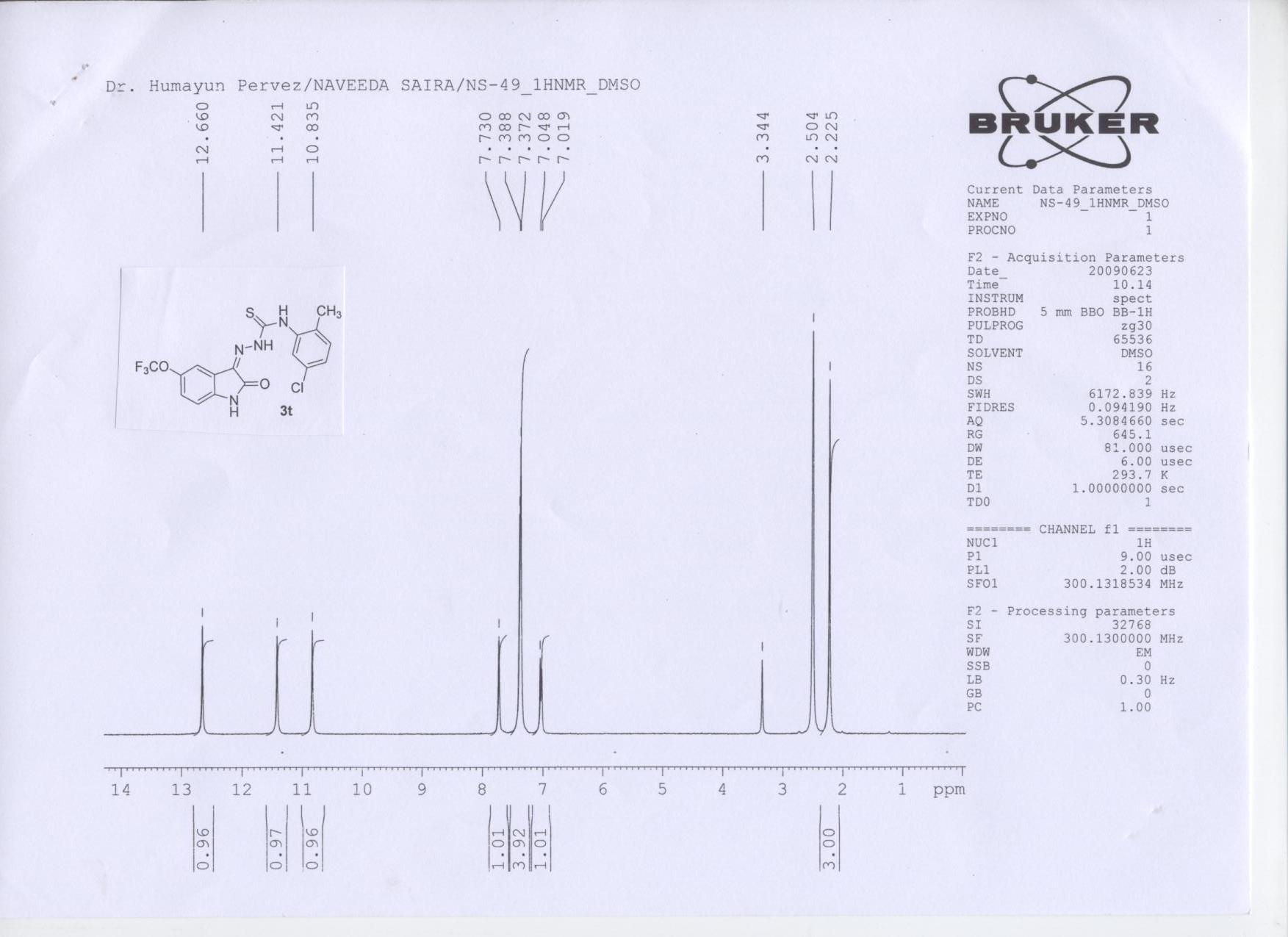

Supplement: Supplementary File 1 [file molecules-16-06408-s001.zip › Spectroscopy/NMR/3t.jpg]

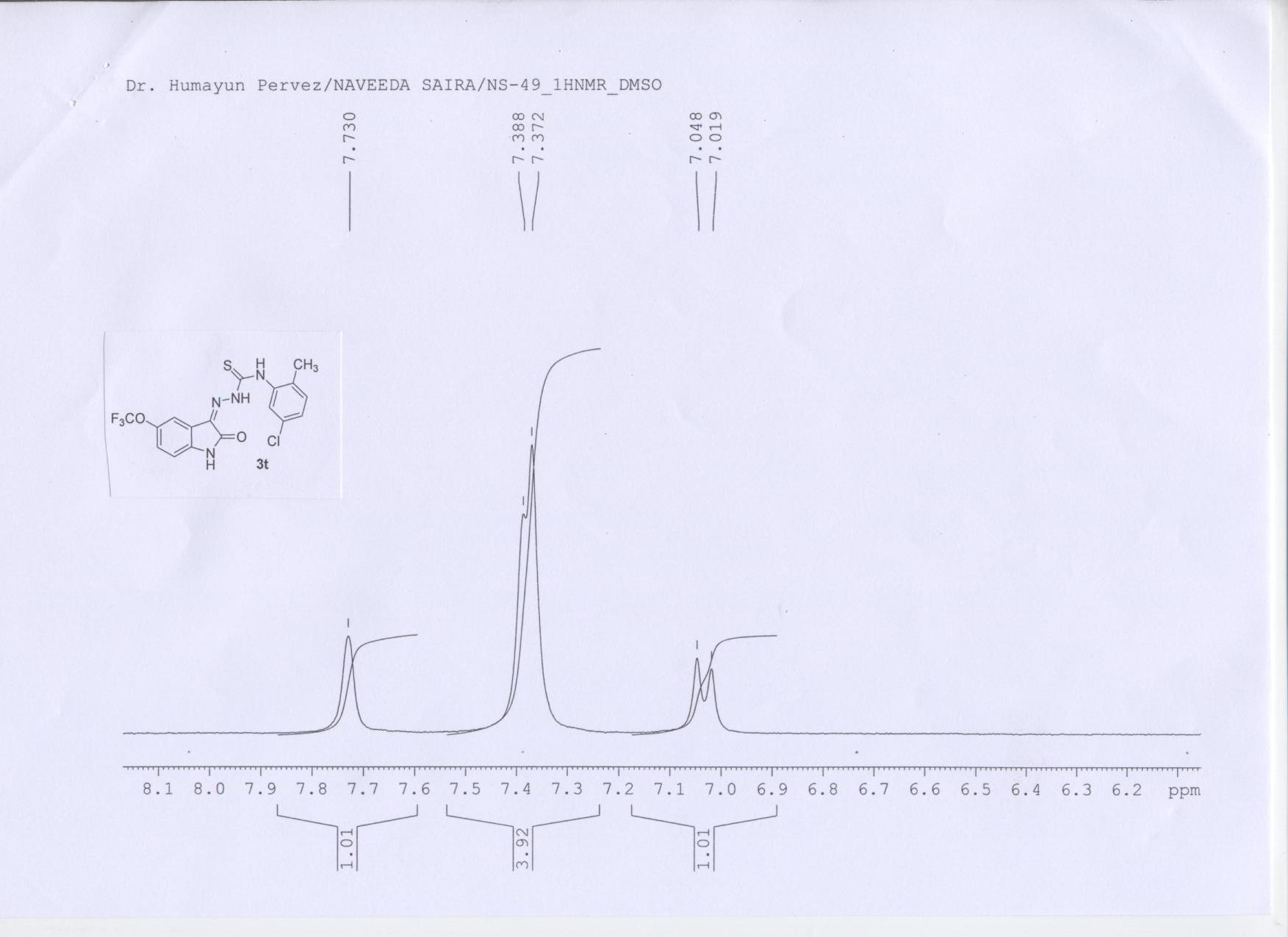

Supplement: Supplementary File 1 [file molecules-16-06408-s001.zip › Spectroscopy/NMR/3t1.jpg]

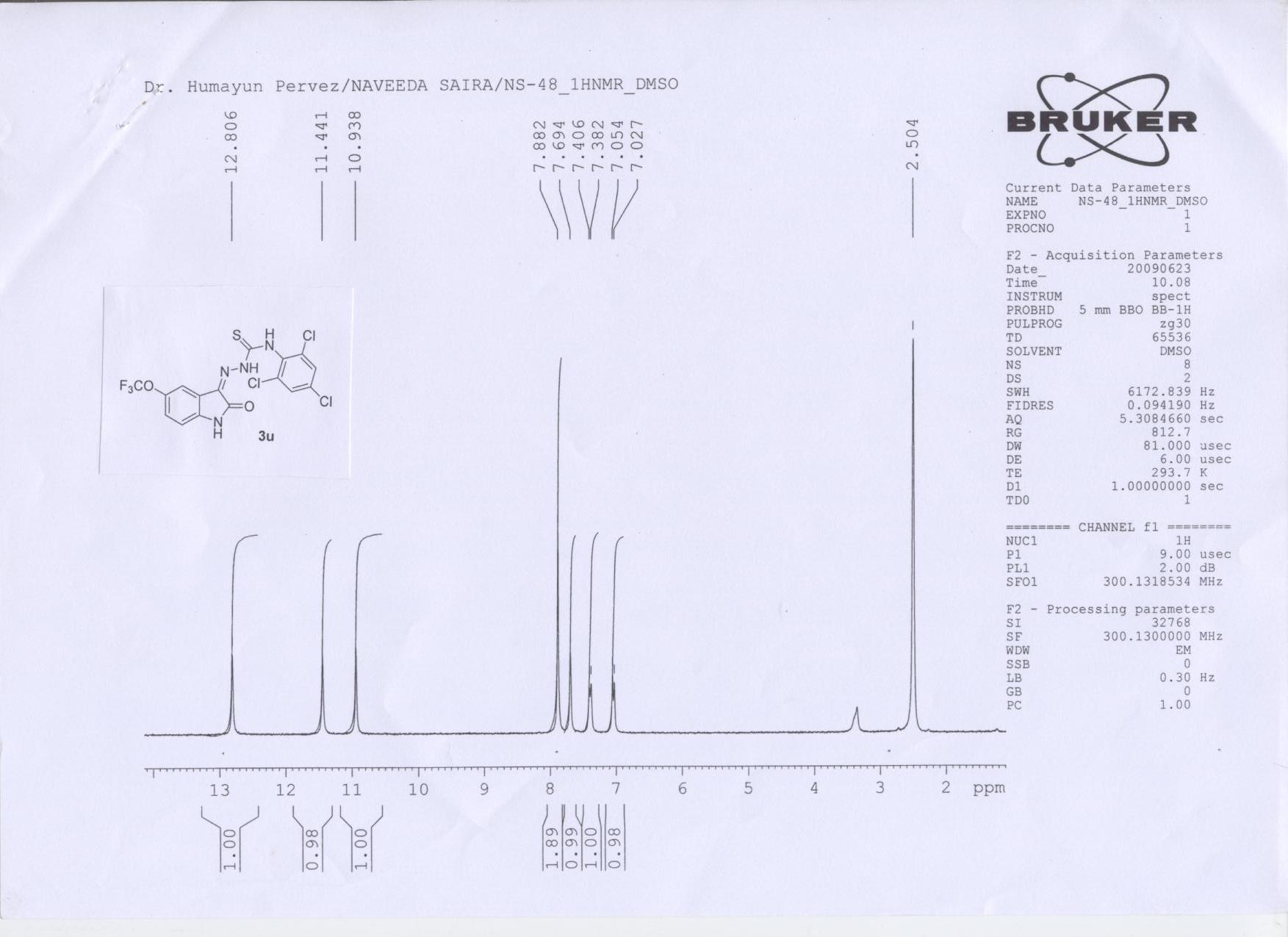

Supplement: Supplementary File 1 [file molecules-16-06408-s001.zip › Spectroscopy/NMR/3u.jpg]

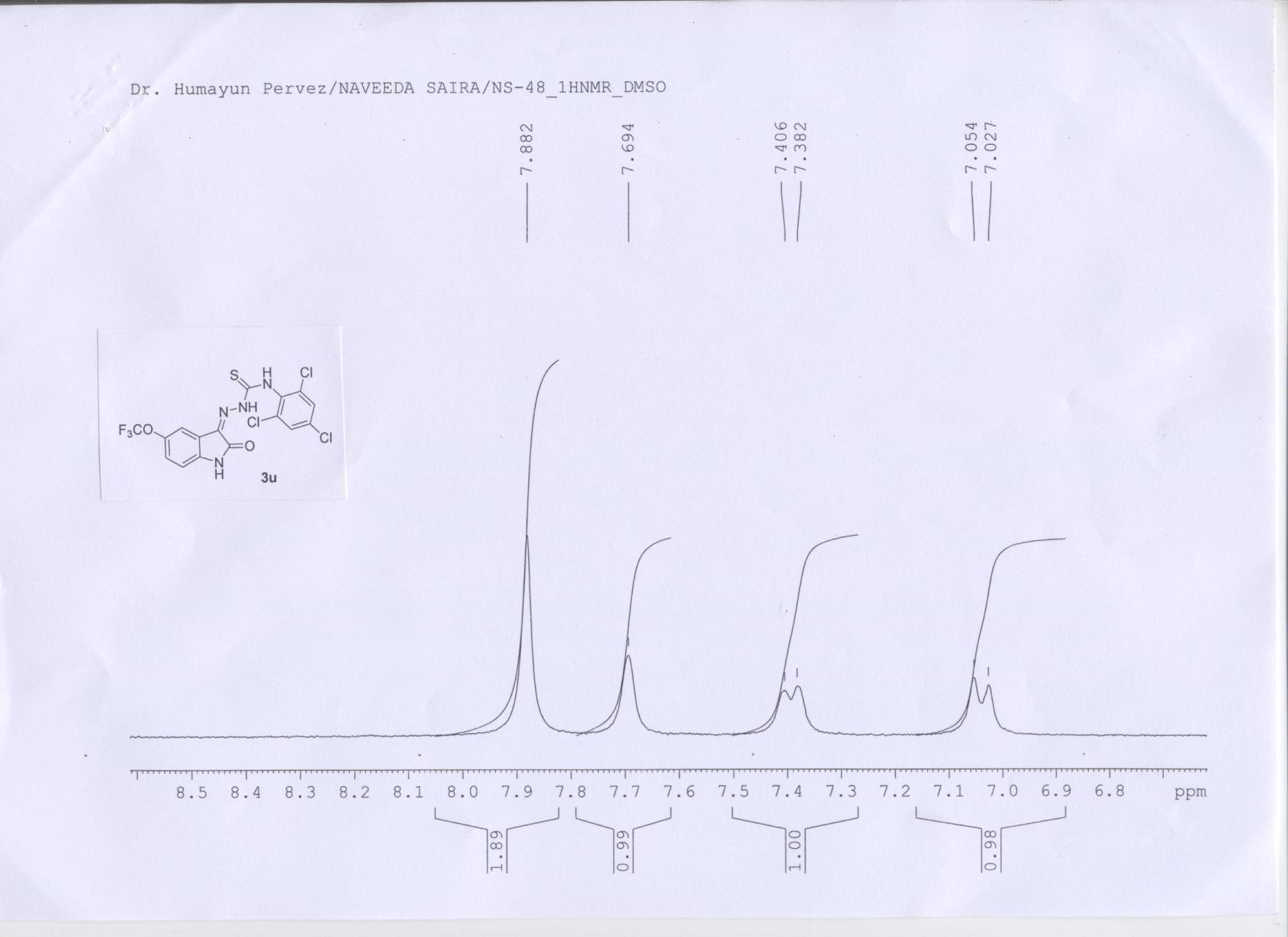

Supplement: Supplementary File 1 [file molecules-16-06408-s001.zip › Spectroscopy/NMR/3u1.jpg]
